# EDNRB‐dependent endothelin signaling reduces proliferation and promotes proneural‐to‐mesenchymal transition in gliomas

**DOI:** 10.1002/1878-0261.70223

**Published:** 2026-04-23

**Authors:** Donovan Pineau, Leonor Garcia, Hugo Arnold, Antonija Hanžek, Maialen Arrieta, Laurent R Gauthier, Christine Granotier‐Beckers, François D Boussin, Amaury Herbet, Marie Hautière, Valentin Asei‐Ceschino, Clémentin Jacques, Laura Brard, Thomas Harnois, Valérie Coronas, Bruno Constantin, Aurélien Chatelier, Jean Chemin, Serge Urbach, Martial Seveno, Szimonetta Hideg, Chantal Ripoll, Kasandra Aguilar‐Cázarez, Min Zheng, Guo‐Hao Huang, Sheng‐Qing Lv, Lei Zhang, Philippe Rondard, Laurent Prezeau, Jean‐Philippe Pin, Hugues Duffau, Luc Bauchet, Valérie Rigau, Franck Denat, Charles Truillet, Didier Boquet, Jean‐Philippe Hugnot

**Affiliations:** ^1^ Institut de Génomique Fonctionnelle Université de Montpellier, CNRS, INSERM France; ^2^ University of Bordeaux, CNRS, IBGC UMR5095 France; ^3^ Université Paris Cité and Université Paris‐Saclay, Inserm, CEA, Stabilité Génétique Cellules Souches et Radiations Fontenay‐aux‐Roses France; ^4^ Université Paris‐Saclay, CEA, DMTS, SPI France; ^5^ Institut Génétique Humaine Université de Montpellier, CNRS, INSERM France; ^6^ 4CS, Laboratory Channels and Connexins in Cancers and Cell Stemness, UR 22751 University of Poitiers France; ^7^ PReTI Laboratory, UR 24184 University of Poitiers France; ^8^ BioCampus Montpellier Université de Montpellier, CNRS, INSERM France; ^9^ Jinfeng Laboratory Chongqing China; ^10^ Neurosurgery Department Montpellier University Medical Center France; ^11^ Pathology Department Montpellier University Medical Center France; ^12^ Université Bourgogne, Europe, CNRS, UMR 6302 Dijon France; ^13^ Université Paris‐Saclay, CEA, CNRS, Inserm, BioMaps, Orsay France

**Keywords:** Diffuse gliomas, EDNRA, EDNRB, endothelin signaling, Proneural‐to‐Mesenchymal Transition, tumor cell plasticity

## Abstract

Diffuse gliomas are primary brain tumors including glioblastomas (GB), astrocytomas, and oligodendrogliomas, the latter two harboring IDH1 mutations and exhibiting slower progression. Gliomas display cellular plasticity, with transitions between astrocyte‐like, oligodendrocyte‐like, progenitor‐like, and mesenchymal‐like states driven by genetic alterations and microenvironmental signals. The proneural‐to‐mesenchymal transition (PMT), associated with increased malignancy, is tightly regulated by the tumor microenvironment, notably through cytokine signaling and non‐tumor cell interactions. Endothelins (ET‐1, ET‐2, ET‐3), vasoactive peptides mainly produced by vascular cells, signal through the G‐protein‐coupled receptors EDNRA and EDNRB and were previously suggested to promote glioma proliferation based on serum‐based models. Here, we revisited endothelin signaling using eleven serum‐free glioma lines and tumor samples. Multi‐omics and electrophysiological analyses identified EDNRB as the predominant receptor, enriched in astrocyte‐like cells, increased by BMPs or growth factor withdrawal, and repressed by interferons, IL‐6 family cytokines, endothelins, and Hippo/YAP signaling. EDNRA was confined to a perivascular tumor subpopulation and induced by Notch signaling selectively in GB. Functionally, endothelins reduced proliferation while promoting migration and PMT via EDNRB‐dependent Ca^2+^ signaling, ERK/STAT3 activation, and apamin‐sensitive SK2/SK3 potassium channel activity. Collectively these findings establish endothelin signaling as an important regulator of glioma cell plasticity and behavior.

## Graphical abstract

Glioma cells mainly express the endothelin receptor EDNRB, while EDNRA is restricted to a perivascular tumor subpopulation. Endothelin signaling reduces glioma cell proliferation while promoting migration and a proneural‐to‐mesenchymal transition associated with poor prognosis. This pathway activates Ca^2^^+^, K^+^, ERK, and STAT3 signalings and is regulated by BMP, inflammatory cytokines, Hippo/YAP1, Notch signaling, and hypoxia.

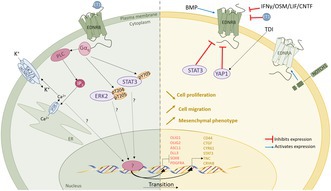

**Abbreviations**
AU: Arbitrary Unit
AC: Astrocyte‐like cells
ACTB: Actin Beta
Akt (PKB): Protein Kinase B
APOE: Apolipoprotein E
AQP4: Aquaporin 4
ASCL1: Achaete‐Scute Family BHLH Transcription Factor 1 (MASH1)
ASTRO: Astrocytomas
ATCC: American Type Culture Collection
ATRX: Alpha‐Thalassemia/Mental Retardation Syndrome X‐linked
BMP: Bone Morphogenetic Protein
BT: Brain Tumor
C/EBP: CCAAT‐Enhancer‐Binding Proteins
CAV1: Caveolin 1
CCN1/CYR61: Cellular Communication Network Factor 1/Cysteine‐Rich Angiogenic Inducer 61
CCN2/CTGF: Cellular Communication Network Factor 2/Connective Tissue Growth Factor
CCNB1: Cyclin B1
CCND1: Cyclin D1
CCND2: Cyclin D2
CD31: Cluster of Differentiation 31 (PECAM‐1, Platelet Endothelial Cell Adhesion Molecule 1)
CD44: Cluster of Differentiation 44 antigen
CDH5: Cadherin 5 (VE‐Cadherin)
CGGA: Chinese Glioma Genome Atlas
CHI3L1: Chitinase‐3‐Like Protein 1 (YKL40)
CHO: Chinese Hamster Ovary
CLU: Clusterin
CNTF: Ciliary Neurotrophic Factor
CRYAB: αB‐crystallin
CV: Coefficient of Variation
Da: Dalton
DEG: Differentially Expressed Gene
DEP: Differentially Expressed Protein
EDNRA: Endothelin Receptor Type A
EDNRB: Endothelin Receptor Type B
EdU: 5‐ethynyl‐2′‐deoxyuridine
EGF: Epidermal Growth Factor
EGFR: Epidermal Growth Factor Receptor
EMT: Epithelial‐to‐Mesenchymal Transition
ERK1: Extracellular Signal‐Regulated Kinase 1 (MAPK3)
ERK2: Extracellular Signal‐Regulated Kinase 2 (MAPK1)
ET‐1: Endothelin 1
ET‐2: Endothelin 2
ET‐3: Endothelin 3
FACS: Fluorescence‐Activated Cell Sorting
FC: Fold Change
FDR: False Discovery Rate
FGF: Fibroblast Growth Factor
FRET: Fluorescence Resonance Energy Transfer
GB: Glioblastomas (former GBM)
GF: Growth Factor
GFAP: Glial Fibrillary Acidic Protein
GFP: Green Fluorescent Protein
GJA1: Gap Junction Protein Alpha 1
GPCR: G‐Protein‐Coupled Receptor
GSEA: Gene Set Enrichment Analysis
HIGD1B: Hypoxia Inducible Domain Family Member 1B
HOPX: Homeodomain‐Only Protein X
HTRF: Homogeneous Time‐Resolved Fluorescence
HUVEC: Human Umbilical Vein Endothelial Cells
IBAQ: Intensity‐Based Absolute Quantification
IDH1: Isocitrate Dehydrogenase 1
IF: Immunofluorescence
IFN: Interferon
IP1: Inositol Monophosphate
IP3: Inositol Triphosphate
JNK: c‐Jun N‐terminal Kinase
KCNN2/SK2: Potassium Calcium‐Activated Channel Subfamily N Member 2/Small Conductance
KCNN3/SK3: Potassium Calcium‐Activated Channel Subfamily N Member 3/Small Conductance
KDR: Kinase Insert Domain Receptor (VEGFR2)
LATS1/2: Large Tumor Suppressor Kinase 1/2
LGG: Low‐Grade Gliomas
LIF: Leukemia Inhibitory Factor
MACS: Magnetic‐Activated Cell Sorting
MAPK: Mitogen‐Activated Protein Kinase
MES: Mesenchymal state
MHC: Major Histocompatibility Complex
MKI67: Marker of proliferation Ki‐67
mRNA: Messenger Ribonucleic Acid
MSD: Mean Square Displacement
MYH11: Myosin Heavy Chain 11
NES: Normalized Enrichment Score
NFκB: Nuclear Factor κB
NICD: Notch Intracellular Domain
O: Oligodendrogliomas (ODG)
OLIG1: Oligodendrocyte Lineage Transcription Factor 1
OLIG2: Oligodendrocyte Lineage Transcription Factor 2
OPC: Oligodendrocyte Progenitor Cells
OSM: Oncostatin M
P38: p38 Mitogen‐Activated Protein Kinase
PBS: Phosphate‐Buffered Saline
PCNA: Proliferating Cell Nuclear Antigen
PDGFRA: Platelet‐Derived Growth Factor Receptor Alpha
PDGFRB: Platelet‐Derived Growth Factor Receptor Beta
PDL: poly‐D‐Lysine
PI3K/AKT: Phosphoinositide 3‐Kinase/Protein Kinase B
PLCβ/Cγ: Phospholipase C β/γ
PMT: Proneural‐to‐Mesenchymal Transition
PolyHEMA: Poly, (2‐hydroxyethyl methacrylate)
pSMAD1/5: Phosphorylated SMAD1/5
PTPRZ1: Protein Tyrosine Phosphatase Receptor Type Z1
RB49: Rendomab‐B49
REMBRANDT: Repository for Molecular Brain Neoplasia Data
RGS5: Regulator of G Protein Signaling 5
RhoA: Ras Homolog Family Member A
RNA‐seq: RNA Sequencing
ROCK: Rho‐Associated Coiled‐Coil Containing Protein Kinase
ROS: Reactive Oxygen Species
RT‐qPCR: Reverse Transcription Quantitative Polymerase Chain Reaction
SC: Single Cell
SD: Standard Deviation
SDF‐1/CXCL12: Stromal Cell‐Derived Factor 1 (CXCL12)
SEM: Standard Error of the Mean
Smooth Muscle: ACTA2 Actin Alpha 2
SOX2/4/8/9/10: SRY‐Box Transcription Factors 2/4/8/9/10
SPARC: Secreted Protein, Acidic and Rich in Cysteine
STAT3: Signal Transducer and Activator of Transcription 3
STR: Short Tandem Repeat
TAZ: Transcriptional Coactivator with PDZ‐Binding Motif (WWTR1)
TCGA: The Cancer Genome Atlas
TGFβ: Transforming Growth Factor Beta
TME: Tumor Microenvironment
TMT: Tandem Mass Tag
TNC: Tenascin C
TPM: Transcripts Per Million
TWIST1/2: Twist Family BHLH Transcription Factors 1/2
UMAP: Uniform Manifold Approximation and Projection
VIM: Vimentin
WB: Western Blot
WHO: World Health Organization
YAP: Yes‐Associated Protein
YFP: Yellow Fluorescent Protein

## 1 Introduction

Diffuse gliomas are the most common type of malignant primary tumors of the central nervous system and remain incurable [1, 2]. Mounting evidence suggests that gliomagenesis originates in adult brain neural stem cells or glial progenitors, which acquire oncogenic alterations and escape normal differentiation cues [3]. Clinically, diffuse gliomas are divided into three major entities: glioblastomas (GB)—the most aggressive form—and astrocytomas and oligodendrogliomas [4, 5, 6]. According to the 2021 WHO classification [4], glioblastomas are defined as IDH‐wild‐type tumors, whereas astrocytomas and oligodendrogliomas are characterized by recurrent IDH1 mutations, whose aberrant enzymatic activity rewires cellular metabolism and reshapes the epigenome, ultimately impairing lineage commitment and promoting tumor progression [7].

Single‐cell RNA sequencing has revealed profound intratumoral heterogeneity across diffuse gliomas [8]. In IDH‐mutant astrocytomas and oligodendrogliomas, tumor cells transition among astrocyte‐like, oligodendrocyte‐like, and neural stem cell‐like states [9, 10]. The signaling pathways regulating these lineage identities are beginning to be elucidated. In lower‐grade gliomas, we and others have shown that Notch and BMP signaling drive cells toward a quiescent or slowly proliferative, astrocytic‐like phenotype [11, 12]. Of note, glioblastomas contain an additional mesenchymal‐like population whose abundance varies between tumors [13, 14]. The relative proportions of oligodendrocyte‐like and mesenchymal‐like cells contribute to the classification of GB into proneural and mesenchymal subtypes, respectively [15]. The proneural state broadly reflects neural and glial developmental programs, characterized by *OLIG2* and *SOX2* expression and is associated with higher proliferative and differentiation potential. In contrast, the mesenchymal state is defined by extracellular matrix– and inflammation‐related genes (*CD44*, *CHI3L1*), enhanced invasiveness, therapy resistance, and activation of NF‐κB and TGF‐β signaling pathways [16]. Frequent proneural‐to‐mesenchymal transitions (PMTs) are observed, particularly upon recurrence, and are promoted by therapeutic stress (irradiation, temozolomide), hypoxia, inflammation, and neuronal activity [17]. This transition toward a mesenchymal‐like state is orchestrated by key transcription factors—including STAT3, C/EBPβ, NF‐κB, Notch, and YAP/TAZ—and can be initiated by pro‐inflammatory cytokines such as oncostatin M (OSM) and leukemia inhibitory factor (LIF), which could activate the STAT3 pathway and are primarily secreted by immune cells in the tumor microenvironment (TME) [18, 19].

Beyond supplying oxygen and nutrients to cancer cells, vascular cells in the TME also play an essential role in shaping tumor cell identity [20, 21]. For instance, they modulate glioma cell behavior through activation of the Notch and YAP1 signaling pathways [20, 22]. Blood vessels additionally produce bone morphogenetic proteins (BMPs), which likely contribute further to the modulation of lineage states in glioma [23, 24, 25]. Another vascularly linked pathway of interest is endothelin signaling. Endothelins (ET‐1, ET‐2, ET‐3) are cytokine‐like peptides synthesized as ~212‐amino acid preproendothelins that undergo sequential proteolytic processing to yield the mature, bioactive peptides [26]. ET‐1 is a 21‐amino acid peptide identified in 1988 as the most potent endogenous vasoconstrictor [27]. ET‐2 and ET‐3 isoforms differ from ET‐1, the most abundant, by 2 and 6 amino acids, respectively, resulting in subtle changes to peptide conformation and different binding affinities for endothelin receptors [28, 29, 30]. Brain endothelial cells are the predominant source of ET‐1, but under pathological or stress conditions, reactive astrocytes, activated microglia, and subsets of neurons also produce ET‐1 [31]. ET‐2 appears chiefly astroglial, whereas ET‐3 is enriched in choroid‐plexus and circumventricular neurons [26].

Beyond their canonical role in regulating vascular tone, endothelins have also emerged as context‐dependent modulators of proliferation, migration, and differentiation in astrocytes, neural stem cells and multiple cancers [31, 32]. Endothelins signal through two G‐protein‐coupled receptors in humans: the endothelin receptor type A (EDNRA) and type B (EDNRB), which share 59% amino acid identity [33]. EDNRA exhibits preferential binding to ET‐1 and ET‐2 (≈100‐fold) over ET‐3 (ET‐1 ≥ ET‐2 » ET‐3), whereas EDNRB binds all three endothelins with equal affinity [33]. While EDNRA displays more ubiquitous expression, EDNRB shows a more restricted pattern, with highest levels in the kidneys and in the brain [26, 31]. In the brain, EDNRB is highly enriched in astrocytes [34] and glial progenitors [35], and is also expressed in selected neuronal populations [36]. By contrast, EDNRA in the brain is predominantly expressed by vascular smooth muscle cells and pericytes, where it regulates vascular tone and reactivity [26, 37]. Upon activation by endothelins, EDNRA and EDNRB signal through promiscuous G‐protein coupling, predominantly engaging Gαq/11‐, Gαi/o‐, and Gα12/13‐dependent cascades that activate PLCβ–Ca^2+^/PKC, MAPK (ERK, JNK, p38), PI3K/AKT, and RhoA/ROCK pathways, thereby regulating ionic conductances, cytoskeletal dynamics, and transcriptional programs controlling cellular plasticity, proliferation, and migration [29, 31, 38].

The presence and impact of endothelins on gliomas have been explored in several studies [39, 40, 41, 42], generally reporting pro‐proliferative effects [43, 44, 45]. However, these studies mainly used serum‐cultured glioma cell lines, such as rat C6 and human U87, conditions that profoundly alter glioma phenotypes and activate multiple signaling pathways, thereby failing to accurately recapitulate the molecular and functional properties of gliomas [46, 47, 48]. To address this limitation, we undertook a comprehensive reassessment of endothelin signaling in gliomas using a panel of eleven well‐characterized, serum‐free patient‐derived cell lines representing glioblastomas, IDH1‐wt and IDH1‐mutant oligodendrogliomas and astrocytomas, complemented by analyses of primary tumor specimens. Through an integrative approach combining multi‐omics and electrophysiological assays, we re‐evaluate the expression and regulation of EDNRA and EDNRB, their downstream signaling pathways, and the functional responses of glioma cells to endothelin stimulation.

## 2 Material and methods

Additional information on tumor microarrays (TMAs), public glioma datasets (bulk and single‐cell RNA‐seq), single‐cell RNA‐seq analysis of the LGG275 cell line, and spatial transcriptomics analysis of glioblastoma samples is provided in Supplementary File Data S1.

### 2.1 Reagents, QPCR primers, and antibodies

References for all products used in this study are provided in the Table S1,S5,S6.

### 2.2 Patient samples

Tumor samples were obtained from the Neurology fresh tissue collection of the Montpellier University Hospital Biological Resources Center (Gui de Chauliac, CRB@chu-montpellier.fr; Project No. RECH/P722/1‐5) from November 2014 to May 2023 with informed patient consent. The experiments were undertaken with the understanding and written consent of each subject and were approved by the tumor biobank's scientific council on August 28, 2023. The study was conducted according to the guidelines of the Declaration of Helsinki and approved by the Institutional Review Board of CHU Hospital of Montpellier (IRB ID: 202000583, 198711). Glioma grading was performed by an experienced neuropathologist (Prof. V. Rigau) according to the 2021 WHO classification [4], based on both molecular alterations (*IDH1*, *ATRX*, *TP53*, 1p/19q) and histopathological criteria (See Table S2 for information on patient specimens included in this study). For Tumor microarrays (TMAs), each TMA contains duplicate 1 mm tissue cores representing characteristic regions of the tumors, as summarized in Table S3 (see Supporting Information).

### 2.3 Glioma cell lines

Astrocytoma cell lines (LGG85, LGG275, LGG336, LGG349) were generated in‐house from explant cultures of surgical resections performed at Montpellier University Hospital (CHU Gui de Chauliac), following the procedures previously described [49]. The characterization of IDH1‐mutant LGG275 and LGG85 lines has been reported [11], [50]. Detailed molecular characterization of LGG336 (IDH1‐mutant) and LGG349 (IDH1‐wild type) will be reported elsewhere [51]. Oligodendroglioma cell lines included BT138, BT237 [52], BT054, and BT088 [53, 54]. Glioblastoma stem‐like cell lines (Gb4, Gb5, Gb7, Gb21) were established and characterized as described previously [55], [56]. A complete list of cell lines and key features is provided in Table S4. U87 cell line (RRID: CVCL_0022) and T98G glioblastoma cell lines (RRID: CVCL_0556) (gift from Dr N.Laguette' laboratory, IGH, Montpellier) were cultured with serum according to the guidelines provided in the ATCC catalog.

### 2.4 Cell culture conditions

Glioma cell lines were maintained at 37 °C in a humidified incubator with 5% CO_2_ in serum‐free DMEM/F12 (#21331046, Life Technologies (Thermo Fisher Scientific), Carlsbad, CA, USA) supplemented with L‐glutamine (2 mm; #25030024, Thermo Fisher, Waltham, MA, USA), N‐2 supplement (1×; #17502048, Life Technologies), B‐27 without vitamin A (1×; #12587010, Life Technologies), heparin (2 μg·mL^−1^; H3149‐100KU, Sigma‐Aldrich, St. Louis, MO, USA), EGF (10 ng·mL^−1^; AF‐100‐15‐1MG, Peprotech (Thermo Fisher Scientific), Rocky Hill, NJ, USA), FGF2 (10 ng·mL^−1^; #100‐18B‐500UG, Peprotech), ciprofloxacin (2 μg·mL^−1^ PHR1044‐1G, Sigma‐Aldrich), gentamycin (10 μg·mL^−1^; #11520506, Thermo Fisher), and fungin (2 μg·mL^−1^; Invivogen, San Diego, CA, USA). This medium is referred to as +GFs. The –GFs medium was identical except for the omission of heparin, EGF, and FGF2. Glioblastoma stem‐like cells were grown in suspension on a non‐adherent poly(2‐hydroxyethyl methacrylate)‐coated plates (poly‐HEMA; 192 066, Sigma‐Aldrich), whereas other lines were cultured on poly‐D‐lysine/laminin (poly‐D‐lysine (25 μg.mL^−1^, P7886, Sigma‐Aldrich)/laminin (2 μg/cm^2^, L2020, Sigma‐Aldrich)). Hypoxia experiments were performed using an incubator at 37 °C in a humid atmosphere with 5% O_2_ (HeraCell Vios 160i). Cultures were routinely tested for mycoplasma and authenticated by STR profiling. Cells were dissociated with Trypsin–EDTA (0,25%, #25200‐056, Thermo Fisher)/CaCl_2_ 20 mm (10035‐04‐8, Thermo Fisher)/DNase I (#10104159001, Roche Diagnostics, Mannheim, Germany) and used below passage 15. For endothelin‐response assays, cells were plated on Nunclon™ Delta plastic culture vessels, while flow cytometry and signaling assays were performed on cells growing on poly‐L‐ornithine (#P3655, 20 μg·mL^−1^; Sigma‐Aldrich). CHO control and EDNRA/EDNRB‐overexpressing lines were maintained using standard procedures for CHO cells.

### 2.5 RNA extraction, bulk RNA‐seq, and RT‐qPCR of patient tissues and cell cultures

Total RNA was extracted from cultured cells using the RNeasy kit (#74104, Qiagen, Hilden, Germany) for RNA‐seq, TRIzol (#15596018, Thermo Fisher), or the Arcturus PicoPure RNA kit (Thermo Fisher) for low cell numbers (e.g., FACS‐purified cells). Patient tissues were homogenized in cold TRIzol using an Ultra‐Turrax prior to extraction. RNA quantity was assessed with a NanoDrop spectrophotometer. For RT‐qPCR, cDNA was synthesized from 200 ng RNA (low‐grade cultures) or 500 ng RNA (glioblastoma/tissues) using random hexamers and reverse transcriptase, following the manufacturer's instructions. qPCR was performed with KAPA SYBR Fast (LC480 kit, #KK4610, Sigma‐Aldrich, St. Louis, MO, USA) on a LightCycler 480, and relative expression was calculated by the 2^−ΔΔ*C*t^ method using ACTB as the reference gene. Primer sequences are listed in Table S5. For bulk RNA‐seq, mRNA libraries were prepared and sequenced by BGI Genomics (Hong Kong, China, or Poland) on the DNBSEQ™ platform (paired‐end, 150 bp; ≥ 30 million reads/sample). Reads were quality‐filtered with SOAPnuke, aligned to the reference genome with HISAT2, and quantified using RSEM to obtain counts, FPKM, and TPM values. Differential expression was analyzed with DESeq2 (FDR ≤ 0.05). Gene expression (TPM) data and volcano plots were generated using the BGI Dr. Tom platform.

### 2.6 Protein extraction and Western blotting of patient tissues and cell cultures

Cells and patient tissues were lysed in RIPA buffer (Sigma‐Aldrich, #R0278) supplemented with protease (cOmplete™ Ultra Tablets, #05892970001, Roche) and phosphatase inhibitors (PhosSTOP™, #04906845001, Roche). For patient tissues, homogenization was performed in 2 mL tubes containing 1.4 mm zirconium–silicate beads (#116913100, Lysing Matrix D, MP Biomedicals, Irvine, CA, USA) using the manufacturer's protocol. Protein concentration was determined by BCA assay (#23225, #23227, Pierce™ BCA Protein Assay Kit, Thermo Fisher). For Western blotting, 10 μg of protein per sample was separated on 4–15% SDS–PAGE gels, transferred to nitrocellulose or PVDF membranes, blocked, and incubated overnight at 4 °C with primary antibodies (see Table S6). HRP‐conjugated secondary antibodies and ECL detection (Bio‐Rad Laboratories, Hercules, CA, USA) were used; β‐actin served as a loading control. Densitometry was performed using ImageLab v6.1 (Bio‐Rad).

### 2.7 Immunofluorescence on cell cultures and patient tissue sections

Immunofluorescences were performed on cells grown on glass coverslips and cultured in +GFs or –GFs medium for 5–7 days. For live staining, cells were incubated for 10 min at 4 °C in PBS containing 0.5% BSA and FcR blocking reagent followed by 15 min at room temperature with anti‐EDNRB (RB49, 2 μg·mL^−1^). After three PBS washes, cells were fixed with 4% paraformaldehyde for 15 min at RT, permeabilized/blocked in PBS containing 5% donkey serum and 0.1% Triton X‐100 for 30 min and labeled with Alexa Fluor secondary antibodies. For fixed‐cell staining, fixation was performed first, followed by blocking/permeabilization and overnight incubation at 4 °C with anti‐EDNRB and/or other primary antibodies (see Table S6). After PBS washes, Alexa Fluor secondary antibodies were applied for 1 h at RT. OCT‐embedded patient samples were cut into 10 μm cryosections (SnapFrost^®^‐frozen), fixed with 4% paraformaldehyde for 20 min at 4 °C, permeabilized/blocked (0.3% Triton X‐100, 5% donkey serum), and incubated overnight at 4 °C with primary antibodies. Secondary antibodies were applied for 1 h at RT. Isotype controls or omission of primary antibodies were used as negative controls. All samples were counterstained with Hoechst 33342, mounted in Fluoromount (F4680, Sigma‐Aldrich), and imaged using a Zeiss Apotome microscope (40–63×, Carl Zeiss Microscopy, Oberkochen, Germany).

### 2.8 Cell growth assay

Glioblastoma stem cells (20 000 per well) or diffuse low‐grade glioma cells (30 000 per well) were seeded in 48‐well plates with +GFs medium (400 μL per well). After adhesion, cells were treated every 2 days with endothelin dilution buffer (control) or recombinant ET‐1 or ET‐3 (4 nm, Sigma). The EDNRB agonist IRL1620 (10 nm, SCP0135, Sigma‐Aldrich) was applied following the same schedule. Dose–response assays (0.01–100 ng·mL^−1^; 4 pm–40 nm) were also performed for ET‐1 and ET‐3 (Fig. 3C). After 15–20 days, cells were dissociated with Trypsin–EDTA and counted using a Z2 Beckman Coulter counter (*n* = 12 technical replicates per condition).

### 2.9 EdU incorporation and cell death assays

LGG275 cells (3 × 10^4^/well) were seeded in 48‐well plates and treated every 2 days for 5–6 cycles with control buffer, ET‐1 (10 nm), ET‐3 (10 nm), or IRL1620 (10 nm). Proliferation was assessed using the Click‐It EdU kit (#BCK‐EdUFC647, BaseClick GmbH, Neuried, Germany) with 10 μm EdU (50 h), followed by fixation, permeabilization, Alexa Fluor 647–azide staining, and Hoechst 33342 counterstain. Cell death was evaluated using YO‐PRO‐1™/propidium iodide or Annexin V–AF647 labeling in appropriate buffers. Samples were analyzed on a MACSQuant cytometer (405, 488, 635 nm lasers) with isotype and fluorescence minus one controls.

### 2.10 Cell migration assay

LGG275 cells (3 × 10^4^ per well) were plated in laminin‐coated 24‐well plates (0.55 μg·cm^−2^, LN521, Biolamina AB, Sundbyberg, Sweden). After 24 h, medium was replaced with endothelin‐containing medium (10 nm), and time‐lapse imaging was performed using a Nikon A1R confocal microscope (Nikon Corporation, Tokyo, Japan) under controlled conditions (37 °C, 5% CO_2_, 19% O_2_, 95% Relative humidity) as described previously [57]. Images were acquired in mosaic mode (8 × 8, 20× objective, zoom 2) every 10 min for 4 or 24 h. Migration velocity and plot‐to‐origin graphs were generated from 100 and 50 tracked cells per condition, respectively, using MTrackJ (ImageJ) and DiPer software [58].

### 2.11 Gene set enrichment analysis (GSEA)

Transcriptomic profiles of glioma cell lines treated with endothelins were analyzed using GSEA included in the Dr. Tom platform (https://biosys.bgi.com) or GSEA software, [59, 60, 61] against the gene sets shown in the figures to identify enriched biological functions and pathways.

### 2.12 Flow cytometry

LGG275 cells (5 × 10^5^) were cultured on PDL/laminin in +GFs or –GFs medium for 5–7 days, then labeled either live or after 4% paraformaldehyde fixation with anti‐EDNRB (sheep) or RB49 (mouse) antibodies (2 μg·mL^−1^), followed by Alexa Fluor 647–conjugated secondary antibodies (1 : 2000). For CD44/OLIG1 labeling after endothelin treatment, LGG275 cells were plated on poly‐L‐ornithine–coated vessels in +GFs or –GFs medium and treated once daily for 5 days with either endothelin dilution buffer (control) or ET‐1 (10 nm). Fixed cells were permeabilized in PBS containing 1% BSA, 0.1% Triton X‐100, and 0.5 mm EDTA. Live cells were first labeled with PE‐conjugated anti‐CD44 (#130‐110‐394, Miltenyi Biotec, Bergisch Gladbach, Germany), then fixed, permeabilized and stained with anti‐OLIG1 (#AF2417, R&D Systems (Bio‐Techne), Minneapolis, MN, USA) in PBS containing 1% BSA, 0.1% Triton X‐100, and 0.5 mm EDTA. Nuclei were counterstained with Hoechst 33342. All samples were analyzed on a MACSQuant cytometer (405, 488, and 635 nm lasers; V1 450/50BP for Hoechst, R1 692/75 for Alexa Fluor 647) using isotype controls (rabbit IgG #3900, Cell Signaling Technology (Danvers, MA, USA); goat IgG #AB‐108‐C, R&D Systems (Bio‐Techne), Minneapolis, MN, USA; sheep IgG #5‐001‐A, R&D; mouse IgG #X093101‐2, Dako (Agilent Technologies), Santa Clara, CA, USA) and fluorescence minus one control.

### 2.13 Proteomic analyses

Proteins from LGG275 cells (1.5 × 10^6^) (+GFs) and treated once daily for 5 days with vehicle (PBS + 0.1% BSA) or ET‐1 (4 nm) were extracted in RIPA buffer (Sigma‐Aldrich, #R0278) with protease/phosphatase inhibitors 8 h after the last treatment. Proteins were digested with trypsin (S‐Trap, ProtiFi, Farmingdale, NY, USA) and labeled with a TMT10 kit (Thermo Fisher). Pooled samples were fractionated (8 fractions) by basic RP chromatography (Pierce™ High pH Reversed‐Phase Peptide Fractionation Kit) and analyzed by nanoLC–MS/MS (RSLC U3000 coupled to an Exploris480 with FAIMS). A gradient consisting of 0–31% B for 120 min, 31–50% B for 5 min (*A* = 0.1% formic acid, 2% acetonitrile in water; *B* = 0.1% formic acid in 80% acetonitrile) at 300 nL·min^−1^ was used to elute peptides from the capillary (0.075 mm × 500 mm) reverse‐phase column (Pepmap^®^, Thermo Fisher Scientific). Data were processed with MaxQuant v2.0.3.0 using the RefProteome_HUMAN‐cano_2023_02_UP000005640 and contaminant databases. Fixed modification: carbamidomethyl (C); variable: oxidation (M), acetyl (protein N‐term) and FDR ≤ 1% at protein/peptide level. Significance thresholds were determined using Perseus (version 1.6.15.0) based on the Significance B approach, with a *P*‐value cutoff set at 0.01 for protein ratios (Et/Ctrl). Only proteins found to be significantly regulated in at least 3 out of 5 replicates were retained for further analysis. The mass spectrometry proteomics data have been deposited to the ProteomeXchange Consortium via the PRIDE [62], partner repository with the dataset identifier PXD065902 and 10.6019/PXD065902.

### 2.14 IP₁ production and ERK1/2 phosphorylation assays (HTRF)

IP_1_ accumulation and ERK1/2 phosphorylation were quantified using the HTRF IP‐One Gq kit (#62IPAPE, Cisbio Bioassays, Codolet, France) and the HTRF Phospho‐ERK1/2 kit (#64ERKPEG, Cisbio Bioassays, Codolet, France), respectively, following the manufacturer's protocols [63, 64]. For IP_1_ assays, LGG275 cells were seeded in poly‐L‐ornithine–coated plates in +GFs or –GFs medium and cultured for 7 days. Cells were then treated for 5 min with vehicle (PBS + 0.1% BSA), ET‐1 or ET‐3 (40 nm), the EDNRB agonist IRL1620 (0‐1000 nm), or the EDNRB antagonist BQ‐788 (0‐100 000 nm) in the presence of EC_80_ ET‐1 (4 nm). Cells were lysed in the kit lysis buffer and incubated with d_2_‐coupled IP_1_ antibodies and terbium cryptate–coupled anti‐IP_1_. For pERK_1/2_ assays, cells were lysed in saturation buffer containing protease and phosphatase inhibitors, then incubated with Europium cryptate–coupled and d_2_‐coupled anti‐pERK_1/2_ antibodies. TR‐FRET signals (665 nm/620 nm ratio) were recorded using a PHERAstar plate reader. IP_1_ concentrations were calculated from the kit's standard curve, and pERK_1/2_ values were expressed relative to control conditions.

### 2.15 Intracellular calcium measurements

For population‐based measurements, LGG275 cells (1 × 10^5^) were seeded in Poly‐L‐ornithine–coated 96‐well plates (+GFs/–GFs, 7 days) and loaded with Cal520‐AM (1.25 μm, 1 h, 37 °C) in PBS containing 0.5 mm CaCl_2_ and 0.5 mm MgCl_2_. Fluorescence responses to ET‐1, ET‐3, IRL1620 agonist, BQ‐788 antagonist were recorded at 37 °C using an FDSS μCell plate reader (Hamamatsu Photonics, Hamamatsu, Japan; Ex 493 nm/Em 515 nm). ΔF (max–min) values were integrated and normalized to control with *FDSS Analyzer V3*. For single‐cell ratiometric imaging, LGG275 cells (5 × 10^4^) on poly‐D‐lysine/laminin‐coated glass coverslips were loaded with Fura‐2‐AM (6 μm, 30 min, 37 °C, 5% CO₂) and imaged in external buffer (130 mm NaCl, 5.4 mm KCl, 0.8 mm MgCl_2_, 10 mm HEPES, 5.6 mm glucose, 1.8 mm CaCl₂, pH 7.4). Excitation at 340 and 380 nm with emission at 505 nm was acquired every 2 s at 37 °C using a Lambda 421–equipped Olympus IX73 microscope (Olympus Corporation, Tokyo, Japan) with Zyla sCMOS camera and *Metafluor* software. The 340/380 nm fluorescence ratio was calculated over time from selected region of interest and normalized to baseline.

### 2.16 Measurement of potassium fluxes

Potassium channel activity was assessed in LGG275 cells using the FluxOR probe (#F20015, ThermoFisher) according to the manufacturer's protocol. After a 1‐min baseline recording, cells were stimulated with ET‐1 (40 nm), ET‐3 (40 nm), the EDNRB agonist IRL1620 (1 μm), or ET‐1/IRL1620 with apamin (1 μm), and fluorescence was recorded for 5 min on an FDSSμcell (Hamamatsu Photonics) at 37 °C. Data were analyzed with FDSS Analyzer V3 to quantify potassium fluxes.

### 2.17 Patch clamp

LGG275 cells (5 × 10^3^) were seeded in poly‐D‐Lysine/Laminin‐coated 35 mm‐diameter Petri dish 7 days before experimentation. For potassium channel identification, Petri dishes were also coated with sDLL4 to further promote a non‐proliferative state as previously shown [11]. The measurements were carried out at room temperature. Patch electrodes (3–5 m) were pulled from borosilicate glass capillaries (GC‐150 T, Harvard Aparatus, Holliston, MA, USA) using a vertical micropipette puller (Narishige, Tokyo, Japan). Voltage‐clamp experiments in whole‐cell configuration were performed using an Axopatch 200B amplifier with a CV 203BU headstage (Molecular Devices, San Jose, CA, USA). Voltage command pulses were generated by a personal computer equipped with an analog‐digital converter (Digidata 1550; Molecular Devices) using pCLAMP software v11.0 (Molecular Devices). Currents were filtered at 5 kHz and digitized at 10 kHz. The digitized currents were stored on a computer for later offline analysis. Intrapipette solution was composed of KCl 145 mm, MgCl2 1 mm, Mg‐ATP 1 mm, HEPES 10 mm, CaCl_2_ 8.7 mm, EGTA 10 mm, pH 7.2. The final pCa was 6 ([free Ca^2+^] = 1 μm) in experiments designed to detect the presence of SK channels by direct stimulation with intracellular solution. In experiments using an extracellular ligand (endothelin) to induce a physiological intracellular Ca^2+^ response a PCa 9 ([free Ca^2+^] = 1 nm) was used with the following intrapipette solution: KCl 145 mm, MgCl_2_ 1 mm, Mg‐ATP 1 mm, HEPES 10 mm, EGTA 0.5 mm, pH 7.2. The extracellular solution consisted of NaCl 140 mm, KCl 4 mm, MgCl_2_ 1 mm, CaCl_2_ 2 mm, glucose 11.1 mm, HEPES 10 m, pH 7.4. Macroscopic currents were measured in response to a ramp protocol, from ‐100 mV to +60 mV for 4 s from a resting potential of 0 mV. The interval between each ramp was 5 s. To monitor the effect of Apamin and ET1 on current amplitude, cells were held in the standard bath solution and perfused with this control solution to measure the control current. Then, the same solution containing either Apamin, ET1 or ET1 + Apamin was applied to the cell using a microperfusion system. Data were analyzed using a combination of pCLAMP software v11.0 (Molecular Devices), Microsoft Excel, and GraphPad Prism 8.0.

### 2.18 Effect of cytokines, YAP and Notch signaling on EDNRA and EDNRB expression

LGG275 cells were treated for five consecutive days with selected cytokines or pathway modulators. Treatments included ET‐1 (10 ng·mL^−1^, 4 nm), hOSM (10 ng·mL^−1^), hLIF (10 ng·mL^−1^), hBMP2 (10 ng·mL^−1^), hBMP4 (10 ng·mL^−1^), hBMP6 (10 ng·mL^−1^), hCNTF (10 ng·mL^−1^), IFNγ (10 ng·mL^−1^), and TDI‐011536 (5 μm) targeting the YAP1 pathway. Medium containing the respective compounds was renewed once daily. Cells were collected 8 h after the final treatment for RNA or protein extraction (See Tables S1, S4 and S5). For virus‐mediated pathway modulation, LGG275 or Gb7 cells were transduced with lentiviral or adenoviral vectors to overexpress constitutively active STAT3 (STAT3C; MOI = 50; gift from Linzhao Cheng, Addgene plasmid #24983; [65]), constitutively active YAP (YAP1‐S5A; MOI = 10; in‐house generated adenovirus; [66]), or activated Notch (NICD; [11]), together with the corresponding control vectors. After a 15‐day expansion period, transduced cells were sorted by FACS to isolate positive populations. Total RNA was extracted (Arcturus Picopure RNA kit) and processed for quantitative PCR analysis. Protein lysates were prepared in RIPA buffer supplemented with protease and phosphatase inhibitors for Western blotting.

### 2.19 Glioma cell–HUVEC coculture, RNA extraction, and bulk RNA sequencing

LGG275 (GFP‐labeled) and Gb7 (YFP‐labeled) glioma cells, as well as HUVEC endothelial cells (mCherry‐labeled), were generated by lentiviral transduction followed by FACS sorting. One day before coculture, 3.4 × 10^5^ HUVECs (Human Umbilical Vein Endothelial Cells; Promocell GmbH, Heidelberg, Germany) were seeded on gelatin‐coated plates in complete endothelial growth medium (#C‐22111, Promocell) supplemented with recombinant human EGF (5 ng·mL^−1^), basic FGF (10 ng·mL^−1^), IGF Long R3 (20 ng·mL^−1^), VEGF_165_ (0.5 ng·mL^−1^), ascorbic acid (1 μg·mL^−1^), heparin (22.5 μg·mL^−1^), hydrocortisone (0.2 μg·mL^−1^), and fetal calf serum (2% v/v). Glioma cells (3.0 × 10^5^) were then added and cocultured for 48 h. Following coculture, GFP^+^ or YFP^+^ glioma cells and mCherry^+^ HUVECs were isolated by FACS. Total RNA was immediately extracted using the Arcturus Picopure RNA kit (Thermo Fisher) for bulk RNA sequencing.

### 2.20 Statistical analysis

For all experiments, the statistical methods, number of replicates, and number of independent experiments are specified in the corresponding figure legends. Data analyses and graph generation were performed with GraphPad Prism software, version 10.0.0 (153) (GraphPad Software, Boston, MA, USA). All significance thresholds were defined as follows: ns (not significant), *P* ≥ 0.05; * (significant), 0.01 ≤ *P* < 0.05; ** (very significant), 0.001 ≤ *P* < 0.01; *** (highly significant), 0.0001 ≤ *P* < 0.001; **** (very highly significant), *P* < 0.0001. For RT‐qPCR, Bootstrap Ratios were calculated using BootstRatio method [67], same significance tests were used except that **** was used for very highly significant, *P* < 0.0005. Negative fold change values reflect the transformation –1/(linear fold change), which is used only for graphical symmetry when representing downregulation. R Statistical Software (version 4.5.2; R Core Team, 2025) and RStudio (version 4.5.2; Posit Team, 2025) were used for EDNRA methylation level assessment and data visualization was performed using the ggplot2 package [68].

### 2.21 Statement on language editing assistance

The writing of this manuscript was refined with the assistance of ChatGPT (OpenAI), which was used to improve grammar, clarity, and style without altering the scientific content.

## 3 Results

### 3.1 EDNRB is the main endothelin receptor expressed and is confined to astrocytic‐like tumor cell population in diffuse gliomas

**Fig. 1:**
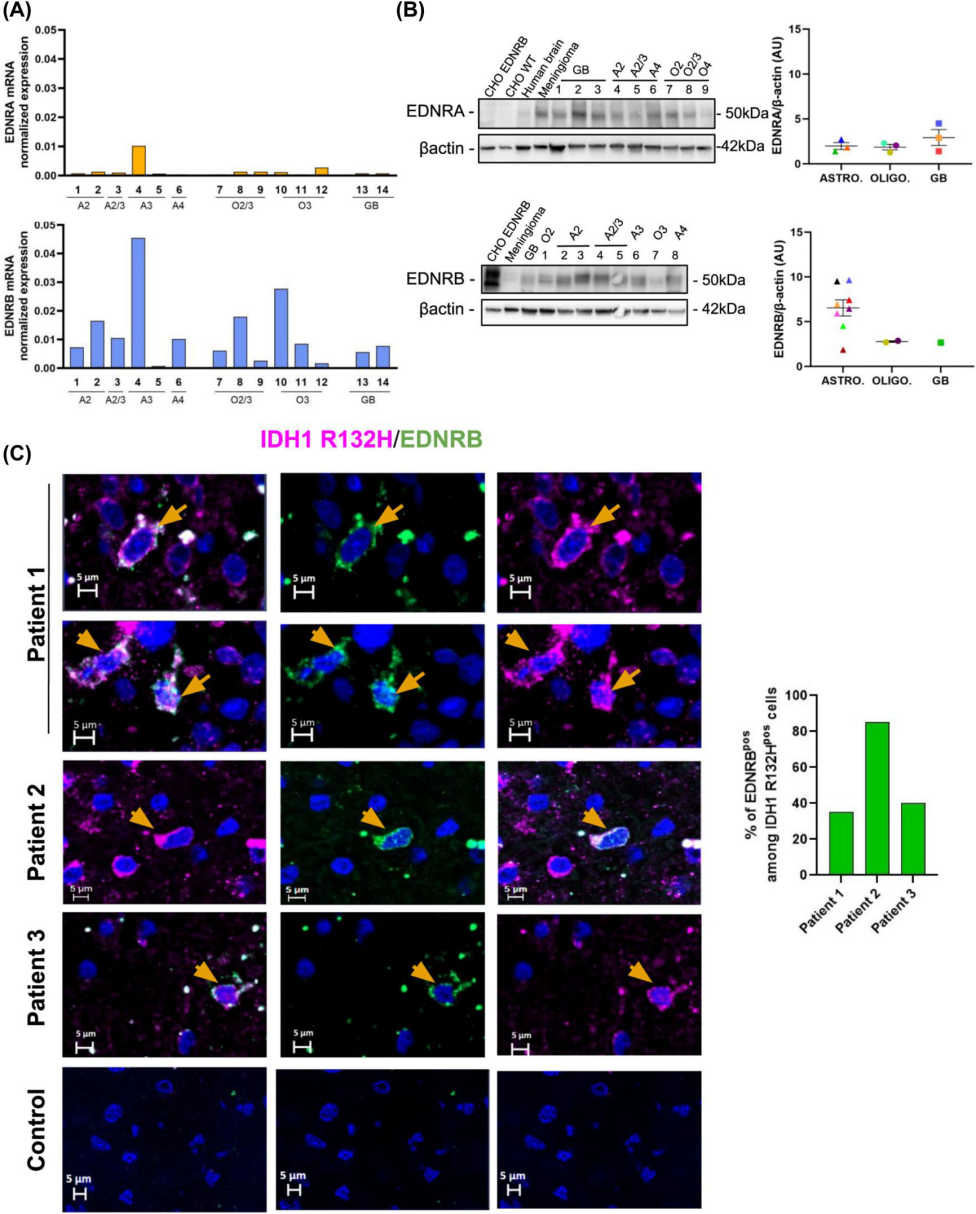
EDNRB is the predominant endothelin receptor expressed in gliomas. (A) RT‐qPCR quantification of *EDNRA* (*top*) and *EDNRB* (*bottom*) mRNA expression in glioma 14 patient samples normalized to *ACTB* gene (B) (*left*) WB of EDNRA (*top*) and EDNRB (*bottom*) protein expression in 9 glioma patient samples. CHO cells (CHO‐WT) were used as a negative control; meningioma served as a positive control for EDNRA. CHO cells (CHO Chinese Hamster Ovarian) overexpressing EDNRB (CHO‐EDNRB) are used as positive controls for EDNRB antibody and to test specificity of EDNRA antibody. β‐actin was used as loading control. (*right*): Quantification of EDNRA and EDNRB levels normalized to β‐actin (a.u., arbritary unit). Data are shown as mean±SEM. Tumor types: A = Astrocytomas, O = Oligodendrogliomas, GB = Glioblastomas. (C) Immunofluorescence co‐stainings of IDH1‐R132H and EDNRB in three IDH‐mutant diffuse low‐grade gliomas. Hoechst: nuclei. Orange arrows: double‐positive (IDH1‐R132H^+^/EDNRB^+^) tumor cells. Scale bars = 5 μm. EDNRA Endothelin Receptor Type A, EDNRB Endothelin Receptor Type B, ACTB Actin Beta, IDH1R132H, Isocitrate Dehydrogenase 1 (IDH1) gene, Arginine (R) at position 132 replaced by Histidine (H).

We initiated our analysis of *EDNRA* and *EDNRB* expression in diffuse gliomas by performing qPCR in fourteen primary diffuse glioma resections (Fig. 1A). *EDNRB* mRNA was detectable in 13/14 tumors (93%), with expression levels ranging from 0.0008 to 0.05 (relative expression with median ± SD = 0.08 ± 0.01). By contrast, *EDNRA* mRNA was detected at consistently lower levels, with only 1/14 tumor reaching 0.01 out of 14 (relative expression with median ± SD =0.0009 ± 0.003), and its expression was consistently lower than *EDNRB* (median *EDNRB/EDNRA* fold change of 9.3). *EDNRB* levels showed substantial inter‐patient variability (Coefficient of Variation CV = 99.8%), but were present across all histological subtypes (astrocytoma, oligodendroglioma, glioblastoma). To determine whether the mRNA differences translate at the protein level, we quantified EDNRA and EDNRB proteins in 9 additional samples that covered the main histological glioma subtypes and grades (Fig. 1B). Antibody specificity was verified in CHO cells engineered to overexpress either receptor (Fig. 1B). Although the small cohort size precluded robust statistics, both receptors were variably detected across tumors (CV_EDNRA_ = 45.1%; CV_EDNRB_ = 50.6%), and EDNRB generally surpassed EDNRA—especially in astrocytomas—mirroring the transcript data (EDNRA expression mean ± SD = 2.3 ± 1,0; EDNRB expression mean ± SD =5.5 ± 2,8).

Single‐cell transcriptomic datasets from gliomas dataset revealed that *EDNRB* is expressed not only in tumor cells but also in non‐neoplastic astrocytes (Fig. S1A) [69]. To unequivocally confirm EDNRB expression in tumor cells, we used the mutant IDH1 R132H protein as a reliable marker of tumor cells in IDH1‐mutant gliomas [70]. Employing double labeling of EDNRB and IDH1 R132H in 3 IDH1‐mutant patients, we observed that between 25% and 75% of IDH1 R132H^+^ tumor cells co‐express EDNRB (mean ± SD = 52% ± 24%) (Fig. 1C). Given the now well‐established intratumoral cellular heterogeneity of gliomas, we next investigated which tumor cell subtypes express *EDNRB* in glioblastomas, astrocytomas, and oligodendrogliomas using publicly available single‐cell RNA sequencing datasets. In both glioblastomas and oligodendrogliomas, *EDNRB* expression appears enriched in cells displaying an astrocyte‐like phenotype (Fig. S1B). In astrocytomas, correlations between *EDNRB* and astrocytic or oligodendrocytic markers confirm *EDNRB* predominance in tumor cells with an astrocytic signature (Fig. S1C). To validate at the protein level, the preferential expression of EDNRB in astrocyte‐like cells, we conducted co‐labeling experiments in one astrocytoma and one oligodendroglioma sample, using two markers of astrocytic and oligodendrocytic lineages, namely APOE and OLIG2, respectively. As shown in Fig. S1D,E, the vast majority of EDNRB^+^ cells co‐expressed APOE (mean ± SD = 94.1 ± 8.3%), whereas only a few co‐expressed OLIG2 (mean ± SD = 4.3 ± 6.1%), confirming the astrocytic identity of EDNRB‐expressing cells.

### 3.2 Expression of endothelin receptors in glioma cell lines

**Fig. 2:**
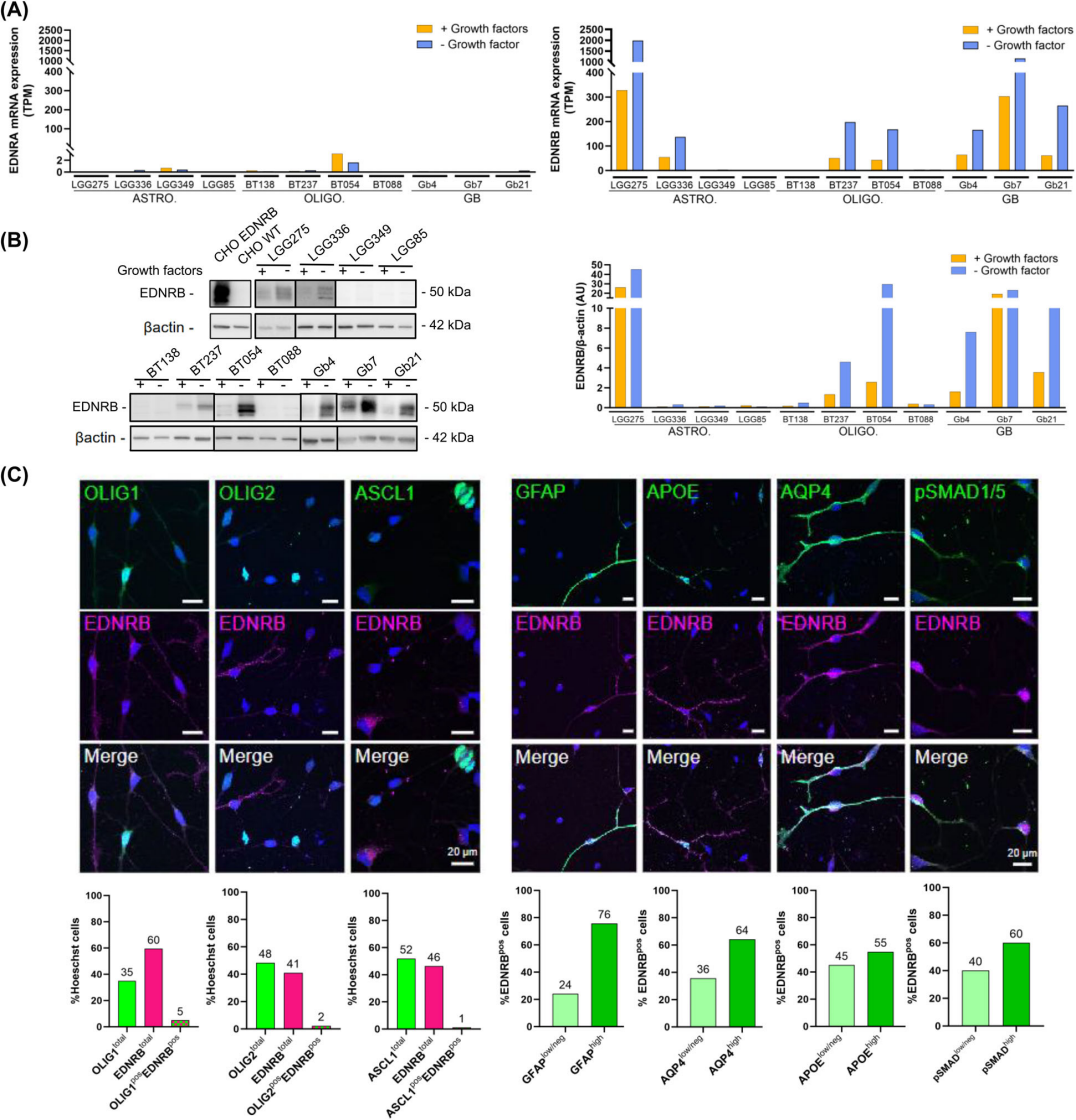
EDNRB dominates endothelin receptor expression in glioma cell lines. (A). RNA‐seq analysis of *EDNRA* *(left)* and *EDNRB (right)* (TPM) in 11 glioma cell lines cultured ±growth factors (GFs): Astrocytomas (LGG275, LGG336, LGG85, LGG349), oligodendrogliomas (BT138, BT237, BT054, BT088), and IDH–wild‐type glioblastomas (Gb4, Gb7, Gb21). (B). Western blot of EDNRB in the same lines ±GFs, with CHO‐WT (negative) and CHO‐EDNRB (positive) controls; β‐Actin: Loading control. *Right*: Densitometric quantification (a.u.). (C). Immunofluorescence of LGG275 cells showing that EDNRB^+^ cells lack oligodendroglial (OLIG1, OLIG2) and neural progenitor (ASCL1) markers but variably express astrocytic markers (GFAP, APOE, AQP4, pSMAD1/5). Nuclei: Hoechst. Scale bars = 20 μm. EDNRA Endothelin Receptor Type A, EDNRB Endothelin Receptor Type B, GF Growth Factors, OLIG1 Oligodendrocyte Lineage Transcription Factor 1, OLIG2 Oligodendrocyte Lineage Transcription Factor 2, ASCL1 Achaete‐Scute Family BHLH Transcription Factor 1 (MASH1), GFAP Glial Fibrillary Acidic Protein, APOE Apolipoprotein E, AQP4 Aquaporin 4, pSMAD1/5 Phosphorylated SMAD1/5.

To identify a suitable *in vitro* model to study endothelin signaling, we performed bulk RNA sequencing (RNA‐seq) on a panel of 11 patient‐derived glioma cell lines cultured in a serum‐free medium with growth factors (+GFs) (Fig. 2). This panel included three IDH1‐mutant lines (LGG275, LGG336, LGG85) derived from low‐ and high‐grade astrocytomas, and a high‐grade IDH1‐wt astrocytoma line (LGG349) [11, 49, 50, 51]. It also comprised four high‐grade oligodendroglioma lines (BT054, BT088, BT138, BT237) [52, 53] and previously characterized three glioblastoma cancer stem cell lines (Gb4, Gb7, Gb21) [55, 56]. To assess regulation by growth factors, we also repeated RNA‐seq after a 4‐day growth factor withdrawal, a condition promoting lineage commitment and proliferation reduction (‐GF). *EDNRB* was more strongly expressed than *EDNRA* in most cell lines (+GFs: mean_EDNRA_ ± SD = 0.4 ± 0,9 TPM; mean_EDNRB_ ± SD = 82.4 ± 118 TPM) but absent in four of them (LGG85, LGG349, BT138 and BT088). In the seven positive cell lines, *EDNRB* transcripts rose after growth factor (mean_EDNRB_ ± SD = 583.9 ± 719.7 TPM, mean fold increase = 4.5) whereas *EDNRA* remained undetectable or low (mean_EDNRA_ ± SD = 0.3 ± 0,5 TPM) (Fig. 2A); qPCR confirmed these results (Fig. S2A). Protein validation mirrored the RNA profile: WBs showed heterogeneous, but concordant EDNRB expression that increased after growth factor removal (mean fold increase of 2.2) (Fig. 2B). EDNRB migrated as two to three bands, suggesting alternative splicing and/or post‐translational modifications. Consistent with RNA, EDNRA protein was not detected in any of the four cell lines examined, in contrast to CHO cells engineered to overexpress EDNRA (Fig. S2B). Then, to assess surface expression of the EDNRB receptor, we performed flow cytometry and IF on non‐permeabilized live LGG275 cells using two independent antibodies targeting the extracellular domain of the receptor. Both approaches confirmed membrane localization of EDNRB and confirmed its upregulation following growth factor withdrawal (Fig. S2C,D).

As we found that EDNRB was mainly expressed by astrocytic‐like cells in patients (Fig. S1A–C), we tested whether this pattern was recapitulated *in vitro* using LGG275 cells [11] which showed higher *EDNRB* RNA expression than all other cell lines analyzed (Fig. 2A). After growth factor withdrawal, LGG275 cells display 2 distinct populations expressing either oligodendrocyte lineage transcription factors (OLIG1, OLIG2, ASCL1) or astrocyte‐lineage markers (GFAP, APOE, AQP4, pSMAD1/5) (Fig. 2C). Double immunostainings showed that EDNRB was largely absent from oligodendrocytic cells but co‐expressed with astrocytic markers, indicating its restriction to astrocytic‐like cells in LGG275 (Fig. 2C). scRNA‐seq analysis of the LGG275 line further confirmed that EDNRB^high^ cells correspond to cells with an astrocyte‐like transcriptional profile (Fig. S3A,B), consistent with what is observed in patient tumors (Fig. S1B,C,D). This analysis also revealed that *EDNRB*‐positive cells in LGG275 exhibit reduced expression of proliferation markers, including *PCNA* and *MKI67*, suggesting a lower proliferative state within this subpopulation (Fig. S3B) as observed in patients (Fig. S3C correlation matrix).

### 3.3 Endothelins reduce glioma cell proliferation and promote cell motility

**Fig. 3:**
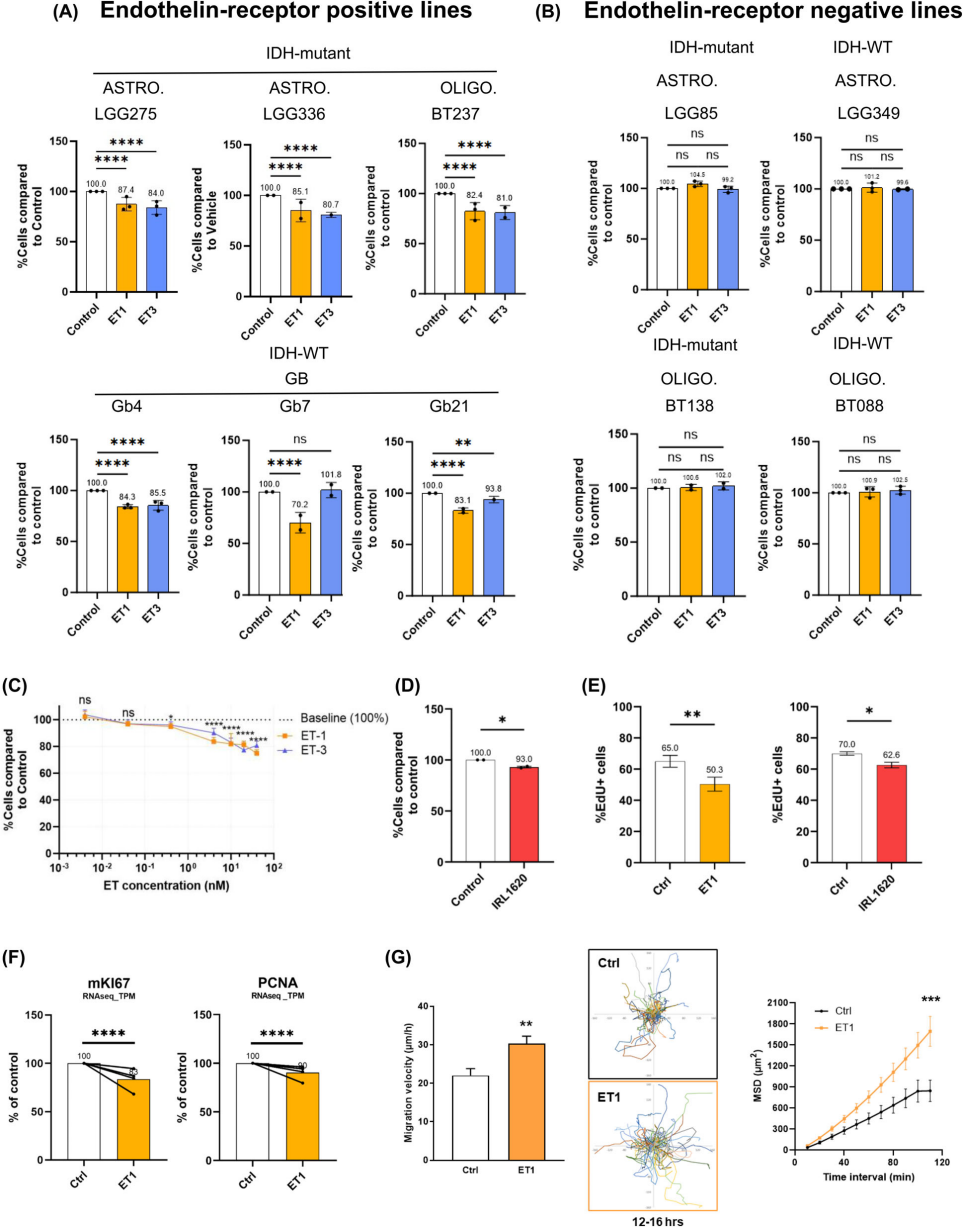
Endothelins reduce proliferation in a subset of diffuse glioma cell lines and promote migration. (A, B) Effect of ET‐1 and ET‐3 (4 nm) on glioma cell growth. (A) In endothelin‐receptor‐positive cell lines, both ligands significantly reduced cell numbers relative to vehicle (100%). (B) Endothelin‐receptor‐negative cell lines showed no significant change. Data are mean ± SD from three independent experiments (≥ 12 wells/condition); ANOVA with Dunnett's *post hoc* test. ****P < 0.0001; **P < 0.01; ns: not significant. (C) Dose–response in LGG275 cells showing significant growth inhibition by ET‐1 and ET‐3 from 0.4 nm. Mean ± SD; 12 wells at least per condition; ANOVA with Dunnett's *post hoc* test, ****P < 0.0001, *P < 0.05, ns = non‐significant. (D) EDNRB agonist IRL1620 (1 μm) reduces LGG275 growth (mean ± SD; 12 replicates; *n* = 2, unpaired *t*‐test, *P < 0.05). (E) Percentage of EdU^+^ proliferating LGG275 cells after ET‐1 (10 nm) or IRL1620 (1 μm) treatment. Data are shown as mean ± SD with *n* = 5 independent experiments (ET‐1) and *n* = 2 independent experiments with 6 replicates (IRL1620); paired *t*‐test. **P < 0.01; *P < 0.05. (F) Decreased *MKI67* (*left*) and *PCNA* (*right*) mRNA (TPM) in LGG275 after ET‐1 (10 nm). Data are normalized to control. Each dot represents an individual replicate. Paired measurements are connected by lines. Statistical significance was determined using Bootstratio test, *n* = 4 independent experiments. ****P < 0.0005. (G) Migration assays in LGG275 cells with or without ET‐1 (10 nm). (*Left*) Mean migration velocity over 12–16 h post‐treatment. Data are shown as mean ± SD with *n* = 100 cells per condition. ANOVA with Tukey's *post hoc* test. (*t*‐test, **P < 0.01). (*Middle*) Plot‐to‐origin trajectories showing more cells migrating farther. Each plot‐to‐origin displayed 50 color‐coded traces. (*Right*) Mean square displacement (MSD) over 10 min intervals from 12–16 h of 100 individual cells per condition shown as mean±SD, indicating greater area explored with ET‐1 (two‐way ANOVA, ***P < 0.001). EDNRB Endothelin Receptor Type B, ET‐1 Endothelin 1, ET‐3 Endothelin 3, EdU 5‐ethynyl‐2′‐deoxyuridine, PCNA Proliferating Cell Nuclear Antigen, MKI67 Marker of proliferation Ki‐67, TPM Transcripts per Million.

To evaluate how endothelin receptor stimulation influences cell growth, we treated ten glioma cell lines with either ET‐1 or ET‐3 and then quantified total cell numbers (Fig. 3A,B). Six of the treated lines exhibited a moderate yet statistically‐significant reduction in cell number (≈10–20% reduction depending on cell lines). The four lines that failed to respond were those lacking EDNRB expression (Fig. 2A,B) underscoring its role in the growth‐inhibitory effect. Focusing on the LGG275 line, dose–response assays with ET‐1/ET‐3 showed growth inhibition from 0.4 nm (Fig. 3C). The selective EDNRB agonist IRL‐1620 reproduced this effect, confirming EDNRB receptor involvement (cell number mean ± SD = 93.0% ± 1.0% of control; *P* = 0.013) (Fig. 3D). To assess whether the reduction was due to decreased proliferation or increased apoptosis, we performed EdU incorporation assays and apoptosis detection. Both ET‐1 and IRL‐1620 significantly reduced the proportion of EdU‐positive cells (ET1: 65% ± 8.5% to 50.3 ± 10.0%, *P* = 0.0014; IRL1620: 70.0% ± 2.9% to 62.6% ± 4.4%, *P* = 0.012) (Fig. 3E), whereas no significant change in apoptosis was observed (Fig. S4A). RNA‐seq of ET‐1‐treated cells (*see below*) further supported the antiproliferative effect of endothelin signaling showing a 10‐16% decrease in *MKI67* and *PCNA* transcript levels (mean_mKi67_ ± SEM = 83.5% ± 5.6% of control, *P* < 0.0005; mean_PCNA_ ± SEM = 90.2 ± 3.7% of control, *P* < 0.0005) (Fig. 3F).

Finally, as a reduction in proliferation can coincide with greater migratory capacity—a feature of the “go‐or‐grow” paradigm, in which diffuse glioma cells are either migratory, dispersing through surrounding tissue, or proliferative, driving tumor growth, with these states largely mutually exclusive [71]—we asked whether ET‐1 promotes cell motility. We therefore quantified the motility of LGG275 cells by time‐lapse microscopy, as previously described [57]. On a laminin‐coated substrate, LGG275 cells exhibited a mean migration velocity of 22.0 ± 1.8 μm/h. Addition of ET‐1 significantly increased migration speed (30.3 ± 1.9 μm/h, a mean fold increase of 1.38, *P* < 0.01) and territory explored, as shown by cumulative traces and mean square displacement (MSD) (Fig. 3G). We then assessed the onset of increased migration in endothelin‐treated LGG275 cells by measuring 4h intervals over a 2–22h period. Fig. S4B shows that migration significantly increased 10 h after ET‐1 addition (+139% in migration velocity) and remained elevated until 22 h (+173%). Similarly, ET‐1 enhanced the spatial exploration of the cells, as highlighted by the plot‐to‐origin graphs and MSD data (Fig. S4C,D). Altogether, our results demonstrate that ET‐1 stimulates the motility of LGG275 cells.

### 3.4 Endothelins promote a proneural‐to‐mesenchymal transition in glioma cells

**Fig. 4:**
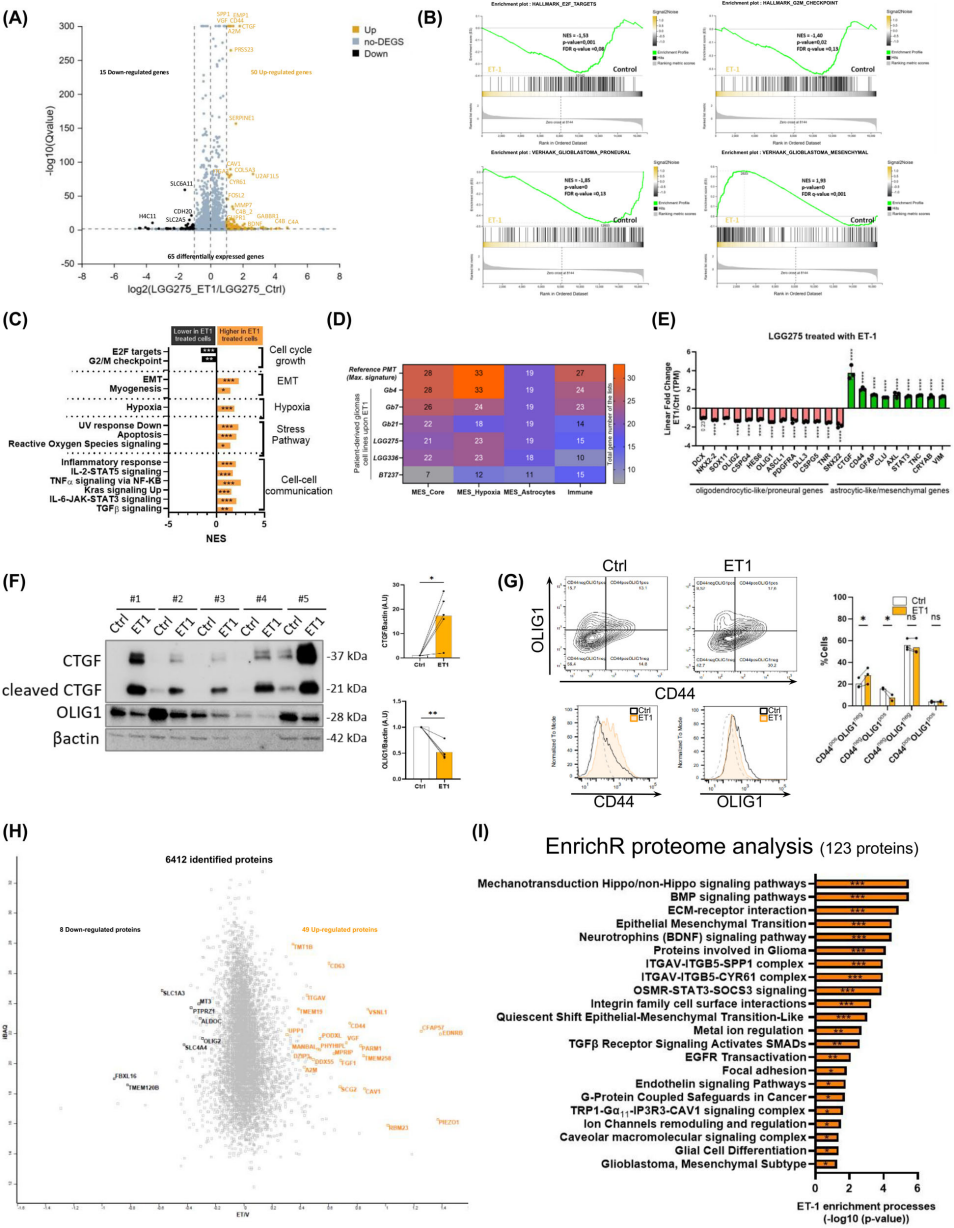
Endothelins promote a proneural‐to‐mesenchymal transition (PMT) in glioma cell lines. (A) Volcano plot of differentially expressed genes (DEGs) in LGG275 cells treated with ET‐1 (10 nm) versus vehicle, based on RNA‐seq (TPM). *X*‐axis: log_2_ fold change; *Y*‐axis: –log₁₀(FDR *q*‐value), n=3 independent experiments. (B) Gene Set Enrichment Analysis (GSEA) of ET‐1–treated LGG275 cells using MSigDB Hallmark and Curated C2 (CGP) collections. (*Top*) Downregulation of cell cycle–related gene sets (E2F targets, G2/M checkpoint). (Bottom) Decreased enrichment of Proneural and increased enrichment of Mesenchymal signatures (Verhaak_Glioblastoma signatures). NES, nominal *P*‐value, and FDR *q*‐value are shown. (C) Summary of significantly enriched GSEA Hallmark pathways (MSigDB) (FDR *q* < 0.25) in ET‐1–treated LGG275 cells. Pathways with positive enrichment scores are shown in orange, whereas pathways with negative enrichment scores are shown in black. ***P < 0.001; **P < 0.01; *P < 0.05 (nominal *P*‐value). (D) Heatmap showing overlap between ET‐1–upregulated genes in glioma cell lines and mesenchymal gene signatures from Chanoch‐Myers *et al*. [14] (MES_core, MES_Hypoxia, MES_Astrocyte, Immune). Top row: total genes per signature; rows below: number of overlapping genes in ET‐1–treated lines. Color intensity and values indicate absolute counts, with higher counts reflecting a stronger shift toward the corresponding mesenchymal subtype. (E) Fold change in lineage marker expression from RNA‐seq (TPM) in LGG275 cells treated with ET‐1, showing oligodendrocyte progenitor markers (purple) and astrocytic markers (green). Mean ± SD from TPM values (*n* = 3). Statistical significance was determined using DESeq2 with FDR‐adjusted *P*‐values (*q*‐values), ****P < 0.0001, *P < 0.05. Negative fold change values were obtained by applying the transformation –1/(linear fold change) to achieve graphical symmetry when representing downregulated genes. (F) (*Left*) Western blot of mesenchymal/astrocytic‐like marker CTGF (full‐length and cleaved) and proneural/oligodendrocytic‐like marker OLIG1 in LGG275 cells ±ET‐1. β‐actin: loading control. (*Righ*t) Quantification (A.U. arbritary unit), mean ± SD; *n* = 5; paired *t*‐test. *P < 0.05; **P < 0.01. (G) Flow cytometry of CD44 and OLIG1 in LGG275 cells ±ET‐1 (no GFs). *Top*: density plots; bottom: overlay histograms for CD44 and OLIG1. *Right*: quantification of CD44/OLIG1 subpopulations (mean ± SD; *n* = 3); two‐way ANOVA with Šídák's test. *P < 0.05; ns: not significant. (H) Scatter plot of proteomic changes in LGG275 cells ±ET‐1 by TMT‐ Mass spectrometry. *X*‐axis: ET‐1/vehicle ratio; *Y*‐axis: iBAQ intensity. Shown are proteins significant by Fisher's exact test (*P* < 0.05) and detected in ≥ 3/5 experiments with consistent direction. (I) EnrichR analysis of ET‐1–differentially expressed proteins, showing top enriched pathways ranked by –log₁₀(*P*‐value); Fisher's exact test (one‐sided hypergeometric over‐representation test) implemented in EnrichR. ***P < 0.001; **P < 0.01; *P < 0.05. TPM Transcripts per Million, ET‐1 Endothelin 1, NES Normalized Enrichment Score, FDR False Discovery Rate, OLIG1 Oligodendrocyte Lineage Transcription Factor 1, CTGF Connective Tissue Growth Factor (CCN2), TMT Tandem Mass Tag, IBAQ Intensity‐Based Absolute Quantification.

To investigate how endothelins affect glioma cells at the molecular level, we performed RNA seq on ET‐1‐treated LGG275 (*n* = 3; Fig. 4), LGG336, BT237 and Gb4, 7, 21 lines (*n* = 1 each, Fig. S5). In LGG275, the volcano plot shows 50 significantly upregulated and 15 downregulated genes (log_2_FC > |1|, *q* < 0.05) (Fig. 4A, Table S7). Gene Set Enrichment Analysis (GSEA) revealed a reduction in cell cycle–related programs, notably E2F targets and the G2/M checkpoint, along with enrichment of migration‐associated genes (Fig. 4B, Fig. S4E, Table S8), consistent with our proliferation and migration functional assays. Unexpectedly, GSEA using the Verhaak list of transcriptional signatures [72] that define the classical, proneural, mesenchymal, and neural subtypes of glioblastoma revealed that ET‐1 treatment promotes a proneural‐to‐mesenchymal transition (PMT) (Fig. 4B, Table S9). Notably, six well‐characterized mesenchymal‐associated genes were upregulated following ET‐1 treatment: *CCN1/CYR61* [73], *CCN2/CTGF*, *CD44*, *SERPINE1* [74, 75] and *CAV1* [75]. GSEA further showed that ET‐1 activates stress‐ and inflammation‐related transcriptional programs, with enrichment of gene sets linked to hypoxia, apoptosis, and reactive oxygen species (ROS), and downregulation of UV‐response genes (Fig. 4C, Table S8). The enrichment of the epithelial–mesenchymal transition (EMT) hallmark found by GSEA (Fig. 4C) likely reflects a PMT rather than a true EMT, given the non‐epithelial origin of glioma cells. In line with this transcriptional reprogramming, EnrichR enrichment analysis [76] demonstrated a robust activation of multiple convergent signaling pathways driving mesenchymal‐like transcriptional reprogramming, including TNF/NF‐κB, interferon–STAT3, GPCR–Gαq, TGFβ/BMP–SMAD, Hippo/YAP, MAPK, Notch, integrin, and calcium signaling, alongside potassium signaling (Fig. S5A).

This shift toward a more mesenchymal phenotype is not limited to LGG275. GSEA of RNA‐seq from LGG336 and the Gb4, Gb7, and Gb21 lines also revealed an EMT/PMT signature (Fig. S5B,C, Tables S10–S19). BT237 was the sole exception: although a trend toward ET‐1–induced EMT/PMT was observed in this oligodendroglioma line, the effect did not reach statistical significance (*P* = 0.10), suggesting that oligodendroglioma cells may be less susceptible to ET‐1–driven PMT. As observed in LGG275, ET‐1 also induced the expression of inflammation‐related genes and activated the IL6‐JAK‐STAT3 signaling pathway in most lines.

Although mesenchymal (MES) programs are recurrently reported in gliomas, their exact composition varies across studies. A recent meta‐analysis of ten published MES signatures distilled a 28‐gene “MES‐core” that appears in at least three independent datasets [14]. In our RNA‐seq dataset, ET‐1 treatment upregulated the vast majority of these core genes – between 22 and 28 genes – in five of the six lines examined (LGG275, LGG336, Gb4, Gb7, Gb21), whereas the oligodendroglioma line BT237 displayed only a marginal response (Fig. 4D, Table S20). The same study further resolved three recurrent MES sub‐states: i/MES‐Hyp, dominated by hypoxia/glycolysis genes; ii/ MES‐Ast, enriched for astrocytic markers and antigen‐presentation genes; iii/ an intermediate state that co‐expresses elements of both programs. Cross‐referencing these sub‐state signatures with our ET‐1 dataset revealed a strong concordance with the MES‐hypoxia module and, to a lesser extent, upregulation of selected MES‐astrocytic genes (*CLU, GFAP, GJA1, HOPX, SPARC*) (Fig. 4D, Table S20). Additionally, of the seven gene sets linked to the mesenchymal phenotype in this meta‐analysis, our data showed the strongest overlap with the immune/MHC cluster in the Gb4 and Gb7 cell lines (Fig. 4D), consistent with reports that this MES cluster distinguishes glioblastoma from lower‐grade gliomas [14]. Collectively, these results indicate that ET‐1 robustly drives a mesenchymal transition dominated by the MES‐Hyp type, while partially engaging astrocytic and immune‐associated pathways.

Given that acquisition of mesenchymal features is typically associated with repression of proneural/oligodendrocyte progenitor cell (OPC) markers, we next extracted from our ET‐1‐treated RNA seq dataset the expression of 13 canonical OPC genes across our six glioma cell lines. Indeed, ET‐1 treatment downregulated most OPC markers in five lines, with BT237 (oligodendroglioma) as the exception (Fig. 4E, Fig. S5B–C). In contrast, a curated panel of astrocytic and MES‐associated genes (*AXL, CD44, CLU, CRYAB, CTGF, GFAP, STAT3, TNC*, and *VIM*) was upregulated in the same five lines, again with minimal or no response in BT237 (Fig. 4E, Fig. S5B,C, Table S21). Protein‐level analyses corroborated these transcriptional changes. We first focused on OLIG1, a key OPC transcription factor, and *CCN2/CTGF* a prototypical mesenchymal matrix protein induced by TGF‐β, hypoxia, and inflammation [74]. LGG275 cells showed strong induction of both full‐length and cleaved CTGF isoforms (mean fold increase ±SEM: 17.3 ± 4.3, *P* = 0.019) accompanied by a decrease in OLIG1 levels in WB (mean fold decrease ±SEM: 1.9 ± 0.4, *P* = 0.002) (Fig. 4F). Flow cytometry further supported this phenotypic shift: the proportion of CD44^pos^/OLIG1^neg^ cells increased, while CD44^neg^/OLIG1^pos^ OPC‐like cells decreased following ET‐1 treatment (Fig. 4G), consistent with a proneural‐to‐mesenchymal transition. To assess global protein‐level changes, we performed quantitative mass spectrometry in LGG275 upon ET‐1 treatment (*n* = 3). ET‐1 significantly upregulated 49 proteins and downregulated 8 (ratio ET/ctrl significant in at least 3 out of 5 replicates) (Fig. 4H, Tables S22 and S23). Overall, Gene Ontology analysis reveals a decrease in replication‐associated proteins, consistent with the antiproliferative effect observed in LGG275 cells (Fig. S6A). Notably, two proteins characteristic of the proneural state (OLIG2 and PTPRZ1) were downregulated, whereas two canonical MES markers (CAV1 and CD44) were upregulated (Fig. 4H). Enrichment analysis of upregulated proteins using EnrichR and Gene Ontology revealed that proteins belonging to epithelial–mesenchymal transition, STAT3 signaling, and integrin–extracellular matrix interaction pathways are significantly over‐represented among the upregulated proteins, indicating activation of processes reminiscent of PMT (Fig. 4I, Tables S24 and S25). Multiple proteins belonging to the PMT gene set defined by Chanoch *et al*. [14] show increased expression upon ET‐1 treatment (Fig. S6B).

### 3.5 Endothelins stimulate Ca^2+^, ERK and STAT3 signaling in LGG275

**Fig. 5:**
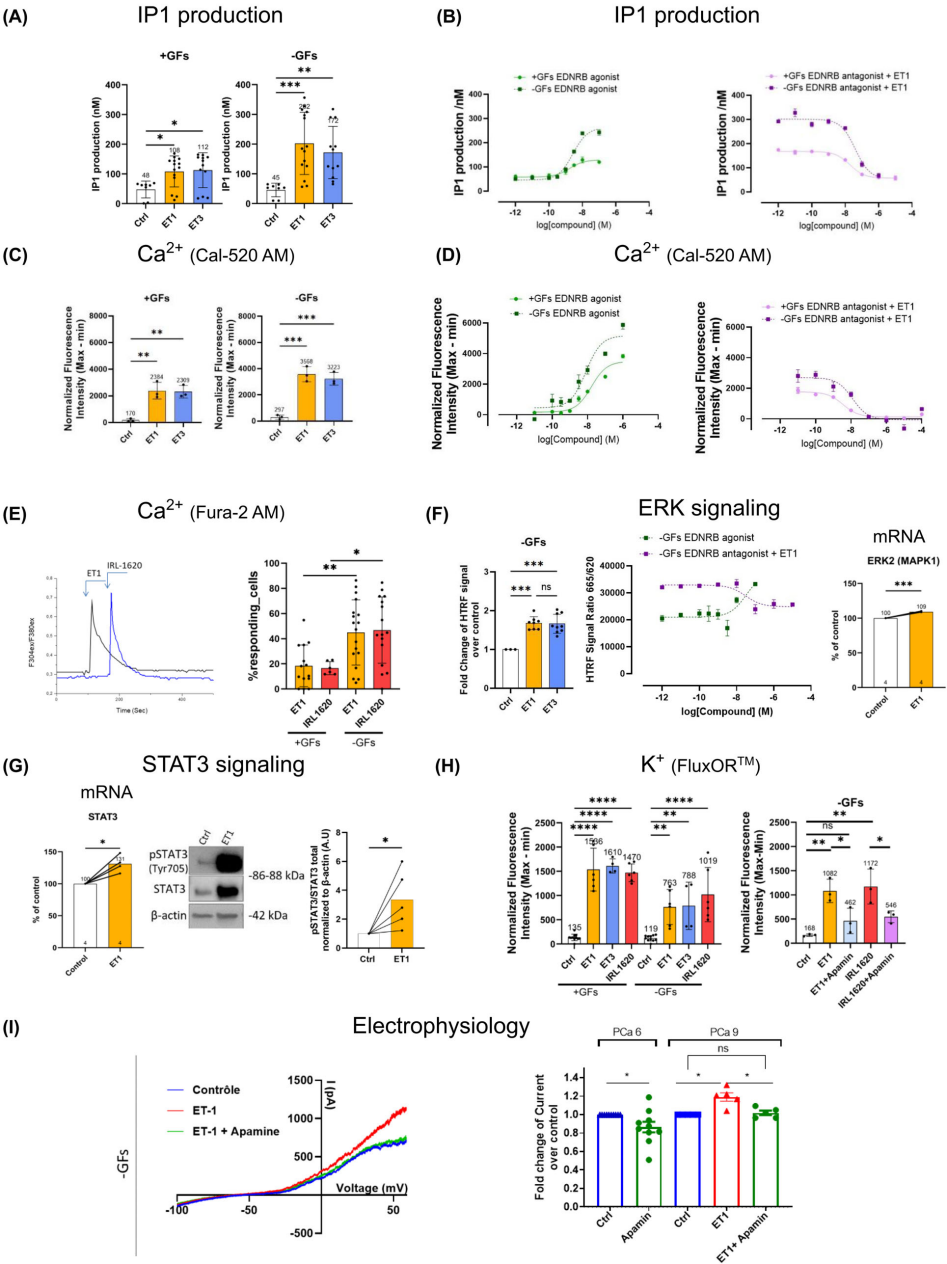
Endothelin signaling stimulates Ca^2+^, K^+^, ERK, and STAT3 pathways in LGG275 cells. (A) IP₁ production measured by HTRF in LGG275 cells cultured with (+GFs) or without (–GFs) growth factors after ET‐1 or ET‐3 treatment. Mean ± SD from *n* = 5 independent experiments; unpaired *t*‐test. ***P < 0.001; *P < 0.05. (B) IP_1_ production in response to the EDNRB agonist IRL‐1620 (*left*) or antagonist BQ‐788 plus EC_80_ ET‐1 (4 nm) (*right*) under ±GF conditions. Non‐linear regression dose–response curves from one representative of three experiments. (C) Intracellular Ca^2+^ flux (Cal‐520 AM) in control, ET‐1, or ET‐3 conditions under ±GFs. Mean ± SD; *n* = 3 independent experiments; ANOVA/Tukey. ***P < 0.001; **P < 0.01. (D) Ca^2+^ flux (Cal‐520 AM) in response to IRL‐1620 (*left*) or BQ‐788 plus ET‐1 (*right*) under ±GFs. Non‐linear regression curves from one representative of three experiments. (E) EDNRB‐dependent Ca^2+^ responses measured by Fura‐2. (*Left*) Representative traces for ET‐1 (40 nm) or IRL‐1620 (1 μm). (*Right*) Percentage of responding cells under ±GFs shown as mean±SD. *n* = 14 individual cells (ET‐1 + GFs), *n* = 6 individual cells (IRL1620 + GFs), *n* = 18 individual cells (ET‐1 ‐GFs), *n* = 16 individual cells (IRL1620 ‐GFs). Mann–Whitney test. **P < 0.01; *P < 0.05. (F) ERK Activation. (*Left*) Phospho‐ERK1/2 quantified by HTRF 5 min after ET‐1 or ET‐3 (40 nm) in –GF conditions (*n* = 3). (*Middle*) Phospho‐ ERK1/2 dose–responses to IRL‐1620 or BQ‐788 plus ET‐1 (4 nm) in –GF conditions. Mean ± SD; ANOVA/Tukey. ***P < 0.001; ns: not significant. Representative result from three independent experiments. (*Right*) *MAPK1* (ERK2) mRNA levels in ET‐1–treated versus control cells (RNA‐seq, TPM; n = 4 independent experiments). (G). STAT3 activation. (*Left*) *STAT3* mRNA (TPM) expression after ET‐1 treatment (*n* = 4 independent experiments). (*Middle*) WB of pSTAT3 (Tyr705), total STAT3, and β‐actin. (*Right*) Quantification of pSTAT3 normalized to total STAT3 and β‐actin (A.U.), relative to control, from n = 5 independent experiments. Paired *t*‐test. *P < 0.05. (H) K^+^ fluxes (FluxOR™ probe) under ±GFs in response to ET‐1, ET‐3, or IRL‐1620. (*Left*) Normalized fluorescence (Max–Min) from *n* = 3 independent experiments with 4 replicates (Ctrl) and duplicates (ET‐1, IRL1620); from *n* = 2 independent experiments with duplicates (ET‐3); ANOVA/Tukey tests, ****P < 0.0001; ***P < 0.001; **P < 0.01; ns: not significant. (*Right*) K^+^ fluxes under –GFs induced by ET‐1 or IRL‐1620 and blocked by apamin (1 μm) a blocker of SK channels. Normalized fluorescence (Max–Min) are plotted as Mean ± SD from *n* = 3 independent experiments. Multiple unpaired *t*‐tests. ****P < 0.0001; ***P < 0.001; **P < 0.01; *P < 0.05; ns: not significant. (I) Whole‐cell voltage‐clamp recordings under –GFs. (*Left*) I–V curves showing ET‐1–induced outward K^+^ currents (red) blocked by apamin (green) versus control (blue). (*Right*) Fold change in current amplitude under high (PCa6, 1 μm) or physiological (PCa9, 1 nm) intracellular Ca^2+^. At PCa6, currents were reduced by apamin; at PCa9, ET‐1 increased current amplitude, partially blocked by apamin. Mean ± SD; *n* = 6–7 cells; unpaired *t*‐test. *P < 0.05; ns: not significant. IP1 Inositol monophosphate, HTRF Homogeneous Time‐Resolved Fluorescence, ERK1/2 Extracellular signal‐Regulated Kinase 1/2, MAPK Mitogen‐Activated Protein Kinase, TPM Transcripts per Million, STAT3 Signal Transducer and Activator of Transcription 3, SK Potassium Calcium‐Activated Channel Subfamily N Member 3/Small Conductance.

Mesenchymal transition in gliomas is largely driven by STAT3 signaling, with ERK and calcium pathways also implicated [77, 78, 79]. Our RNA‐seq and proteomic data (Fig. 4, Figs S5 and S6) suggested activation of these pathways, leading us to test whether endothelins stimulate Ca^2+^, ERK, and STAT3 signaling in LGG275 cells. We first examined calcium signaling. As EDNRB canonically couples to Gαq/11–PLC–IP3, we measured IP1 accumulation, a downstream metabolite of IP3, using the FRET‐based HTRF assay. ET‐1 or ET‐3 significantly increased IP1, with stronger effects under growth factor–deprived conditions (Fig. 5A), consistent with the previously noted EDNRB upregulation (Fig. 2A,B). The EDNRB agonist IRL‐1620 reproduced this effect, which was abolished by the competitive antagonist BQ788, confirming specificity (Fig. 5B). To directly assess Ca^2+^ mobilization, we used Cal‐520‐AM (Fig. 5C,D) and Fura‐2‐AM (Fig. 5E), both showing robust ET‐1–evoked Ca^2+^ increase (Cal520‐AM: 12.1 fold increase; Fura‐2 AM: ΔF/F_0_ increase of 140%). These effects were sensitive to the EDNRB agonist and antagonist and were further amplified upon growth factor withdrawal (associated with a rightward shift, corresponding to ~0.6‐log increase in agonist EC₅₀ and an ~1‐log increase in antagonist EC_50_) (Fig. 5D), consistent with the increased expression of EDNRB under these conditions. Similar EDNRB‐dependent Ca^2+^ responses to ET‐1/ET‐3 were observed in BT237 cells (Fig. S7A).

To assess MAPK/ERK activation, we quantified ERK1/2 phosphorylation (Thr202/Tyr204) by HTRF in the LGG275 line. As ERK1/2 phosphorylation was already near maximal in growth factor–rich medium preventing any further increase upon endothelin stimulation (Fig. S7B), we next examined ERK1/2 activation in the absence of growth factors. Under growth factor–deprived conditions, ET‐1 and ET‐3 markedly increased pERK expression (mean fold increase±SD = 2.2 ± 0.25, ET‐1 *P* = 0.019, ET‐3 *P* = 0.04), an effect reproduced by IRL‐1620 and reduced by BQ788, confirming EDNRB dependence (Fig. 5F). Additionally, ET‐1 upregulated MAPK1 (ERK2) mRNA (RNA seq), corroborating pathway activation (*P* = 0.0001) (Fig. 5F).

STAT3 signaling was likewise induced: RNA‐seq showed upregulation of STAT3 mRNA, and WB confirmed a significant increase in pSTAT3/STAT3 upon endothelin treatment in LGG275 cells (mean fold increase ±SEM = 2.36 ± 0.9, *P* = 0.03) (Fig. 5G) and in other cell lines (Fig. S7C).

### 3.6 Activation of an apamin‐sensitive Ca^2+^‐regulated K^+^ current by endothelins in LGG275 cells

We previously showed that LGG275 cells express functional apamin‐sensitive K^+^ channels (*KCNN3/SK3*), which are activated by intracellular Ca^2+^ [11], RNA seq also revealed the expression of another calcium‐activated K^+^ channel, namely *KCNN2/SK2* in these cells (Fig. S7D). Both channels are detected at the protein level with variation upon growth factor removal (Fig. S7D,E). Given that endothelins strongly elevate intracellular Ca^2+^ and that *EDNRB* expression correlates with *SK2/3* in glioma patient datasets (Fig. S7F), we hypothesized that SK channels are activated downstream of endothelin signaling. To test this, we used the FluxOR probe, which enables real‐time monitoring of K^+^ channel activity in living cells [80]. Its functionality was validated with the K^+^ ionophore nigericin, which induced a clear fluorescence response (Fig. S7G). A Ca^2+^ ionophore (ionomycin) further confirmed that Ca^2+^ influx activates K^+^ channels in LGG275 cells, with partial inhibition by apamin under growth factor–free conditions (Fig. S7G). Using this probe, we found that ET‐1, ET‐3, and IRL‐1620 all triggered measurable K^+^ fluxes in both growth factor–rich and –deprived media, a response reduced by apamin, indicating mediation, at least in part, by apamin‐sensitive K^+^ channels (Fig. 5H). To further confirm functional SK channel activation, we performed whole‐cell electrophysiology. Under high intracellular Ca^2+^ (PCa6, 1 μm), cells displayed spontaneous SK‐type currents partially blocked by apamin (Fig. 5I, Fig. S7H). At lower, physiological Ca^2+^ (PCa9, 1 nm), ET‐1 application evoked an apamin‐sensitive current, demonstrating that ET‐1 activates SK channels (Fig. 5I).

### 3.7 BMPs, IL‐6–related cytokines and endothelins differentially control EDNRB expression

**Fig. 6:**
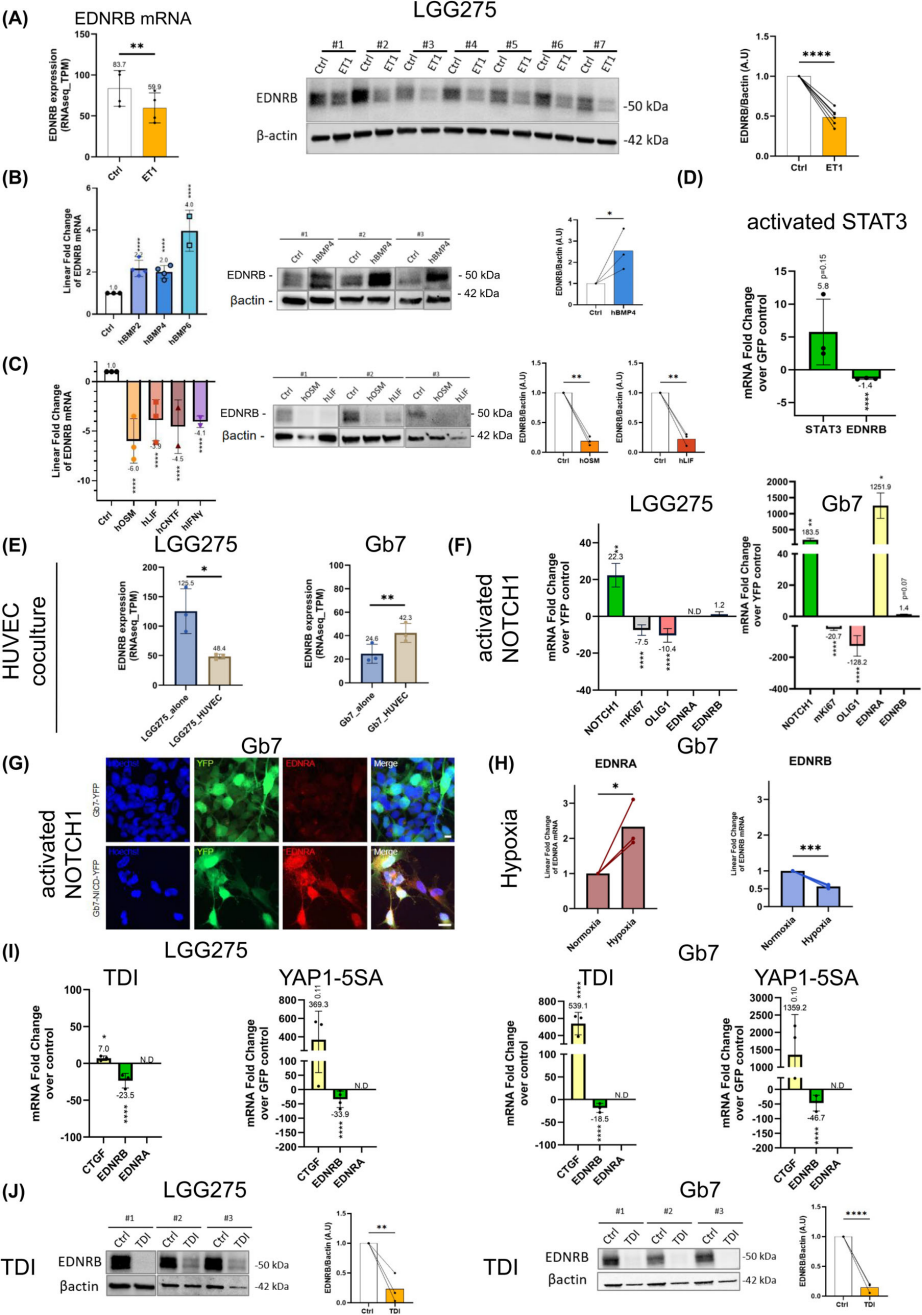
Dynamic regulation of endothelin receptor expression in diffuse glioma cells. (A) ET‐1 reduces *EDNRB* in LGG275 cells. (*Left*) mRNA (RNA‐seq, TPM; *n* = 4 independent experiments). Data are shown as mean ± SD and unpaired t‐test was used, **P < 0.01 (*Middle*) Representative Western Blot (WB) (*n* = 7 independent experiments). (*Right*) Quantification is shown as values normalized to the control (A.U. arbitrary units, β‐Actin–normalized). Each dot represents an individual replicate. Paired measurements are connected by lines. Statistical significance was determined using paired *t*‐test, ****P < 0.0001. (B) BMPs (10 ng·mL^−1^) increase *EDNRB* expression. (*Left*) mRNA fold change values were calculated using the ΔΔCt method and are presented as mean ± SD (RT‐qPCR, *n* = 3 independent experiments). Statistical significance was determined using Bootstratio test, ****P < 0.0005. (*Middle*) WB for hBMP4 (*n* = 3 independent experiments). (*Right*) Quantification is shown as values normalized to the control (arbitrary units, β‐Actin–normalized). Each dot represents an individual replicate. Paired measurements are connected by lines. Statistical significance was determined using paired *t*‐test (WB); *P < 0.05. (C). IL‐6 family cytokines (10 ng·mL^−1^) (hOSM, hLIF, hCNTF) and IFN‐γ reduce *EDNRB* in LGG275. (*Left*) mRNA fold change values were calculated using the ΔΔCt method and are presented as mean ± SD (RT‐qPCR, *n* = 3 independent experiments). Negative fold change values were obtained by applying the transformation –1/(linear fold change) to achieve graphical symmetry when representing downregulated genes. Statistical significance was determined using Bootstratio test, ****P < 0.0005. (*Middle*) WB for hOSM/hLIF (*n* = 3 independent experiments). (*Right*) Quantification is shown as values normalized to the control (arbitrary units, β‐Actin–normalized). Each dot represents an individual replicate. Paired measurements are connected by lines. Statistical significance was determined using paired *t*‐test, **P < 0.01. (D) Constitutively active STAT3 decreases *EDNRB* mRNA. Fold change values were calculated using the ΔΔ*C*_t_ method and are presented as mean ± SD (RT‐qPCR, *n* = 3 independent experiments). Negative fold change values were obtained by applying the transformation –1/(linear fold change) to achieve graphical symmetry when representing downregulated genes. Statistical significance was determined using Bootstratio; ****P < 0.0005. (E) HUVEC endothelial cells coculture reduces *EDNRB* in LGG275 and Gb7 cells (RNA‐seq, TPM; *n* = 3 independent experiments). Data are shown as Mean ± SD and unpaired *t*‐test was used: *P < 0.05 (LGG275), **P < 0.01 (Gb7). (F) Notch activation (NICD‐YFP) decreases MKI67 and OLIG1, increases EDNRA in Gb7 but not LGG275, and leaves EDNRB unchanged. mRNA fold change values were calculated using the ΔΔ*C*_t_ method and are presented as mean ± SD (RT‐qPCR, *n* = 3 independent experiments). Negative fold change values were obtained by applying the transformation –1/(linear fold change) to achieve graphical symmetry when representing downregulated genes. Statistical significance was determined using Bootstratio test: ****P < 0.0005, **P < 0.01, *P < 0.05. (G) Immunofluorescence: NICD (NICD‐YFP virus) increases EDNRA^+^ cells in Gb7 cultures. Scale bar: 10 μm. (H) Hypoxia (5% O_2_, 5 days) increases *EDNRA* and decreases *EDNRB* in Gb7. mRNA fold change values were calculated using the ΔΔCt method and are presented as mean ± SD (RT‐qPCR, *n* = 3 independent experiments). Each dot represents an individual replicate. Paired measurements are connected by lines. Statistical significance was determined using paired *t*‐test, *P < 0.05, ***P < 0.001. (I) Hippo/YAP1 activation by TDI (5 μm) or YAP1‐5SA increases *CTGF* and decreases *EDNRB* in LGG275 and Gb7 (RT‐qPCR; *n* = 3 independent experiments). *EDNRA* was not detected (N.D). Fold change values were calculated using the ΔΔ*C*_t_ method and are presented as mean ± SD (RT‐qPCR, *n* = 3 independent experiments). Negative fold change values were obtained by applying the transformation –1/(linear fold change) to achieve graphical symmetry when representing downregulated genes. Statistical significance was determined using Bootstratio, *P < 0.05; ****P < 0.001. (J) TDI reduces EDNRB protein in LGG275 and Gb7 (WB; *n* = 3 independent experiments). Quantification is shown as values normalized to the control (arbitrary units, β‐Actin–normalized). Each dot represents an individual replicate. Paired measurements are connected by lines. Statistical significance was determined using paired *t*‐test; **P < 0.01, ****P < 0001. TPM Transcripts per Million, BMP Bone Morphogenetic Protein, OSM Oncostatin M, LIF Leukemia Inhibitory Factor, CNTF Ciliary Neurotrophic Factor, STAT3 Signal Transducer and Activator of Transcription 3, HUVEC Human Umbilical Vein Endothelial Cells, NICD Notch Intracellular Domain, YAP Yes‐Associated Protein, CTGF Connective Tissue Growth Factor (CCN2).

In light of EDNRB's functional impact on glioma cells, we then investigated the regulation of its expression at both RNA and protein levels. We first observed that treatment of LGG275 cells with ET‐1 led to a reduction in both *EDNRB* mRNA and protein levels (RNA fold decrease mean ± SEM = 1.4 ± 0.3, *P* < 0.01 and protein fold decrease mean ± SEM = 1.9 ± 0.15, *P* < 0.0001) (Fig. 6A). Since EDNRB is enriched in astrocyte‐like tumor cells, we next tested whether microenvironmental cues promoting astrocytic differentiation affect EDNRB levels. We first treated LGG275 cells with BMP2, BMP4, or BMP6—cytokines known to promote astrocytic differentiation and quiescence [81, 82]. All three BMPs significantly increased *EDNRB* mRNA (mean fold increase ±SEM = 2.7 ± 0.3, *P* < 0.0005) and BMP4 also elevated EDNRB protein (mean fold increase ±SEM = 2.6 ± 0.6, *P* < 0.049) (Fig. 6B). We next tested other pro‐inflammatory cytokines that promote astrocytic fate—LIF, OSM, CNTF, and IFN‐γ. In sharp contrast to BMPs, each of these cytokines markedly repressed *EDNRB* transcription (mean fold decrease ±SEM = 3.9 ± 0.6, *P* < 0.0005), and EDNRB protein became almost undetectable after LIF or OSM treatment (mean fold decrease 4.8 ± 1.3, LIF *P* = 0.0058; OSM *P* = 0.027) (Fig. 6C). Because LIF and OSM activate STAT3 transcription factor [83], we transduced LGG275 cells with a constitutively active STAT3 mutant; this alone significantly reduced *EDNRB* mRNA (mean fold decrease ±SEM = 1.36 ± 0.1 *P* < 0.0005), implicating STAT3 in the repression (Fig. 6D).

### 3.8 Endothelin receptors expression is controlled by endothelial cells, hypoxia, Notch and hippo/YAP1 pathways

Blood vessels greatly influence glioma cells by providing ligands that activate Notch1 and Hippo/YAP1 signaling, pathways critical for stem and cancer stem cell maintenance [20, 21, 56, 84,85]. However, the roles of endothelial cells, Notch1, and Hippo/YAP1 in regulating endothelin receptors remain poorly defined. To address this, as performed previously [55], we cocultured mCherry^+^ endothelial cells (HUVEC) and IDH1‐mutant LGG275 or IDH1‐wt GB Gb7 GFP^+^ cells and then sort GFP^+^ cells and performed RNA‐seq (*n* = 3) to assess EDNRA and EDNRB expression. Surprisingly, LGG275 and Gb7 react differentially by downregulating and upregulating EDNRB, respectively (Fig. 6E), while EDNRA expression remains extremely low compared with EDNRB (TPM <0.07 for EDNRA vs TPM > 24 for EDNRB in LGG275 and Gb7) (*data not shown*). To directly test the effect of Notch1 activation, we transduced LGG275 and Gb7 cells with a constitutively active form (NICD) (Fig. 6F). As reported previously [11, 56], NICD decreased proliferation—measured via *MKI67*—and reduced expression of the proneural transcription factor *OLIG1* transcripts in both lines (Fig. 6F). While *EDNRB* was unchanged, *EDNRA* was strongly induced (mean fold increase ±SEM = 1251.8 ± 396, *P* < 0.05) in Gb7 but remained undetectable in LGG275 (Fig. 6F). This strong induction in Gb7 was further confirmed by IF (Fig. 6G). Given that hypoxia has been shown to work in a Notch‐dependent manner and increases the expression of direct Notch target genes [86, 87], we also assessed the impact of hypoxia on endothelin receptor expression in Gb7 cells. As shown in Fig. 6H, exposure to reduced oxygen levels (5% O_2_) led to a moderate but significant increase in *EDNRA* expression (mean fold increase ±SEM = 1.3 ± 0.4, *P* = 0.027), accompanied by a concomitant decrease in *EDNRB* (mean fold decrease ±SEM = 2.3 ± 0.2, *P* = 0.0001). Finally, we explored the impact of Hippo/YAP1 pathway activation in LGG275 and Gb7 both pharmacologically using TDI‐011536, an inhibitor of LATS1/2 kinase for YAP1 [88] or by transducing cells with an active form of YAP1 (5SA) [66]. Both treatments robustly upregulated CTGF RNA and protein, confirming pathway activation (Fig. 6I, Fig. S8A,B). Under these conditions, EDNRA remained undetectable, whereas EDNRB was strongly repressed at RNA and protein levels (LGG275‐Gb7 *EDNRB* mRNA mean fold decrease ±SEM 30.6 ± 5.6, *P* < 0.0005; LGG275‐Gb7 EDNRB protein mean fold decrease ±SEM = 5.26 ± 2.5, LGG275 *P* = 0.0053, Gb7 *P* < 0.0001) (Fig. 6I,J).

### 3.9 Bioinformatic and spatial transcriptomics reveal a vascular‐associated subpopulation of EDNRA ^+^ tumor cells in GB

Because Notch signaling and hypoxia both upregulate EDNRA *in vitro* in the Gb7 line (Fig. 6F,G), we next investigated whether EDNRA^+^ tumor cell subpopulations exist in gliomas. Previous studies reported higher *EDNRA* expression in high‐grade compared with low‐grade gliomas, notably in vessels [89, 90]. We confirmed this trend by IHC on a glioma tissue array [91, 92] (Fig. S9A) and by IF on one grade II and one GB gliomas (Fig. S9B). In GB, EDNRA staining was stronger and enriched in vascular structures (Fig. S9B), co‐localizing with CD31, consistent with its expression by mural cells such as pericytes and smooth muscle cells [23, 93]. This vascular enrichment is a hallmark of GB compared to low‐grade glioma and contributes to tumor progression by enhancing perfusion, sustaining metabolic demands, and facilitating invasive behavior [23, 94, 95]. scRNA‐seq analyses of GB further confirmed robust EDNRA expression in vascular cells (Fig. S1A) [69]. Unexpectedly, a small subset of tumor cells also expressed EDNRA in this dataset (Fig. S1A, red arrow), a finding independently reproduced in another GB dataset (Fig. S3C, red arrow) [13]. Given previous reports that some vascular‐like cells in GB may derive from tumor cells [96], we hypothesized that these EDNRA^+^ tumor cells may represent a vascular‐like tumor subpopulation supporting the increased vascularization found in aggressive gliomas. This hypothesis was further supported by our earlier finding that Notch activation drives Gb7 cells toward a vascular‐like phenotype and perivascular localization [56]. To support this possibility in patient tumors, we analyzed two other independent GB scRNA‐seq datasets: (1) The study by Xi *et al*. shows that some tumor cells co‐purifying with vascular cells express high *EDNRA* and low *EDNRB* (Fig. S9C) [97]; (2) Wang *et al*. [98] identified a tumor subpopulation (Cluster 7) characterized by high *EDNRA* expression, enrichment for angiogenesis and EMT genes, and association with recurrent GB, which they proposed to be linked to blood vessels. To further assess this latter possibility, we mapped cells with this Cluster 7 transcriptomic profile using spatial transcriptomic data from three GB patients from Ravi *et al*. [99]. Although *EDNRB* expression was generally higher than *EDNRA* across tumor sections (Fig. S10A), spatial mapping of cells with Cluster 7 gene signature revealed that EDNRA^+^ tumor cells co‐localized with endothelial and pericyte clusters, primarily in perivascular regions (Fig. S10B, red arrows). Collectively, these scRNA‐seq and spatial transcriptomic data establish the existence of a minor but distinct population of EDNRA^+^ tumor cells associated with the vasculature in GB.

## 4 Discussion

In the healthy brain, endothelin signaling through EDNRA and EDNRB shows distinct patterns of expression and function, with EDNRA predominantly associated with vascular cells and EDNRB with glial populations [26, 31]. In the vascular compartment, endothelins classically promote proliferation and hypertrophic growth of vascular smooth muscle cells through EDNRA‐dependent activation of Gαq/PLCβ, MAPK, and PI3K/AKT signaling pathways, thereby contributing to pathological vascular remodeling [100]. In astrocytes, endothelins—particularly ET‐1—exert predominantly pro‐proliferative effects that are largely mediated by EDNRB [101, 102]. In this study, we revisited endothelin signaling in diffuse gliomas using a newly established panel of serum‐free cultured glioma cell lines representative of the major pathological subtypes—astrocytoma, oligodendroglioma, and glioblastoma. We combined this cellular resource with a broad range of high‐throughput approaches, including bulk and single‐cell RNA sequencing, spatial transcriptomics, proteomics, electrophysiology, as well as patient‐derived tumor extracts and sections. Through this integrative multi‐omics and functional strategy, we re‐evaluated the expression and regulation of EDNRA and EDNRB, their downstream signaling pathways, and the functional responses of glioma cells to endothelin stimulation. A synthesis of these findings is presented in the Graphical abstract.

Our data indicate that EDNRB is the predominant endothelin receptor in both patient‐derived glioma cell lines and primary tumors. This pattern is consistent with glioma large‐scale transcriptomic datasets (TCGA, REMBRANDT, and CGGA) (Fig. S11A–C), in which EDNRB expression consistently exceeds EDNRA across all tumor grades. Importantly, *EDNRB* expression declines with increasing tumor grade and fell further at recurrence, whereas *EDNRA* shows the opposite pattern, being enriched in high‐grade tumors, an opposing regulation associated with worse patient survival (Fig. S11B–D). Taken together, the convergence between our experimental results and multiple independent datasets highlights a grade‐ and subtype‐specific regulation of endothelin receptors in diffuse gliomas, supporting the notion that EDNRA and EDNRB may contribute differently to glioma biology, progression, and recurrence.

Diffuse gliomas are heterogeneous tumors composed of multiple co‐existing tumor cell states. Using patient samples harboring the IDH1 mutation together with an IDH1‐mutant cell line that recapitulates several of these states, we found that EDNRB expression is largely restricted to astrocyte‐like tumor cells, consistent with its known expression profile in the normal brain [102]. These astrocyte‐like tumor cells have previously been reported to exhibit lower proliferative activity than progenitor‐like and oligo‐progenitor‐like cells in GB [13, 103]. In line with this, analysis of the scRNA‐seq dataset from Neftel et al [13] showed that EDNRB expression displays minimal correlation with cyclins and  proliferation markers (*MKI67, PCNA*), whereas oligodendrocyte lineage genes (*OLIG1, OLIG2, ASCL1*) show stronger correlations, reflecting their preferential expression in actively dividing cell populations (Fig. S3C, correlation matrix) [13]. Consistent with these external datasets, our own scRNA‐seq analysis of an IDH1‐mutant glioma cell line showed that EDNRB‐positive cells occupy a less proliferative cellular state (Fig. S3B). This convergence of patient‐derived and *in vitro* data further supports a model in which EDNRB is preferentially associated with slow‐cycling or quiescent astrocyte‐like tumor populations [104].

The differential expression of EDNRB among tumor cell populations prompted us to investigate the signaling pathways that may influence its regulation. Our analyses showed that EDNRB expression increases upon growth factor withdrawal and following exposure to BMPs—factors secreted by glioma cells, reactive astrocytes, and endothelial cells [25]—both conditions that promote astrocytic differentiation programs [12]. In contrast, EDNRB is repressed by interferons, IL‐6–family cytokines, and activation of the Hippo–YAP pathway. We also observed that endothelin stimulation reduces EDNRB expression, suggesting the presence of an intrinsic negative feedback loop in which endothelins downregulate their own receptor. Taken together, these results demonstrate that EDNRB is subject to regulation by multiple, mechanistically distinct signaling cues that are likely present within the glioma microenvironment. This complex regulatory landscape likely contributes to the coexistence of EDNRB‐positive and EDNRB‐negative tumor cell populations within gliomas and may shape overall tumor cell heterogeneity.

Using patient tissue sections, we observed that EDNRA, in contrast, is primarily expressed by vascular cells—an observation consistent with previous reports [42]. However, our bioinformatic analyses revealed that a small subpopulation of GB cells with a pericyte‐like phenotype also express EDNRA. The presence of both tumor‐derived and non‐tumoral vascular cells expressing EDNRA in GB is likely to exacerbate vascular remodeling and thereby contribute to the aggressive behavior of these tumors. Actually, in the GB cell line we examined (Gb7), EDNRA expression was inducible by Notch1 signaling and, to a lesser extent, by hypoxia. EDNRA expression in Gb7 cells could also be detected *in vivo* by immunoPET imaging in a preclinical orthotopic xenograft model [105, 106]. This demonstrates the capacity of GB cells to upregulate EDNRA in response to specific pathways and environments. In contrast, this inducible capacity was absent in the IDH1‐mutant LGG275 line, where EDNRA remained undetectable upon Notch activation. We hypothesize that the well‐known DNA and chromatin hypermethylation associated with the IDH1‐mutant epigenetic context may prevent EDNRA activation in these tumors by the Notch pathway [107]. This interpretation is supported by our methylation analysis of publicly available datasets [108], which shows that the EDNRA locus and promoter are more heavily methylated in IDH1‐mutant gliomas compared with IDH1‐wild‐type tumors (Fig. S12A,B).

Functionally, activation of EDNRB by endothelins reduces proliferation, enhances migration, and reprograms gene expression toward a more mesenchymal profile. This shift coincides with (i) the repression of OPC‐associated genes such as OLIG1 and (ii) the activation of several downstream pathways, including Ca^2+^ signaling, ERK, and STAT3. The strong upregulation of STAT3 expression is particularly noteworthy, given its well‐established role in promoting mesenchymal transition in gliomas [17, 18]. At the electrophysiological level, we found that endothelins activate Ca^2+^‐dependent K^+^ currents that are sensitive to apamin, consistent with the involvement of SK channels. Both SK2 and SK3 are expressed in the LGG275 cell line used in our study. However, due to the very similar pharmacological profiles of these two channels, we were unable to determine which subtype is predominantly activated, and additional work will be required to resolve this point.

Our antiproliferative findings contrast with earlier reports that described endothelins as mitogenic in gliomas [31]. A likely explanation is methodological: most of those studies relied on serum‐cultured cell lines, where undefined serum components can profoundly reshape signaling networks and may mask the growth‐inhibitory effect of EDNRB activation observed under serum‐free, defined conditions.

Our study has several limitations. First, we did not assess whether EDNRB activation decreases proliferation and increases migration *in vivo*, and therefore, the physiological relevance of these effects remains to be established. Second, in patient‐derived samples, our WB analyses revealed the presence of multiple EDNRB isoforms, but their origin and functional significance could not be addressed here. It is possible that distinct isoforms exert different effects on glioma cell behavior [109]. Finally, previous work has reported a crosstalk between EDNRB and EGFR signaling in other biological contexts [110, 111], but this potential interaction was not explored in our study.

## 5 Conclusion

In conclusion, our work identifies endothelin signaling as an important—yet previously underappreciated—regulator of glioma cell state. EDNRB is the predominant endothelin receptor in tumor cells, whereas EDNRA is mainly expressed in vascular cells and in a minor subset of glioma cells. Functionally, EDNRB activation restrains proliferation while promoting a mesenchymal shift and increased migration through multiple signaling pathways. The contribution of G‐protein‐coupled receptors to the proneural‐to‐mesenchymal transition in glioma remains poorly defined [112]. To date, CXCR4—the receptor for SDF‐1/CXCL12—represents one of the few GPCRs known to drive PMT in gliomas [113], and our findings broaden this landscape by identifying EDNRB as an additional GPCR involved. Glioma cells located near blood vessels show reduced proliferation [22] and, since endothelial cells are a major source of endothelins, vessel‐derived endothelins likely contribute to this local growth restraint [114]. Consistently, high *EDNRB* expression correlates with longer patient survival (Fig. S11D). This raises the possibility that loss of EDNRB expression—through a mechanism that remains to be discovered—may enable tumor cells to evade microenvironment‐derived antiproliferative cues, thereby promoting progression. Addressing this question will be a central objective for future studies. From a therapeutic perspective, it will also be important to determine whether modulation of EDNRB signaling can be harnessed to limit proliferation or invasion in gliomas.

## Conflict of interest

A. Hb and D. B are co‐founders of Skymab Biotherapeutics.

## Author contributions

D.P and J‐P.H conceptualized the study and designed the methodology. D.P performed all the experiments with the help of other coauthors. L.G, H.A and A.Ha helped with cell culture, bulk RNA‐seq, qPCR. L.G performed flow cytometry experiments, provided data analysis for endothelin receptors expressions and helped with all the figures design. H.A helped with western blotting and HUVEC cocultures experiments. A.Ha performed qPCR for endothelin receptors expressions in hypoxia experiments. S.H and K.A.‐C helped with primary cell cultures, western blotting and immunostaining experiments. C.R performed immunostaining of glioma patient sections and contributed to imaging and data acquisition. A.Hb, M.H and D.B provided endothelin receptors, notably RB49 monoclonal antibody, CHO‐engineered cell lines and data analysis. V.A.‐C, L.R.G, C.G.‐B, F.D.B performed and analyzed migration assays on LGG275 cells. S.U. and M.S. contributed to the design, data analysis, and interpretation of all mass spectrometry experiments, including sample processing and proteomic data visualization. C.J, L.B, T.H, A.C, V.C, B.C, J.C provided Fura‐2‐AM calcium data and performed electrophysiological analyses of endothelin effects on SK2/SK3 channels (KCNN2/3). P.R, L.P, J‐P.P helped with EDNRB downstream signaling investigation using HTRF kits and ratiometric probes from experiments to data interpretation. M.Z, G.‐H.H, S.‐Q.L, L.Z provided help with patient sections, tumor microarray and data mining as for endothelin receptors explorations. M.A provided spatial transcriptomics data mining. H.D performed surgery on cases with diffuse low‐grade gliomas. L.Ba. performed surgery on cases with glioblastomas. V.R provided the diagnostics and clinical information of the cases. D.B, C.T, F.D, H.D, J‐P.H secured funding support (Funding Acquisition). J‐P.H and D.P wrote and corrected the manuscript. All authors reviewed and approved the final version of the manuscript.

## Supporting information


**Fig. S1.** EDNRB is preferentially expressed in astrocyte‐like glioma cells (related to Fig. 1).

**Fig. S2.** EDNRB is the predominant endothelin receptor and is expressed at the cell surface of diffuse glioma cultures (related to Fig. 2).

**Fig. S3.** EDNRB expression defines a low‐proliferative, astrocyte‐like cell population in gliomas. (related to Figs 1 and 2).

**Fig. S4.** Further characterization of ET‐1 effects on migration and cell death (related to Fig. 3).

**Fig. S5.** RNA‐seq analysis of glioma cell lines treated with ET‐1 (related to Fig. 4).

**Fig. S6.** ET‐1 induces shifts in the proteomic profile associated with mesenchymal transition signatures in LGG275 cells (related to Fig. 4).

**Fig. S7.** Signaling stimulated by endothelins in glioma cell lines (related to Fig. 5).

**Fig. S8.** Validation of ERK1/2 phosphorylation in LGG275 + GFs and of TDI activating YAP pathway in diffuse glioma cells (related to Fig. 6).

**Fig. S9.** EDNRA expression increases with glioma grade.

**Fig. S10.** Spatial mapping of endothelin receptors in glioblastoma.

**Fig. S11.** Endothelin receptor gene expression across human glioma datasets.

**Fig. S12.** EDNRA gene exhibits higher methylation levels in IDH‐mutant gliomas.

**Table S1.** Lists of all products and reagents.
**Table S2.** List of patient specimens.
**Table S3.** Glioma tissue microarray.
**Table S4.** Characteristics of the glioma cell lines.
**Table S5.** List of RT‐qPCR primers.
**Table S6.** List of antibodies.

**Table S7.** Differentially Expressed Genes (DEGs) identified by RNA‐seq in LGG275 cells following ET‐1 treatment.
**Table S8.** GSEA of RNA‐seq data from LGG275 cells following ET‐1 treatment.
**Table S9.** EnrichR analysis of RNA‐seq data from LGG275 cells following ET‐1 treatment.
**Table S10.** Differentially Expressed Genes (DEGs) identified by RNA‐seq of LGG336 cells following ET‐1 treatment.
**Table S11.** GSEA of RNA‐seq data from LGG336 cells following ET‐1 treatment.
**Table S12.** Differentially Expressed Genes (DEGs) identified by RNA‐seq in BT237 cells following ET‐1 treatment.
**Table S13.** GSEA of RNA‐seq data from BT237 cells following ET‐1 treatment.
**Table S14.** Differentially Expressed Genes (DEGs) identified by RNA‐seq of Gb4 cells following ET‐1 treatment.
**Table S15.** GSEA of RNA‐seq data from Gb4 cells following ET‐1 treatment.
**Table S16.** Differentially Expressed Genes (DEGs) identified by RNA‐seq in Gb7 cells following ET‐1 treatment.
**Table S17.** GSEA of RNA‐seq data from Gb7 cells following ET‐1 treatment.
**Table S18.** Differentially Expressed Genes (DEGs) identified by RNA‐seq in Gb21 cells following ET‐1 treatment.
**Table S19.** GSEA of RNA‐seq data from Gb21 cells following ET‐1 treatment.
**Table S20.** Correlation of ET‐1‐induced gene expression with Chanoch‐Myers mesenchymal signatures in endothelin‐receptor‐positive glioma lines.
**Table S21.** ET‐1 promotes astrocytic/mesenchymal markers and represses oligodendrocytic/proneural markers in endothelin‐receptor‐positive glioma lines.
**Table S22.** Proteomics identifies significant Differentially Expressed Proteins (DEPs) in LGG275 cells after ET‐1 treatment (quantified with same significativity in 3/5 experiments at least).
**Table S23.** Complete list of proteins quantified in LGG275 cells upon ET‐1 treatment (123 proteins) (with significativity in 3/5 experiments at least).
**Table S24.** EnrichR functional enrichment of proteomic DEPs in ET‐1–treated LGG275 cells.
**Table S25.** Gene Ontology enrichment (1D Fisher) of ET‐1–responsive proteins in LGG275 cells (5 experiments).

**Data S1.** Supplementary Material and Methods.

**Data S2.** Supplementary Figure legend.

## Data Availability

All raw and processed scRNA‐seq data generated in this study have been deposited in the Gene Expression Omnibus (GEO) under accession number GSE263796. All raw and processed RNA‐seq data generated in this study have been deposited in the Gene Expression Omnibus (GEO) under accession number such as GSE298185 (for LGG275 treated with compounds), GSE298282 (for glioma cell lines upon ET‐1), GSE298356 and GSE298357 (for LGG275 in coculture with endothelial cells), and GSE298358 (for Gb7 in coculture with endothelialcells). All proteomics data generated in this study have been deposited to the ProteomeXchange Consortium PRIDE database under the dataset identifier PXD065902.
