# Supplementary material for: EDNRB‐dependent endothelin signaling reduces proliferation and promotes proneural‐to‐mesenchymal transition in gliomas
Source: Mol Oncol. 2026 Apr 23;20(8):1933–62. doi: 10.1002/1878-0261.70223 (PMC13398683; doi:10.1002/1878-0261.70223)
Supplement: Supplementary file 2 [file MOL2-20-1933-s007.docx]

**Supplementary Tables**

**Table S1**: Lists of all products and reagents

| **Name** | **Reference** | **Final concentration** | **Supplier** |
| --- | --- | --- | --- |
| DMEM/F12 | #21331046 | / | Life Technologies |
| L-glutamine | #25030024 | 2 mM | ThermoFisher |
| B-27 without vitamin A (50X) | #12587010 | 1X | Life Technologies |
| N-2 (100X) supplement | #17502048 | 1X | Life Technologies |
| rhEGF | AF-100-15-1MG | 10 ng/mL | Peprotech, ThermoFisher |
| rhFGF2/bFGF | 100-18B-500UG | 10 ng/mL | Peprotech, ThermoFisher |
| heparin | #H3149 | 2 µg/mL | Sigma‐Aldrich |
| gentamicin | #11520506 | 10 µg/mL | Sigma‐Aldrich |
| ciprofloxacin | #PHR1044-1G | 2 µg/mL | Sigma‐Aldrich |
| fungin | #ant-fn-1 | 2 µg/mL | Invivogen |
| Poly-D-Lysine (PDL) | #P7886 | 25 µg/mL | Sigma‐Aldrich |
| Laminin (Lam) | #L2020 | 2 µg/cm² | Sigma‐Aldrich |
| Poly-HEMA  2-hydroxyethyl methacrylate | #192066 | 10 mg/mL | Sigma‐Aldrich |
| Hypoxia incubator | / | / | HeraCell Vios 160i |
| Trypsin-EDTA | #25200-056 | 0.25% | Life Technologies |
| DNAse I | #10104159001 | 10 mg/μl | Roche |
| CaCl_2_ | #10035-04-8 | 20 mM | Fisher Scientific |
| Trypsin inhibitor | #17075029 | 50 mg/mL | Life Technologies |
| Z2 cell counter | #Z2 9914591-D | / | Beckman Coulter |
| RNEasy kit | #74104 | / | Qiagen |
| TRIzol, | #15596018 | / | Thermo Fisher |
| KAPA SYBR Fast LC480 PCR kit | #KK4610 | / | Promega |
| Ultra-Turrax Disperser IKA | (RNA_extracts) | / | ThermoFisher |
| FastPrep-24 | (Protein_extracts) | / | MP_Biomedical |
| RIPA | #R0278 | / | Sigma-Aldrich |
| cOmplete™ Ultra Tablets | #05892970001 | / | Roche |
| PhosSTOP™, | #04906845001 | / | Roche |
| Pierce™ BCA Protein Assay Kit | #23225, #23227 |  | Thermo Fisher |
| Laemmli loading buffer | / | 1 X | Sigma-Aldrich |
| Amersham Protran nitrocellulose membranes (0.20 µm-pore size) | / | / | GE Healthcare |
| Mini-PROTEAN TGX Stain Free Gels 4-15%, | #4568086 | / | Bio-Rad |
| Ladder Kaleidoscope | #1610375 | / | Bio-Rad |
| EveryBlot BlockingBuffer | #12010020 | / | Bio-Rad |
| anti-rabbit IgG-HRP-linked | / | 1/2000 | GE Healthcare |
| Secondary HRP-linked antibodies | / | 1/2000 | Jackson Laboratory |
| Clarity Western ECL Substrate | #170-5060 | / | Bio-Rad |
| ChemiDoc MP Imaging System | / | / | Bio-Rad |
| Immobilon ECL | #WBULS0100 | / | Millipore-Sigma |
| GeneGnome XRQ Imaging System | / | / | Syngene |
| Poly-L-Ornithine | #P3655 | 1X | Millipore-Sigma |
| Nunclon Delta surface | #140685 | / | ThermoFisher |
| anti-Annexin V-AF647 antibody | #640912, | / | BioLegend |
| YO-PRO-1™ | #V13243 | / | ThermoFisher |
| Click-It EdU kit | #BCK-EdUFC647 | / | BaseClick |
| FcR blocking reagent | #130-059-901 | / | Miltenyi Biotec |
| TMT10 kit | #90110 | / | Thermo Fisher |
| nanoLC–MS/MS (RSLC U3000 + Exploris480 with FAIMS) | / | / | Thermo Fisher |
| HTRF IP-One Gq kit | #62IPAPE, | / | Cisbio, Revvity |
| HTRF Phospho-ERK1/2 kit | #64ERKPEG | / | Cisbio, Revvity |
| PheraStar | / | / | BMG, Labtech |
| Cal520-AM | #Cal-520® AM | 1,25 µM | AATBioquest |
| Fura-2AM | #F1201 | 6 µM | ThermoFisher |
| FluxORTM | #F20015 | / | ThermoFisher |
| FURA2-AM Lambda 421–equipped Olympus IX73 microscope with Zyla sCMOS camera | / | / | Olympus, Oxford instruments |
| FDSS µcell | / | / | Hamamatsu Photonics |

For compounds :

| **Name** | **Reference** | **Stock concentration** | **Dilution** | **Final concentration** | **Supplier** |
| --- | --- | --- | --- | --- | --- |
| ET-1 | E7764-10UG | 4µM (10µg/mL) | PBS+BSA 0,1% | Defined in the study | Sigma-Aldrich |
| ET-3 | E9137-10UG | 4µM (10µg/mL) | PBS+BSA 0,1% | Defined in the study | Sigma-Aldrich |
| IRL-1620 | SCP0135-500UG | 100 µM | DMSO | Defined in the study | Sigma-Aldrich |
| BQ-788 | HY-15894A | 10 mM | DMSO | Defined in the study | MedChemExpress |
| hOSM | 300-10 | 10 µg/mL | PBS+BSA 0,1% | 10 ng/mL | Peprotech |
| hLIF | 300-05 | 10 µg/mL | PBS+BSA 0,1% | 10 ng/mL | Peprotech |
| hCNTF | 450-13 | 10 µg/mL | PBS+BSA 0,1% | 10 ng/mL | Peprotech |
| hBMP2 | 120-02C | 10 µg/mL | PBS+BSA 0,1% | 10 ng/mL | Peprotech |
| hBMP4 | 120-05 | 10 µg/mL | PBS+BSA 0,1% | 10 ng/mL | Peprotech |
| hBMP6 | 120-06 | 10 µg/mL | PBS+BSA 0,1% | 10 ng/mL | Peprotech |
| hIFNβ | 300-02BC | 10 µg/mL | PBS+BSA 0,1% | 10 ng/mL | Peprotech |
| hIFNγ | 300-02 | 10 µg/mL | PBS+BSA 0,1% | 10 ng/mL | Peprotech |
| TDI-011536 | HY-150042 | 5 mM | DMSO | 5 µM | MedChemExpress |

**Table S2** : List of patient specimens

| **Patient samples name** | **Patient gender** | **Patient Age** | **Diagnosis**  **(subype, grade)** | **IDH1 status** | **Extracted from biological samples** | **Use for which experiments (EDNRA/EDNRB)** | **IF/IHC** |
| --- | --- | --- | --- | --- | --- | --- | --- |
| Gb39 | F | 50 | GB | IDH WT | Protein | Western Blot |  |
| Gb40 | F | 81 | GB | IDH WT | RNA, protein | RT-qPCR, Western Blot |  |
| Gb43 | M | 52 | GB | IDH WT | RNA, protein | RT-qPCR, Western Blot |  |
| LGG321 | M | 28 | A2 | IDH1R132H | RNA, protein | RT-qPCR, Western Blot |  |
| LGG330 | M | 37 | A2 | IDH1R132H | RNA, protein | RT-qPCR, Western Blot |  |
| LGG250 | F | 40 | A2/3 | IDH1R132H | RNA, protein | RT-qPCR, Western Blot |  |
| LGG348 | F | 25 | A3 | IDH1R132H | RNA, protein | RT-qPCR, Western Blot, Cryosections | EDNRB/APOE |
| LGG309 | F | 61 | A3 | IDH1R132H | RNA | RT-qPCR |  |
| LGG309 (2) | F | 61 | A3 | IDH1R132H | RNA | RT-qPCR |  |
| LGG356 | M | 39 | A3 | IDH1R132H | RNA, protein | RT-qPCR, Western Blot |  |
| LGG322 | M | 52 | O2/3 | IDH1R132H | RNA | RT-qPCR |  |
| LGG346 | M | 45 | O2/3 | IDH1R132H | RNA, protein | RT-qPCR, Western Blot, Cryosections | EDNRB/IDH1R132H, Ctrl |
| LGG358 | M | 43 | O2 ou O3 | IDH1R132H | RNA, protein | RT-qPCR |  |
| LGG351 | M | 60 | O3 | IDH1R132H | RNA, protein | RT-qPCR, Western Blot |  |
| LGG357 | M | 29 | O2 (/O3) | IDH1R132H | RNA, protein | RT-qPCR, Western Blot, Cryosections | EDNRB/IDH1R132H |
| LGG357bis | M | 29 | O2 (/O3) | IDH1R132H | RNA | RT-qPCR | EDNRB/APOE ; EDNRB/OLIG2 |
| LGG359 | F | 62 | O3 | IDH1R132H | RNA, protein | RT-qPCR, Western Blot |  |
| LGG361 | M | 40 | O3 | IDH1R132H | Protein | Western Blot, Cryosections | EDNRB/IDH1R132H, Ctrl |
| LGG362 | M | 25 | O3 | IDH1R132H | Protein | Western Blot |  |
| LGG318 | M | 26 | A2 | IDH1R132H | Protein | Western Blot |  |
| LGG309 | F | 61 | A3 | IDH1R132H | Protein | Western Blot |  |
| LGG355 | M | 44 | A4 | IDH1R132H | Protein | Western Blot |  |
| LGG244 | M | 40 | A2 | IDH1R132H | Sections | Cryosections | EDNRB/OLIG2 |
| Gb34-A | M | 60 | GB | IDH WT | Protein | Western Blot |  |
| LGG 180 (180-1) | F | 35 | O2 | IDH1R132H | Protein | Western Blot |  |
| LGG184 (184-O) | F | 38 | A2 | IDH1R132H | Protein | Western Blot |  |
| LGG187 (187-1) | M | 26 | A2 | IDH1R132H | Protein | Western Blot |  |
| LGG182 (182) | M | 25 | A2/3 | IDH1R132H | Protein | Western Blot |  |
| LGG190 (190-2) | M | 42 | A2/3 | IDH1R132H | Protein | Western Blot |  |
| LGG189 (189-2) | F | 27 | A3 | IDH1R132H | Protein | Western Blot |  |
| LGG93 | N.A | N.A | O3 | IDH1R132H | Protein | Western Blot |  |
| LGG185 (185) | M | 39 | A4 | IDH1R132H | Protein | Western Blot |  |
| LGG56 (56) | M | 33 | A4 | IDH1R132H | Protein | Western Blot |  |
| LGG85 (85) | M | 38 | A4 | IDH1R132H | Protein | Western Blot |  |
| LGG316 | M | 59 | O2 | IDH1R132H | Protein | Western Blot |  |
| Meningioma | N.A | N.A | / | / | Protein | Western Blot |  |
| Human Brain | N.A | N.A | / | / | Protein | Western Blot |  |

(A= Astrocytomas, O=Oligodendrogliomas, GB = Glioblastomas)

**Table S3**: Glioma tissue microarray [1], [2].

|  | **Case no** | **Gender** | **Age** | **Diagnosis** | **WHO grade** | **Core 1** | **Core 2** |
| --- | --- | --- | --- | --- | --- | --- | --- |
| **1** | 3 | F | 37 | Anaplastic astrocytoma | 3 | 0 | 0 |
| **2** | 4 | F | 57 | Diffuse astrocytoma | 2 | 1 | 0 |
| **3** | 6 | M | 40 | Anaplastic oligodendroglioma | 3 |  |  |
| **4** | 7 | F | 65 | Oligodendroglioma | 2 |  |  |
| **5** | 26 | M | 57 | Control WM & GM |  | 1 | 1 |
| **6** | 27 | M | 34 | Anaplastic oligoastrocytoma | 3 | 1 | 0 |
| **7** | 33 | M | 54 | Glioblastoma | 4 | 1 | 2 |
| **8** | 34 | F | 67 | Anaplastic astrocytoma | 3 | 1 | 1 |
| **9** | 35 | M | 83 | Diffuse astrocytoma | 2 | 1 | 0 |
| **10** | 37 | F | 70 | Anaplastic oligodendroglioma | 3 |  |  |
| **11** | 38 | M | 29 | Oligodendroglioma | 2 |  |  |
| **12** | 55 | M | 28 | Control WM & GM |  | 1 | 0 |
| **13** | 56 | M | 49 | Anaplastic oligoastrocytoma | 3 | 1 | 2 |
| **14** | 60 | M | 65 | Glioblastoma | 4 | 0 | 1 |
| **15** | 61 | F | 31 | Anaplastic astrocytoma | 3 | 0 | 0 |
| **16** | 62 | M | 27 | Diffuse astrocytoma | 2 | 0 | 0 |
| **17** | 64 | F | 60 | Anaplastic oligodendroglioma | 3 |  |  |
| **18** | 76 | M | 38 | Control WM & GM |  | 0 | 0 |
| **19** | 77 | F | 61 | Anaplastic oligoastrocytoma | 3 | 1 | 0 |
| **20** | 80 | F | 29 | Anaplastic astrocytoma | 3 | 0 | 0 |
| **21** | 81 | M | 32 | Diffuse astrocytoma | 2 | 0 | 0 |
| **22** | 83 | F | 42 | Anaplastic oligodendroglioma | 3 |  |  |
| **23** | 84 | F | 65 | Oligodendroglioma | 2 |  |  |
| **24** | 92 | F | 24 | Anaplastic oligoastrocytoma | 3 | 1 | 0 |
| **25** | 93 | F | 65 | Glioblastoma | 4 | 2 | 2 |
| **26** | 94 | F | 46 | Anaplastic astrocytoma | 3 | 2 | 2 |
| **27** | 95 | M | 48 | Diffuse astrocytoma | 2 | 0 | 0 |
| **28** | 102 | M | 34 | Anaplastic oligoastrocytoma | 3 | 0 | 0 |
| **29** | 103 | F | 57 | Glioblastoma | 4 | 1 | 2 |
| **30** | 104 | M | 41 | Anaplastic astrocytoma | 3 | 0 | 1 |
| **31** | 105 | F | 42 | Diffuse astrocytoma | 2 | 0 | 0 |
| **32** | 106 | F | 34 | Anaplastic oligodendroglioma | 3 |  |  |
| **33** | 112 | F | 63 | Glioblastoma | 4 | 2 | 2 |
| **34** | 113 | F | 37 | Anaplastic astrocytoma | 3 | 1 |  |
| **35** | 114 | M | 26 | Diffuse astrocytoma | 2 | 1 | 1 |
| **36** | 115 | M | 42 | Anaplastic oligodendroglioma | 3 |  |  |
| **37** | 116 | M | 42 | Glioblastoma | 4 | 1 | 1 |
| **38** | 117 | F | 64 | Glioblastoma | 4 | 2 | 2 |
| **39** | 120 | M | 55 | Glioblastoma | 4 | 0 | 2 |
|  | | | | | |  |  |

Annotation: 0 = no vascular staining; 1 = minority of vessels stained; 2 = majority of vessels stained

**Table S4: Characteristics of the glioma cell lines**

| **Cell lines** | **Patient**  **(Sex-Age)** | **Tumor information** | **Grade**  **(WHO 2016)** | **IDH1**  **status** | **Miscellaneous information** | **Alterations** | **Publication status** |
| --- | --- | --- | --- | --- | --- | --- | --- |
| LGG275 | F40 | Recurrent primary Tumor (reoperation of 2014) | Grade II / III astrocytoma | IDH1R132H | Astrocytoma grade II according to histology astrocytoma high grade according to molecular annotation | ATRX lost Presence in 3 copies of EGFR, MYC~~,~~ Braf, c-Met, loss of one copy of CDKN2A | Augustus et al, 2021 |
| LGG336 | F40 | Recurrent primary tumor (reoperation of 2012) | Grade II / III astrocytoma | IDH1R132H | Astrocytoma grade II according to histology | c-Met and Braf 3 copies, loss of 9q | *Unpublished (Garcia et al, in preparation)* |
| LGG85 | M38 | Recurrent primary tumor (reoperation of 2012) | Grade IV astrocytoma | IDH1R132H | TMZ | c-Met neg | Leventoux et al, 2020 |
| LGG349 | F57 | Recurrent primary Tumor (reoperation of 2018) | Grade IV astrocytoma | IDH1R132H initially but mutation loss in culture | multiple cycles of PCV, radiotherapy + TMZ | ATRX maintained MGMT methylation, unmutated TERT | *Unpublished (Garcia et al, in preparation)* |
| BT138 | M50 | Recurrent primary Tumor (reoperation) | Grade III Oligodendroglioma (NOS) | IDH1R132H initially but mutation loss in culture | Tumor in the left frontal lobe |  | Koivunen et al, 2012 |
| BT237 | F43 | Recurrent primary Tumor (reoperation) | Grade III Oligodendroglioma | IDH1R132H | Tumor in the left frontal lobe |  | Koivunen et al, 2012 |
| BT054 | F49 | Recurrent primary Tumor | Grade III Oligodendroglioma | IDH1R132H | TMZ and radiotherapy Tumor in the right frontal lobe | MGMT methylation | Kelly et al, 2010; Luchman et al, 2012; Mazor et al, 2015; Yuan et al, 2018 |
| BT088 | M50 | Recurrent primary Tumor (reoperation) | Grade III Oligodendroglioma | IDH1R132H initially but mutation loss in culture | Tumor in the right frontal lobe Several cycles of PCV, radiotherapy+TMZ |  | Kelly et al, 2010; Luchman et al, 2012; Mazor et al, 2015; Yuan et al, 2018 |
| Gb4 | M53 | Primary Tumor | Grade IV Glioblastoma | IDH1 WT | Mesenchymal |  | Guichet et al, 2013  Guichet et al, 2015 |
| Gb5 | M64 | Primary Tumor | Grade IV Glioblastoma | IDH1 WT | GB with oligodendroglial component |  | Guichet et al, 2013  Guichet et al, 2016 |
| Gb7 | M52 | Primary Tumor | Grade IV Glioblastoma | IDH1 WT | Proneural |  | Guichet et al, 2013; Guichet et al, 2015 |
| Gb21 | F53 | Primary Tumor | Grade IV Glioblastoma | IDH1 WT | Giant Cell GB |  | Guichet et al, 2016;  Guelfi et al, 2021 |

**Table S5 :** List of RT-qPCR primers

| **Gene** | **Forward Primer** | **Reverse Primer** |
| --- | --- | --- |
| ACTB | GGACTTCGAGCAAGAGATGG | AGCACTGTGTTGGCGTACAG |
| **EDNRA** | TCAAGATGGAAACCCTTTGC | ATTGAGCCATTGCTGGGTAG |
| **EDNRB** | ATGACGCCACCCACTAAGAC | GAACACAAGGCAGGACACAA |
| CD44 | CAATAGCACCTTGCCCACAAT | AATCACCACGTGCCCTTCTATG |
| CTGF | CAGCATGGACGTTCGTCTG | AACCACGGTTTGGTCCTTGG |
| OLIG1 | CGCAGAGAGTTTTCGCTCTT | GCGGTTGGTTTTCGTTTTTA |
| OLIG2 | GACAAGCTAGGAGGCAGTGG | CGGCTCTGTCATTTGCTTCT |
| MKI67 | CCCCCACCAGAACTAACAGA | ACTTTGATGCCCTCATCACC |
| STAT3 | CAGCAGCTTGACACACGGTA | AAACACCAAAGTGGCATGTGA |
| NOTCH1 | TCCACCAGTTTGAATGGTCA | CGCAGAGGGTTGTATTGGTT |
| KCNN3 | CTGCCGCCAAAATAAACATT | GCCTGGCACAAGCTTTCTAC |

**Table S6** **:** List of antibodies

| **Name** | **Species** | **Reference** | **Manufacturer** | **Dilution** |
| --- | --- | --- | --- | --- |
| EDNRA | Rabbit | ab178454 | Abcam | 1:1000 |
| EDNRB | Sheep | AF4496-SP | R&D Systems | 1:500 |
| RB49 | Mouse | CEA | from Dr Boquet & Dr Herbet | 1:500 |
| OLIG1 | Goat | AF2417 | R&D Systems | 1:300 |
| OLIG2 | Mouse | MABN50 | Sigma | 1:500 |
| ASCL1 | Rabbit | ab211327 | Abcam | 1:100 |
| GFAP | Rabbit | Z0334 | Dako | 1:5000 |
| AQP4 | Rabbit | 16473-1-AP | Proteintech | 1:500 |
| APOE | Rabbit | EP1374Y | Abcam | 1:1000 |
| pSMAD1/5 | Rabbit | 9516 | Cell Signalling | 1:800 |
| IDH1R132H | Mouse | DIA−H09 | Dianova | 1:100 |
| CD44-PE | Rabbit | 130-110-394 | Miltenyi Biotec | 1:100 |
| CTGF | Rabbit | Ab209780 | Abcam | 1:500 |
| pSTAT3 | Rabbit | 9145S | Cell Signalling | 1:1000 |
| STAT3 | Rabbit | A19566 | ABClonal | 1:1000 |
| pERK1/2 | Rabbit | 9910 | Cell Signalling | 1:1000 |
| ERK1/2 | Rabbit | 9102 | Cell Signalling | 1:1000 |
| KCNN3/SK3 | Rabbit | H-45 | Santa Cruz Biotechnology | 1:100 |
| KCNN2/SK2 | Rabbit | APC-028 | Alomone Labs | 1:200 |
| α-actin | Rabbit | A2066 | Sigma-Aldrich | 1:5000 |
| β-actin | Mouse | 8H10D10 | Novus | 1:2500 |

**Supplementary references for Table S3**

[1]	L. Zhang *et al.*, “Pleiotrophin promotes vascular abnormalization in gliomas and correlates with poor survival in patients with astrocytomas.,” *Sci. Signal.*, vol. 8, no. 406, p. ra125, Dec. 2015, doi: 10.1126/scisignal.aaa1690.

[2]	S. N. Popova *et al.*, “Subtyping of gliomas of various WHO grades by the application of immunohistochemistry.,” *Histopathology*, vol. 64, no. 3, pp. 365–79, Feb. 2014, doi: 10.1111/his.12252.
