# Supplementary figures and images for: EDNRB‐dependent endothelin signaling reduces proliferation and promotes proneural‐to‐mesenchymal transition in gliomas
Source: Mol Oncol. 2026 Apr 23:10.1002/1878-0261.70223. Online ahead of print. doi: 10.1002/1878-0261.70223 (PMC13398683; doi:10.1002/1878-0261.70223)

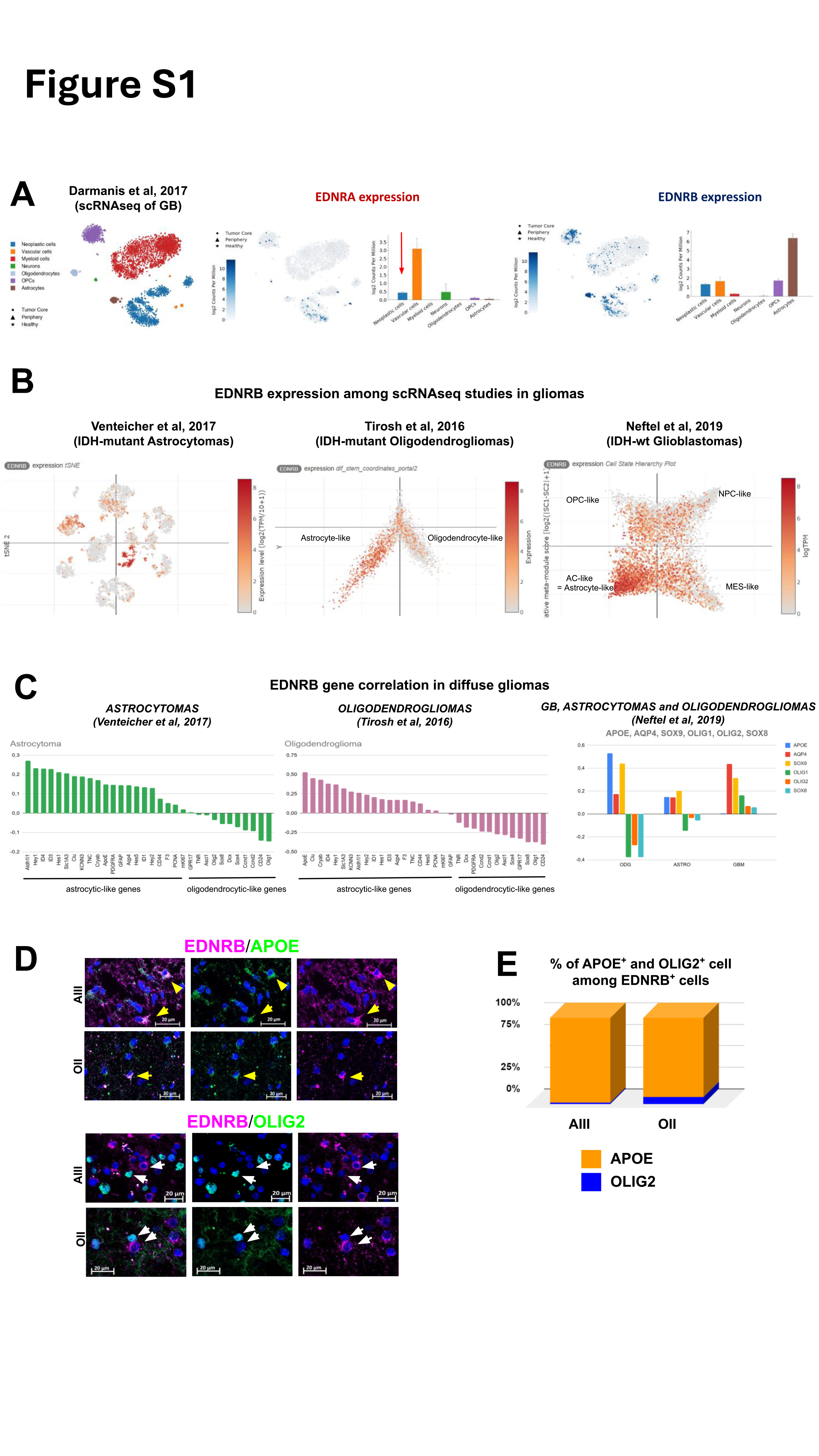

Supplement: Supplementary file 1 — Fig. S1. EDNRB is preferentially expressed in astrocyte‐like glioma cells (related to Fig. 1). [file MOL2-9999-0-s014.tif]

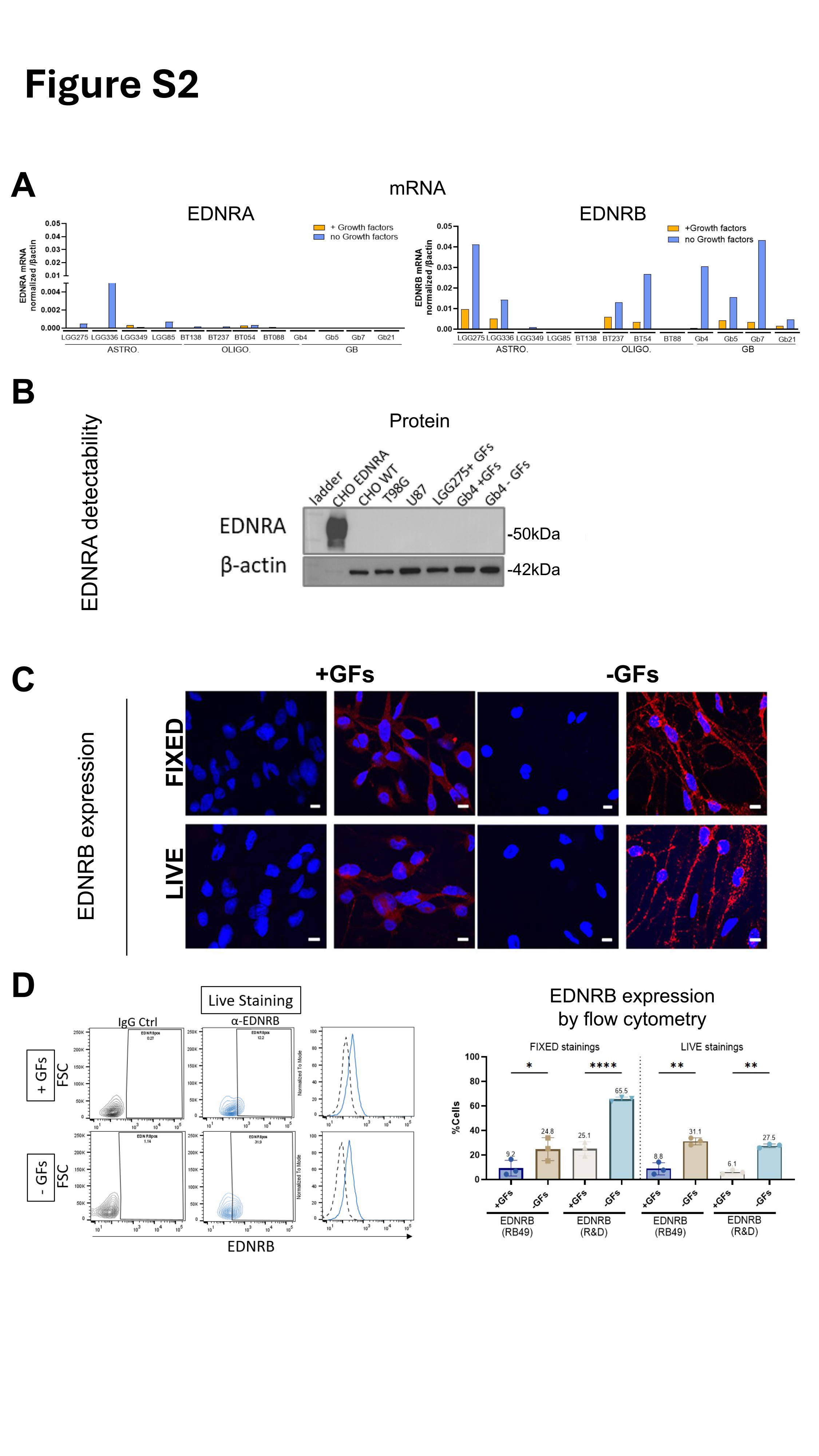

Supplement: Supplementary file 2 — Fig. S2. EDNRB is the predominant endothelin receptor and is expressed at the cell surface of diffuse glioma cultures (related to Fig. 2). [file MOL2-9999-0-s012.tif]

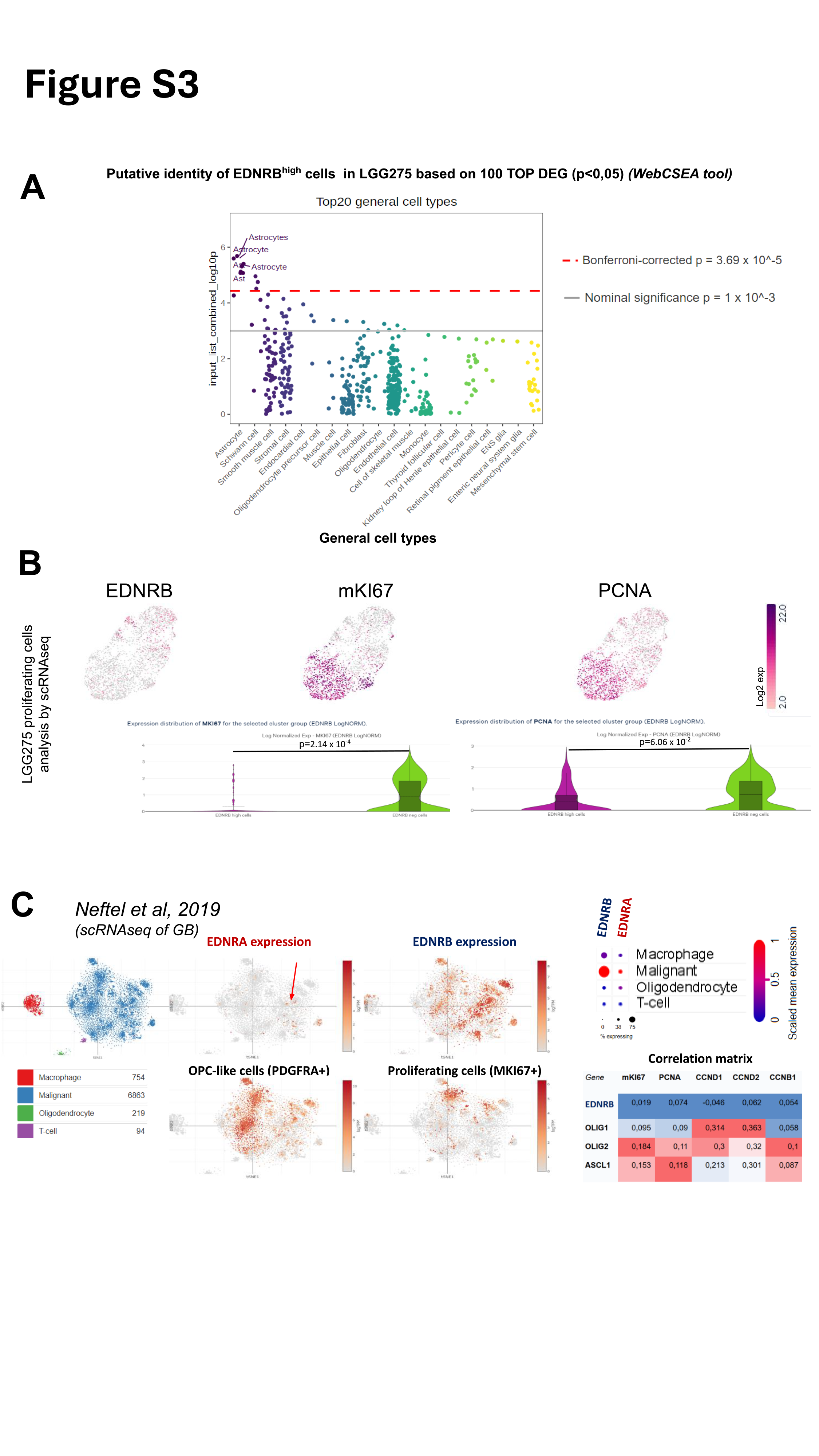

Supplement: Supplementary file 3 — Fig. S3. EDNRB expression defines a low‐proliferative, astrocyte‐like cell population in gliomas. (related to Figs 1 and 2). [file MOL2-9999-0-s001.tif]

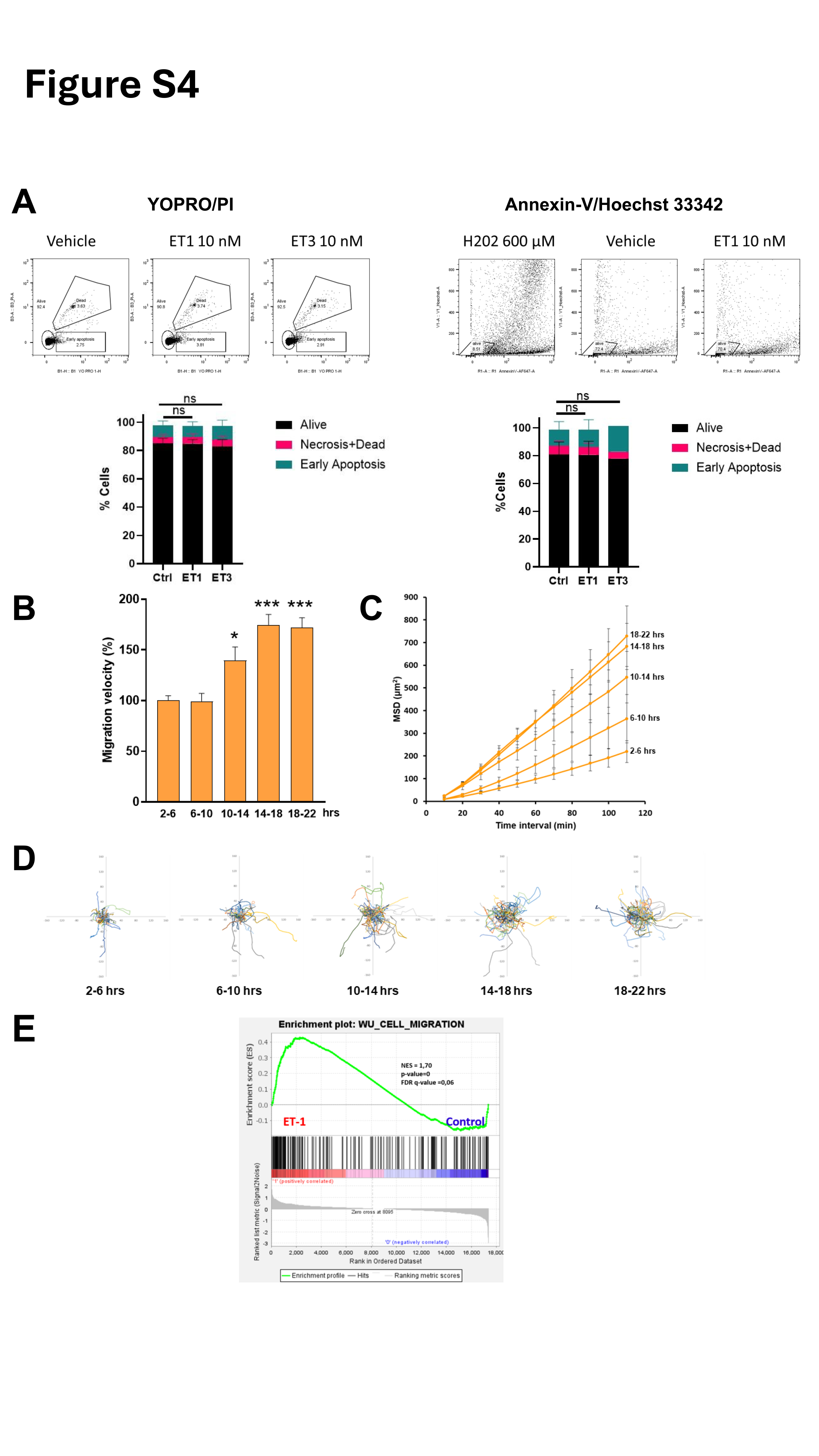

Supplement: Supplementary file 4 — Fig. S4. Further characterization of ET‐1 effects on migration and cell death (related to Fig. 3). [file MOL2-9999-0-s010.tif]

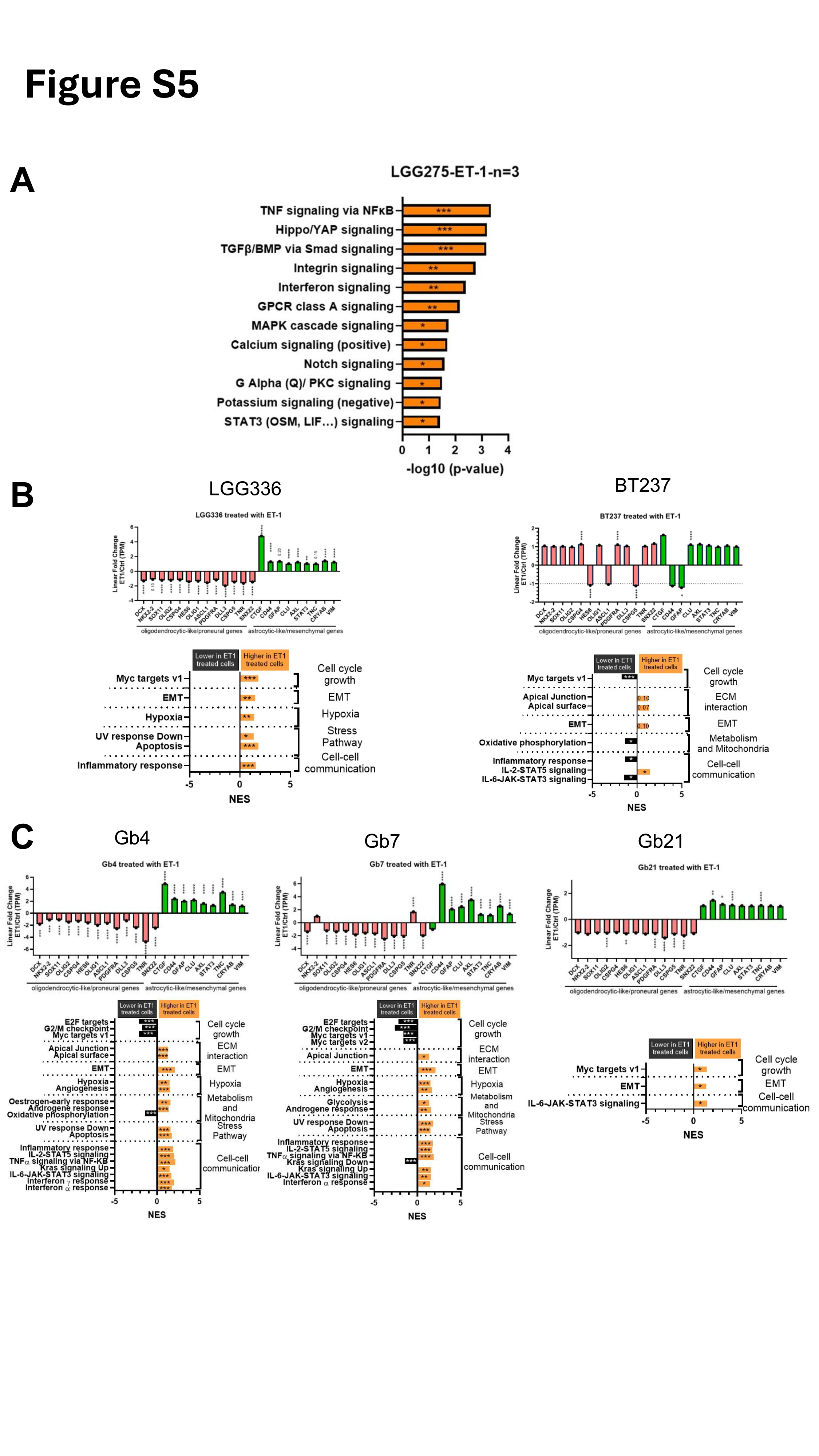

Supplement: Supplementary file 5 — Fig. S5. RNA‐seq analysis of glioma cell lines treated with ET‐1 (related to Fig. 4). [file MOL2-9999-0-s007.tif]

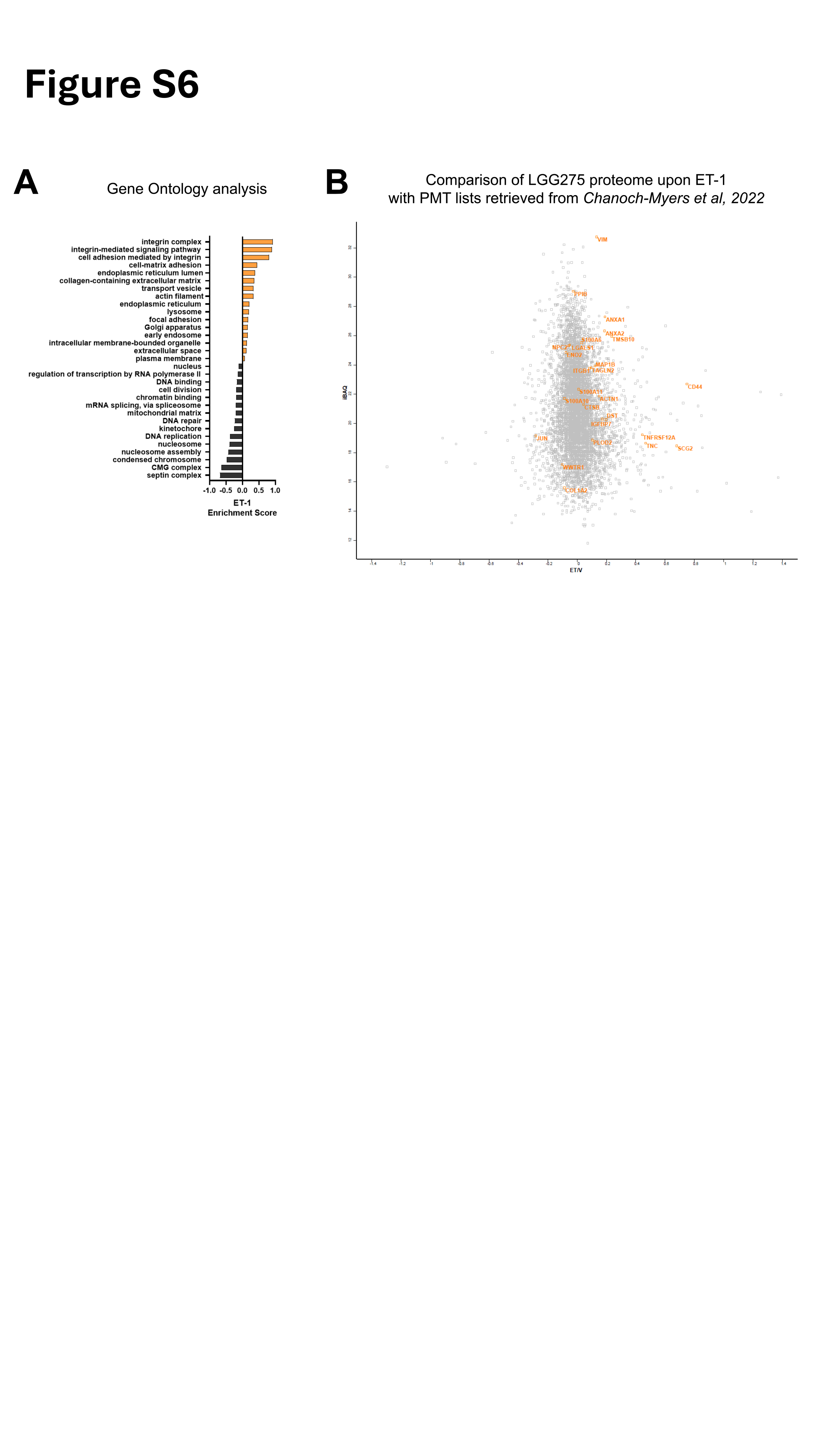

Supplement: Supplementary file 6 — Fig. S6. ET‐1 induces shifts in the proteomic profile associated with mesenchymal transition signatures in LGG275 cells (related to Fig. 4). [file MOL2-9999-0-s004.tif]

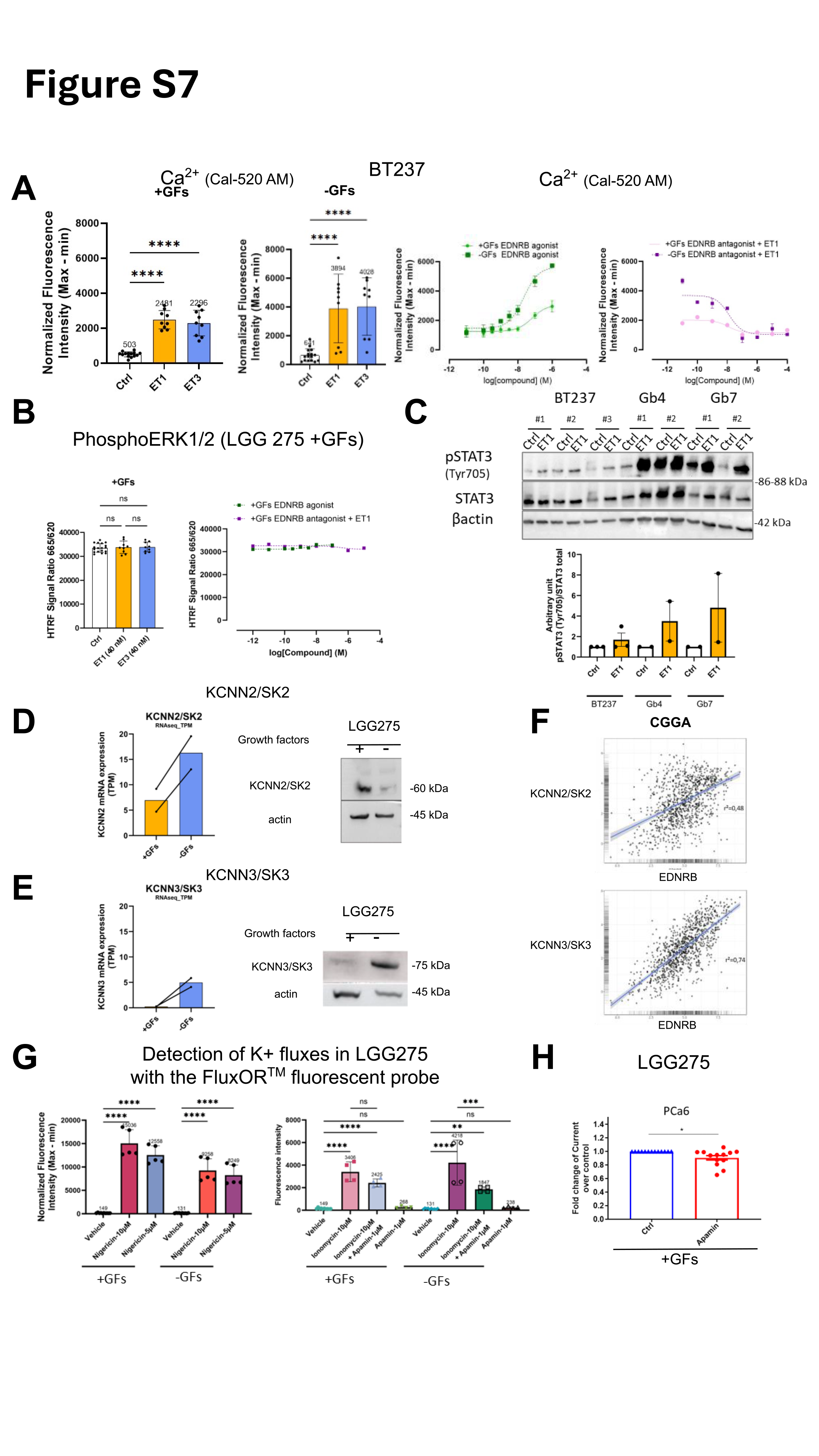

Supplement: Supplementary file 7 — Fig. S7. Signaling stimulated by endothelins in glioma cell lines (related to Fig. 5). [file MOL2-9999-0-s009.tif]

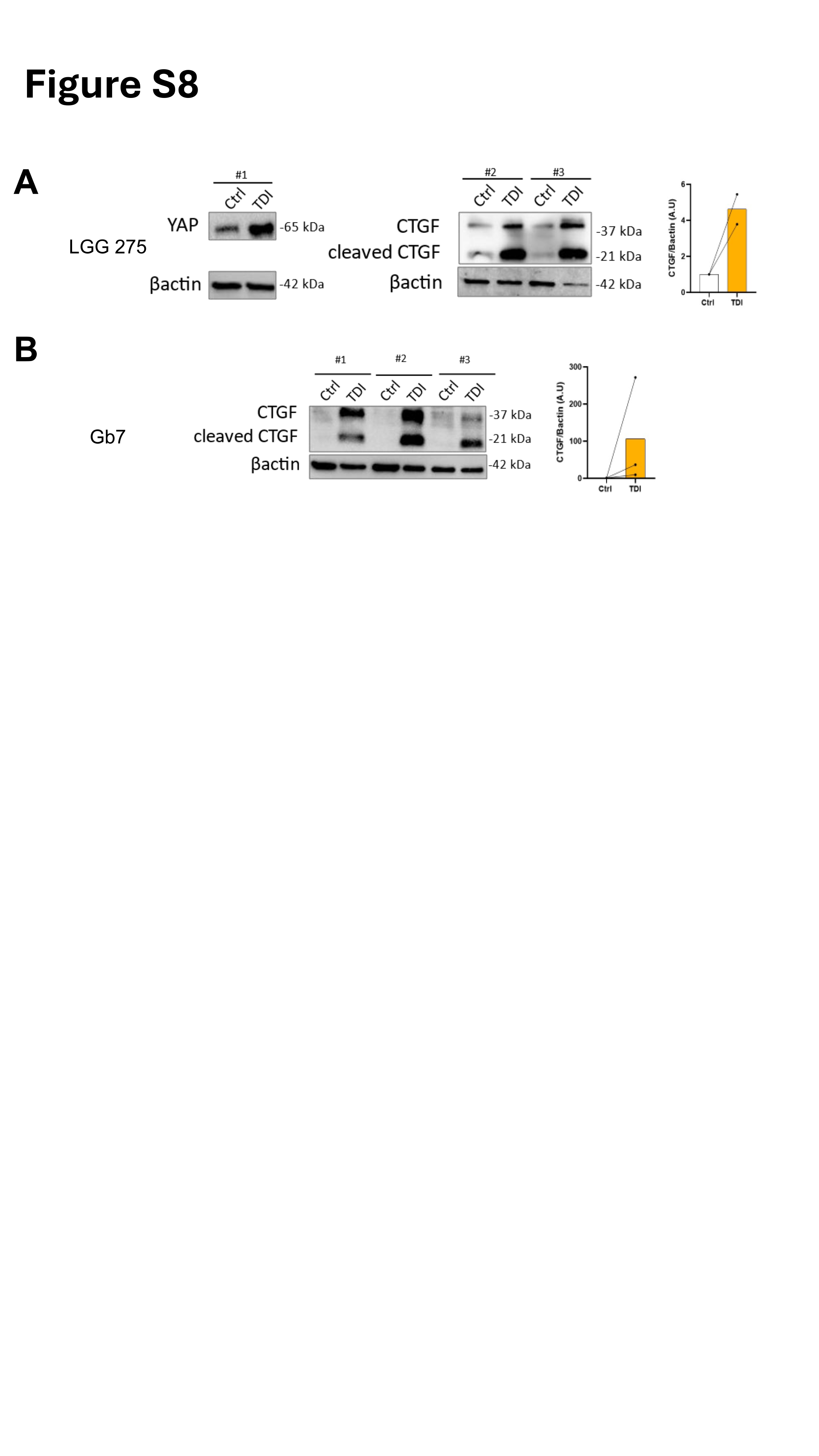

Supplement: Supplementary file 8 — Fig. S8. Validation of ERK1/2 phosphorylation in LGG275 + GFs and of TDI activating YAP pathway in diffuse glioma cells (related to Fig. 6). [file MOL2-9999-0-s003.tif]

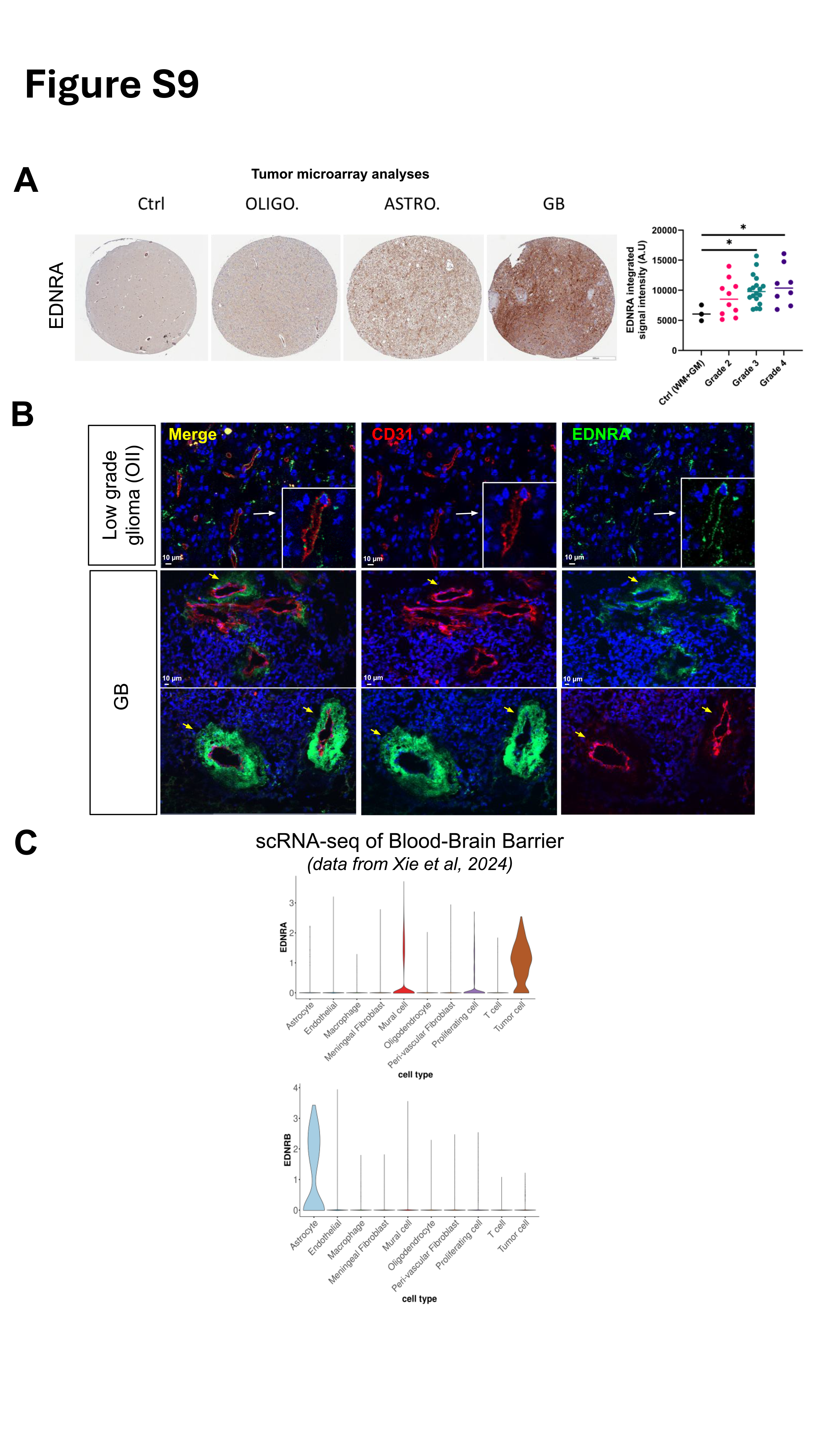

Supplement: Supplementary file 9 — Fig. S9. EDNRA expression increases with glioma grade. [file MOL2-9999-0-s013.tif]

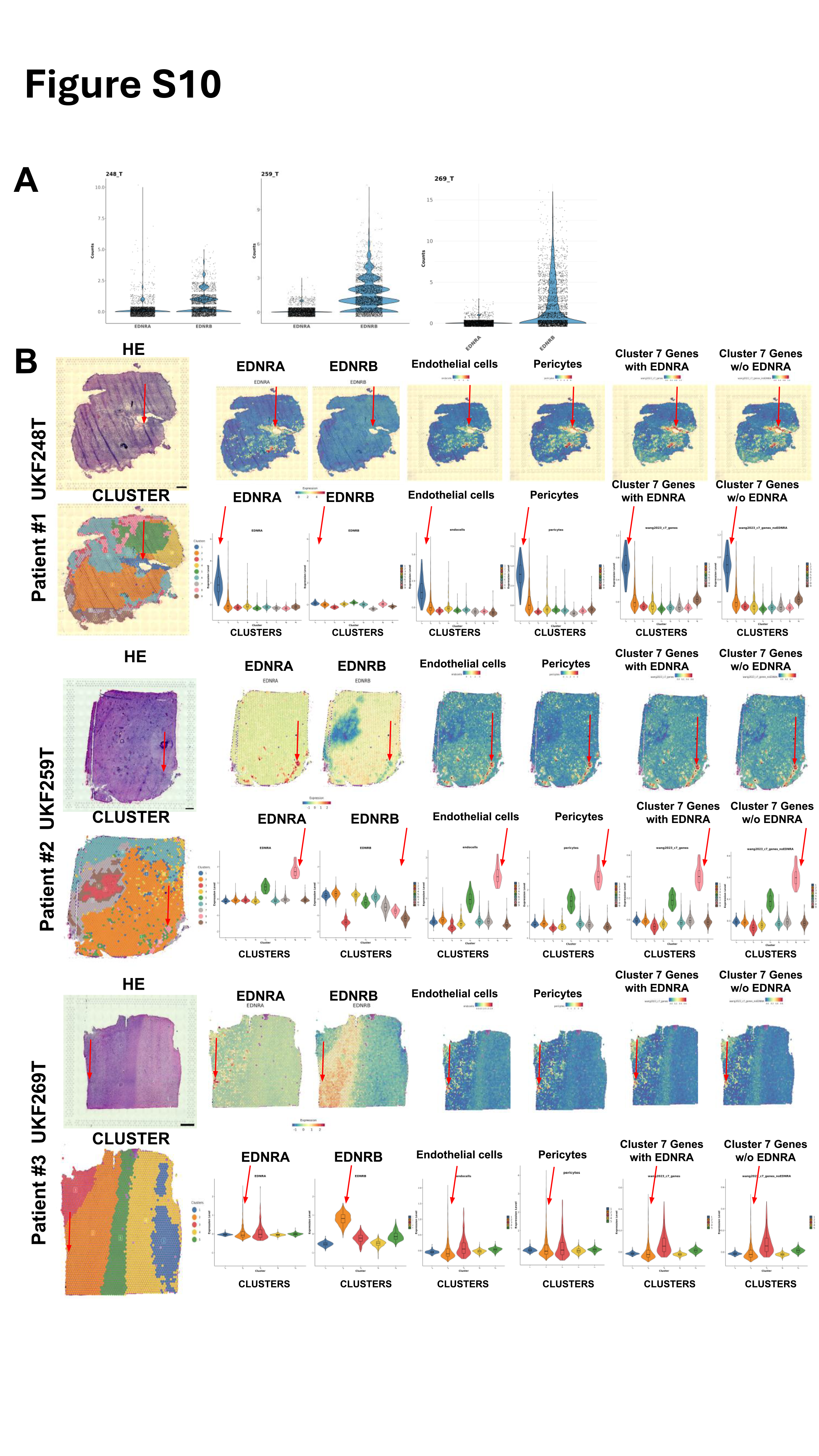

Supplement: Supplementary file 10 — Fig. S10. Spatial mapping of endothelin receptors in glioblastoma. [file MOL2-9999-0-s008.tif]

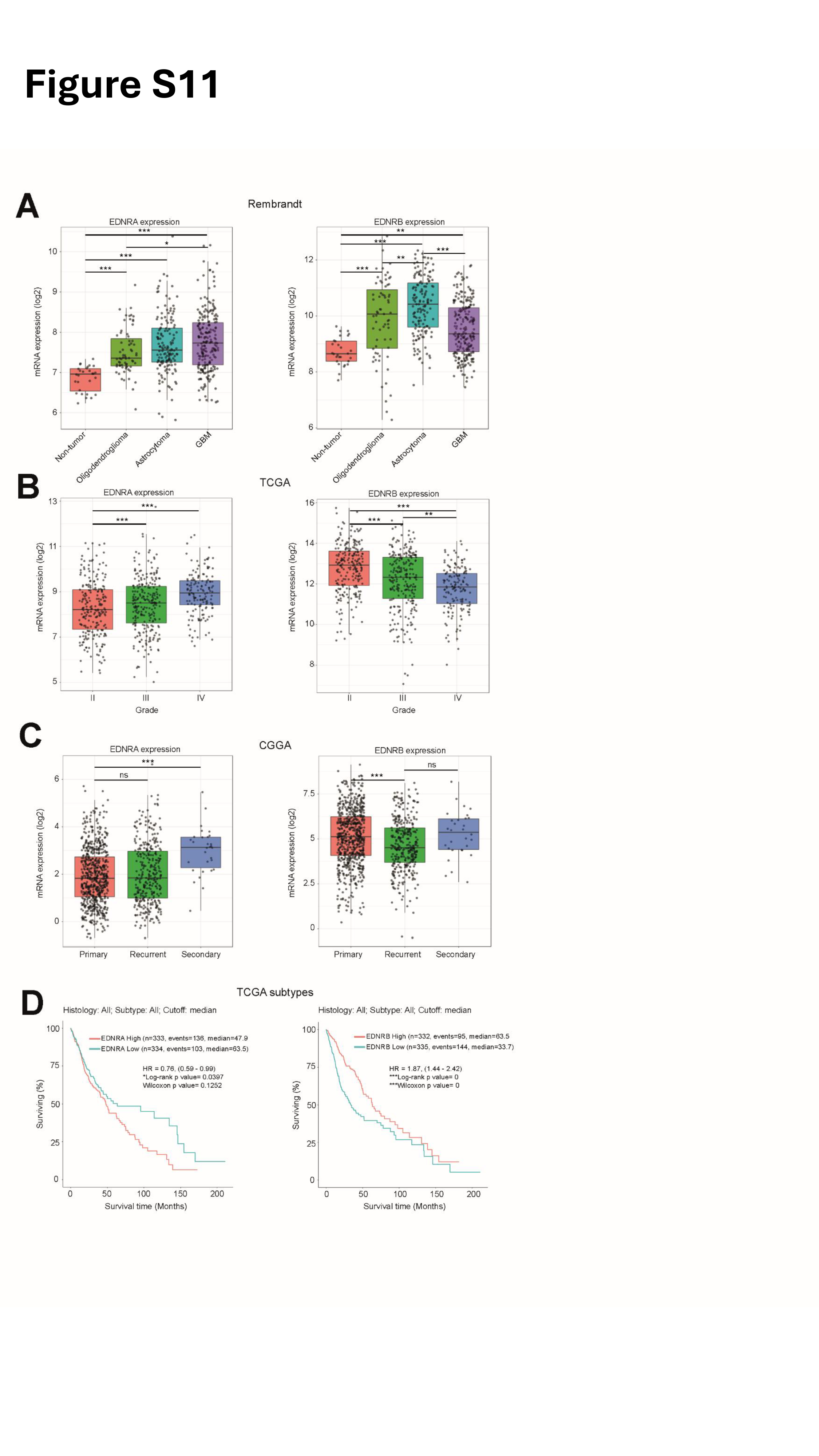

Supplement: Supplementary file 11 — Fig. S11. Endothelin receptor gene expression across human glioma datasets. [file MOL2-9999-0-s002.tif]

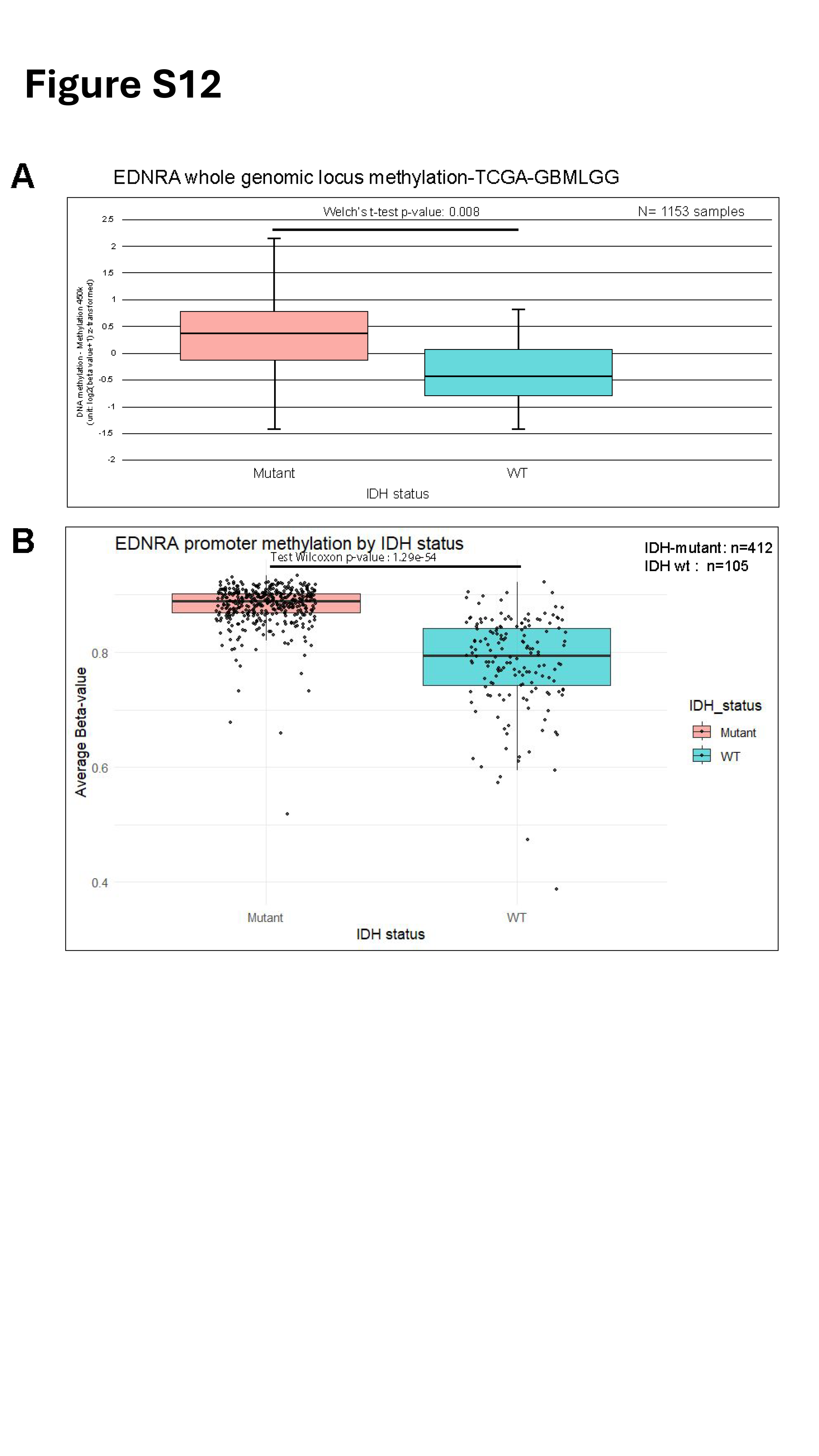

Supplement: Supplementary file 12 — Fig. S12. EDNRA gene exhibits higher methylation levels in IDH‐mutant gliomas. [file MOL2-9999-0-s006.tif]
