# Supplementary material for: EDNRB‐dependent endothelin signaling reduces proliferation and promotes proneural‐to‐mesenchymal transition in gliomas
Source: Mol Oncol. 2026 Apr 23:10.1002/1878-0261.70223. Online ahead of print. doi: 10.1002/1878-0261.70223 (PMC13398683; doi:10.1002/1878-0261.70223)
Supplement: Supplementary file 13 — Table S1. Lists of all products and reagents. Table S2. List of patient specimens. Table S3. Glioma tissue microarray. Table S4. Characteristics of the glioma cell lines. Table S5. List of RT‐qPCR primers. Table S6. List of antibodies. [file MOL2-9999-0-s005.docx]

**Supplementary Tables**

**Table S1**: Lists of all products and reagents

| **Name** | **Reference** | **Final concentration** | **Supplier** |
| --- | --- | --- | --- |
| DMEM/F12 | #21331046 | / | Life Technologies |
| L-glutamine | #25030024 | 2 mM | ThermoFisher |
| B-27 without vitamin A (50X) | #12587010 | 1X | Life Technologies |
| N-2 (100X) supplement | #17502048 | 1X | Life Technologies |
| rhEGF | AF-100-15-1MG | 10 ng/mL | Peprotech, ThermoFisher |
| rhFGF2/bFGF | 100-18B-500UG | 10 ng/mL | Peprotech, ThermoFisher |
| heparin | #H3149 | 2 µg/mL | Sigma‐Aldrich |
| gentamicin | #11520506 | 10 µg/mL | Sigma‐Aldrich |
| ciprofloxacin | #PHR1044-1G | 2 µg/mL | Sigma‐Aldrich |
| fungin | #ant-fn-1 | 2 µg/mL | Invivogen |
| Poly-D-Lysine (PDL) | #P7886 | 25 µg/mL | Sigma‐Aldrich |
| Laminin (Lam) | #L2020 | 2 µg/cm² | Sigma‐Aldrich |
| Poly-HEMA  2-hydroxyethyl methacrylate | #192066 | 10 mg/mL | Sigma‐Aldrich |
| Hypoxia incubator | / | / | HeraCell Vios 160i |
| Trypsin-EDTA | #25200-056 | 0.25% | Life Technologies |
| DNAse I | #10104159001 | 10 mg/μl | Roche |
| CaCl_2_ | #10035-04-8 | 20 mM | Fisher Scientific |
| Trypsin inhibitor | #17075029 | 50 mg/mL | Life Technologies |
| Z2 cell counter | #Z2 9914591-D | / | Beckman Coulter |
| RNEasy kit | #74104 | / | Qiagen |
| TRIzol, | #15596018 | / | Thermo Fisher |
| KAPA SYBR Fast LC480 PCR kit | #KK4610 | / | Promega |
| Ultra-Turrax Disperser IKA | (RNA_extracts) | / | ThermoFisher |
| FastPrep-24 | (Protein_extracts) | / | MP_Biomedical |
| RIPA | #R0278 | / | Sigma-Aldrich |
| cOmplete™ Ultra Tablets | #05892970001 | / | Roche |
| PhosSTOP™, | #04906845001 | / | Roche |
| Pierce™ BCA Protein Assay Kit | #23225, #23227 |  | Thermo Fisher |
| Laemmli loading buffer | / | 1 X | Sigma-Aldrich |
| Amersham Protran nitrocellulose membranes (0.20 µm-pore size) | / | / | GE Healthcare |
| Mini-PROTEAN TGX Stain Free Gels 4-15%, | #4568086 | / | Bio-Rad |
| Ladder Kaleidoscope | #1610375 | / | Bio-Rad |
| EveryBlot BlockingBuffer | #12010020 | / | Bio-Rad |
| anti-rabbit IgG-HRP-linked | / | 1/2000 | GE Healthcare |
| Secondary HRP-linked antibodies | / | 1/2000 | Jackson Laboratory |
| Clarity Western ECL Substrate | #170-5060 | / | Bio-Rad |
| ChemiDoc MP Imaging System | / | / | Bio-Rad |
| Immobilon ECL | #WBULS0100 | / | Millipore-Sigma |
| GeneGnome XRQ Imaging System | / | / | Syngene |
| Poly-L-Ornithine | #P3655 | 1X | Millipore-Sigma |
| Nunclon Delta surface | #140685 | / | ThermoFisher |
| anti-Annexin V-AF647 antibody | #640912, | / | BioLegend |
| YO-PRO-1™ | #V13243 | / | ThermoFisher |
| Click-It EdU kit | #BCK-EdUFC647 | / | BaseClick |
| FcR blocking reagent | #130-059-901 | / | Miltenyi Biotec |
| TMT10 kit | #90110 | / | Thermo Fisher |
| nanoLC–MS/MS (RSLC U3000 + Exploris480 with FAIMS) | / | / | Thermo Fisher |
| HTRF IP-One Gq kit | #62IPAPE, | / | Cisbio, Revvity |
| HTRF Phospho-ERK1/2 kit | #64ERKPEG | / | Cisbio, Revvity |
| PheraStar | / | / | BMG, Labtech |
| Cal520-AM | #Cal-520® AM | 1,25 µM | AATBioquest |
| Fura-2AM | #F1201 | 6 µM | ThermoFisher |
| FluxORTM | #F20015 | / | ThermoFisher |
| FURA2-AM Lambda 421–equipped Olympus IX73 microscope with Zyla sCMOS camera | / | / | Olympus, Oxford instruments |
| FDSS µcell | / | / | Hamamatsu Photonics |

For compounds :

| **Name** | **Reference** | **Stock concentration** | **Dilution** | **Final concentration** | **Supplier** |
| --- | --- | --- | --- | --- | --- |
| ET-1 | E7764-10UG | 4µM (10µg/mL) | PBS+BSA 0,1% | Defined in the study | Sigma-Aldrich |
| ET-3 | E9137-10UG | 4µM (10µg/mL) | PBS+BSA 0,1% | Defined in the study | Sigma-Aldrich |
| IRL-1620 | SCP0135-500UG | 100 µM | DMSO | Defined in the study | Sigma-Aldrich |
| BQ-788 | HY-15894A | 10 mM | DMSO | Defined in the study | MedChemExpress |
| hOSM | 300-10 | 10 µg/mL | PBS+BSA 0,1% | 10 ng/mL | Peprotech |
| hLIF | 300-05 | 10 µg/mL | PBS+BSA 0,1% | 10 ng/mL | Peprotech |
| hCNTF | 450-13 | 10 µg/mL | PBS+BSA 0,1% | 10 ng/mL | Peprotech |
| hBMP2 | 120-02C | 10 µg/mL | PBS+BSA 0,1% | 10 ng/mL | Peprotech |
| hBMP4 | 120-05 | 10 µg/mL | PBS+BSA 0,1% | 10 ng/mL | Peprotech |
| hBMP6 | 120-06 | 10 µg/mL | PBS+BSA 0,1% | 10 ng/mL | Peprotech |
| hIFNβ | 300-02BC | 10 µg/mL | PBS+BSA 0,1% | 10 ng/mL | Peprotech |
| hIFNγ | 300-02 | 10 µg/mL | PBS+BSA 0,1% | 10 ng/mL | Peprotech |
| TDI-011536 | HY-150042 | 5 mM | DMSO | 5 µM | MedChemExpress |

**Table S2**: List of patient specimens

| **Patient samples name** | **Patient gender** | **Patient Age** | **Diagnosis**  **(subype, grade)** | **IDH1 status** | **Extracted from biological samples** | **Use for which experiments (EDNRA/EDNRB)** | **IF/IHC** |
| --- | --- | --- | --- | --- | --- | --- | --- |
| Gb39 | F | 50 | GB | IDH WT | Protein | Western Blot |  |
| Gb40 | F | 81 | GB | IDH WT | RNA, protein | RT-qPCR, Western Blot |  |
| Gb43 | M | 52 | GB | IDH WT | RNA, protein | RT-qPCR, Western Blot |  |
| LGG321 | M | 28 | A2 | IDH1R132H | RNA, protein | RT-qPCR, Western Blot |  |
| LGG330 | M | 37 | A2 | IDH1R132H | RNA, protein | RT-qPCR, Western Blot |  |
| LGG250 | F | 40 | A2/3 | IDH1R132H | RNA, protein | RT-qPCR, Western Blot |  |
| LGG348 | F | 25 | A3 | IDH1R132H | RNA, protein | RT-qPCR, Western Blot, Cryosections | EDNRB/APOE |
| LGG309 | F | 61 | A3 | IDH1R132H | RNA | RT-qPCR |  |
| LGG309 (2) | F | 61 | A3 | IDH1R132H | RNA | RT-qPCR |  |
| LGG356 | M | 39 | A3 | IDH1R132H | RNA, protein | RT-qPCR, Western Blot |  |
| LGG322 | M | 52 | O2/3 | IDH1R132H | RNA | RT-qPCR |  |
| LGG346 | M | 45 | O2/3 | IDH1R132H | RNA, protein | RT-qPCR, Western Blot, Cryosections | EDNRB/IDH1R132H, Ctrl |
| LGG358 | M | 43 | O2 ou O3 | IDH1R132H | RNA, protein | RT-qPCR |  |
| LGG351 | M | 60 | O3 | IDH1R132H | RNA, protein | RT-qPCR, Western Blot |  |
| LGG357 | M | 29 | O2 (/O3) | IDH1R132H | RNA, protein | RT-qPCR, Western Blot, Cryosections | EDNRB/IDH1R132H |
| LGG357bis | M | 29 | O2 (/O3) | IDH1R132H | RNA | RT-qPCR | EDNRB/APOE ; EDNRB/OLIG2 |
| LGG359 | F | 62 | O3 | IDH1R132H | RNA, protein | RT-qPCR, Western Blot |  |
| LGG361 | M | 40 | O3 | IDH1R132H | Protein | Western Blot, Cryosections | EDNRB/IDH1R132H, Ctrl |
| LGG362 | M | 25 | O3 | IDH1R132H | Protein | Western Blot |  |
| LGG318 | M | 26 | A2 | IDH1R132H | Protein | Western Blot |  |
| LGG309 | F | 61 | A3 | IDH1R132H | Protein | Western Blot |  |
| LGG355 | M | 44 | A4 | IDH1R132H | Protein | Western Blot |  |
| LGG244 | M | 40 | A2 | IDH1R132H | Sections | Cryosections | EDNRB/OLIG2 |
| Gb34-A | M | 60 | GB | IDH WT | Protein | Western Blot |  |
| LGG 180 (180-1) | F | 35 | O2 | IDH1R132H | Protein | Western Blot |  |
| LGG184 (184-O) | F | 38 | A2 | IDH1R132H | Protein | Western Blot |  |
| LGG187 (187-1) | M | 26 | A2 | IDH1R132H | Protein | Western Blot |  |
| LGG182 (182) | M | 25 | A2/3 | IDH1R132H | Protein | Western Blot |  |
| LGG190 (190-2) | M | 42 | A2/3 | IDH1R132H | Protein | Western Blot |  |
| LGG189 (189-2) | F | 27 | A3 | IDH1R132H | Protein | Western Blot |  |
| LGG93 | N.A | N.A | O3 | IDH1R132H | Protein | Western Blot |  |
| LGG185 (185) | M | 39 | A4 | IDH1R132H | Protein | Western Blot |  |
| LGG56 (56) | M | 33 | A4 | IDH1R132H | Protein | Western Blot |  |
| LGG85 (85) | M | 38 | A4 | IDH1R132H | Protein | Western Blot |  |
| LGG316 | M | 59 | O2 | IDH1R132H | Protein | Western Blot |  |
| Meningioma | N.A | N.A | / | / | Protein | Western Blot |  |
| Human Brain | N.A | N.A | / | / | Protein | Western Blot |  |

(A= Astrocytomas, O=Oligodendrogliomas, GB = Glioblastomas)

**Table S3**: Glioma tissue microarray [1], [2].

|  | **Case no** | **Gender** | **Age** | **Diagnosis** | **WHO grade** | **Core 1** | **Core 2** |
| --- | --- | --- | --- | --- | --- | --- | --- |
| **1** | 3 | F | 37 | Anaplastic astrocytoma | 3 | 0 | 0 |
| **2** | 4 | F | 57 | Diffuse astrocytoma | 2 | 1 | 0 |
| **3** | 6 | M | 40 | Anaplastic oligodendroglioma | 3 |  |  |
| **4** | 7 | F | 65 | Oligodendroglioma | 2 |  |  |
| **5** | 26 | M | 57 | Control WM & GM |  | 1 | 1 |
| **6** | 27 | M | 34 | Anaplastic oligoastrocytoma | 3 | 1 | 0 |
| **7** | 33 | M | 54 | Glioblastoma | 4 | 1 | 2 |
| **8** | 34 | F | 67 | Anaplastic astrocytoma | 3 | 1 | 1 |
| **9** | 35 | M | 83 | Diffuse astrocytoma | 2 | 1 | 0 |
| **10** | 37 | F | 70 | Anaplastic oligodendroglioma | 3 |  |  |
| **11** | 38 | M | 29 | Oligodendroglioma | 2 |  |  |
| **12** | 55 | M | 28 | Control WM & GM |  | 1 | 0 |
| **13** | 56 | M | 49 | Anaplastic oligoastrocytoma | 3 | 1 | 2 |
| **14** | 60 | M | 65 | Glioblastoma | 4 | 0 | 1 |
| **15** | 61 | F | 31 | Anaplastic astrocytoma | 3 | 0 | 0 |
| **16** | 62 | M | 27 | Diffuse astrocytoma | 2 | 0 | 0 |
| **17** | 64 | F | 60 | Anaplastic oligodendroglioma | 3 |  |  |
| **18** | 76 | M | 38 | Control WM & GM |  | 0 | 0 |
| **19** | 77 | F | 61 | Anaplastic oligoastrocytoma | 3 | 1 | 0 |
| **20** | 80 | F | 29 | Anaplastic astrocytoma | 3 | 0 | 0 |
| **21** | 81 | M | 32 | Diffuse astrocytoma | 2 | 0 | 0 |
| **22** | 83 | F | 42 | Anaplastic oligodendroglioma | 3 |  |  |
| **23** | 84 | F | 65 | Oligodendroglioma | 2 |  |  |
| **24** | 92 | F | 24 | Anaplastic oligoastrocytoma | 3 | 1 | 0 |
| **25** | 93 | F | 65 | Glioblastoma | 4 | 2 | 2 |
| **26** | 94 | F | 46 | Anaplastic astrocytoma | 3 | 2 | 2 |
| **27** | 95 | M | 48 | Diffuse astrocytoma | 2 | 0 | 0 |
| **28** | 102 | M | 34 | Anaplastic oligoastrocytoma | 3 | 0 | 0 |
| **29** | 103 | F | 57 | Glioblastoma | 4 | 1 | 2 |
| **30** | 104 | M | 41 | Anaplastic astrocytoma | 3 | 0 | 1 |
| **31** | 105 | F | 42 | Diffuse astrocytoma | 2 | 0 | 0 |
| **32** | 106 | F | 34 | Anaplastic oligodendroglioma | 3 |  |  |
| **33** | 112 | F | 63 | Glioblastoma | 4 | 2 | 2 |
| **34** | 113 | F | 37 | Anaplastic astrocytoma | 3 | 1 |  |
| **35** | 114 | M | 26 | Diffuse astrocytoma | 2 | 1 | 1 |
| **36** | 115 | M | 42 | Anaplastic oligodendroglioma | 3 |  |  |
| **37** | 116 | M | 42 | Glioblastoma | 4 | 1 | 1 |
| **38** | 117 | F | 64 | Glioblastoma | 4 | 2 | 2 |
| **39** | 120 | M | 55 | Glioblastoma | 4 | 0 | 2 |
|  | | | | | |  |  |

Annotation: 0 = no vascular staining; 1 = minority of vessels stained; 2 = majority of vessels stained

**Table S4: Characteristics of the glioma cell lines**

| **Cell lines** | **Patient**  **(Sex-Age)** | **Tumor information** | **Grade**  **(WHO 2016)** | **IDH1**  **status** | **Miscellaneous information** | **Alterations** | **Publication status** |
| --- | --- | --- | --- | --- | --- | --- | --- |
| LGG275 | F40 | Recurrent primary Tumor (reoperation of 2014) | Grade II / III astrocytoma | IDH1R132H | Astrocytoma grade II according to histology astrocytoma high grade according to molecular annotation | ATRX lost Presence in 3 copies of EGFR, MYC~~,~~ Braf, c-Met, loss of one copy of CDKN2A | Augustus et al, 2021 |
| LGG336 | F40 | Recurrent primary tumor (reoperation of 2012) | Grade II / III astrocytoma | IDH1R132H | Astrocytoma grade II according to histology | c-Met and Braf 3 copies, loss of 9q | *Unpublished (Garcia et al, in preparation)* |
| LGG85 | M38 | Recurrent primary tumor (reoperation of 2012) | Grade IV astrocytoma | IDH1R132H | TMZ | c-Met neg | Leventoux et al, 2020 |
| LGG349 | F57 | Recurrent primary Tumor (reoperation of 2018) | Grade IV astrocytoma | IDH1R132H initially but mutation loss in culture | multiple cycles of PCV, radiotherapy + TMZ | ATRX maintained MGMT methylation, unmutated TERT | *Unpublished (Garcia et al, in preparation)* |
| BT138 | M50 | Recurrent primary Tumor (reoperation) | Grade III Oligodendroglioma (NOS) | IDH1R132H initially but mutation loss in culture | Tumor in the left frontal lobe |  | Koivunen et al, 2012 |
| BT237 | F43 | Recurrent primary Tumor (reoperation) | Grade III Oligodendroglioma | IDH1R132H | Tumor in the left frontal lobe |  | Koivunen et al, 2012 |
| BT054 | F49 | Recurrent primary Tumor | Grade III Oligodendroglioma | IDH1R132H | TMZ and radiotherapy Tumor in the right frontal lobe | MGMT methylation | Kelly et al, 2010; Luchman et al, 2012; Mazor et al, 2015; Yuan et al, 2018 |
| BT088 | M50 | Recurrent primary Tumor (reoperation) | Grade III Oligodendroglioma | IDH1R132H initially but mutation loss in culture | Tumor in the right frontal lobe Several cycles of PCV, radiotherapy+TMZ |  | Kelly et al, 2010; Luchman et al, 2012; Mazor et al, 2015; Yuan et al, 2018 |
| Gb4 | M53 | Primary Tumor | Grade IV Glioblastoma | IDH1 WT | Mesenchymal |  | Guichet et al, 2013  Guichet et al, 2015 |
| Gb5 | M64 | Primary Tumor | Grade IV Glioblastoma | IDH1 WT | GB with oligodendroglial component |  | Guichet et al, 2013  Guichet et al, 2016 |
| Gb7 | M52 | Primary Tumor | Grade IV Glioblastoma | IDH1 WT | Proneural |  | Guichet et al, 2013; Guichet et al, 2015 |
| Gb21 | F53 | Primary Tumor | Grade IV Glioblastoma | IDH1 WT | Giant Cell GB |  | Guichet et al, 2016;  Guelfi et al, 2021 |

**Table S5 :** List of RT-qPCR primers

| **Gene** | **Forward Primer** | **Reverse Primer** |
| --- | --- | --- |
| ACTB | GGACTTCGAGCAAGAGATGG | AGCACTGTGTTGGCGTACAG |
| **EDNRA** | TCAAGATGGAAACCCTTTGC | ATTGAGCCATTGCTGGGTAG |
| **EDNRB** | ATGACGCCACCCACTAAGAC | GAACACAAGGCAGGACACAA |
| CD44 | CAATAGCACCTTGCCCACAAT | AATCACCACGTGCCCTTCTATG |
| CTGF | CAGCATGGACGTTCGTCTG | AACCACGGTTTGGTCCTTGG |
| OLIG1 | CGCAGAGAGTTTTCGCTCTT | GCGGTTGGTTTTCGTTTTTA |
| OLIG2 | GACAAGCTAGGAGGCAGTGG | CGGCTCTGTCATTTGCTTCT |
| MKI67 | CCCCCACCAGAACTAACAGA | ACTTTGATGCCCTCATCACC |
| STAT3 | CAGCAGCTTGACACACGGTA | AAACACCAAAGTGGCATGTGA |
| NOTCH1 | TCCACCAGTTTGAATGGTCA | CGCAGAGGGTTGTATTGGTT |
| KCNN3 | CTGCCGCCAAAATAAACATT | GCCTGGCACAAGCTTTCTAC |

**Table S6****:** List of antibodies

| **Name** | **Species** | **Reference** | **Manufacturer** | **Dilution** |
| --- | --- | --- | --- | --- |
| EDNRA | Rabbit | ab178454 | Abcam | 1:1000 |
| EDNRB | Sheep | AF4496-SP | R&D Systems | 1:500 |
| RB49 | Mouse | CEA | from Dr Boquet & Dr Herbet | 1:500 |
| OLIG1 | Goat | AF2417 | R&D Systems | 1:300 |
| OLIG2 | Mouse | MABN50 | Sigma | 1:500 |
| ASCL1 | Rabbit | ab211327 | Abcam | 1:100 |
| GFAP | Rabbit | Z0334 | Dako | 1:5000 |
| AQP4 | Rabbit | 16473-1-AP | Proteintech | 1:500 |
| APOE | Rabbit | EP1374Y | Abcam | 1:1000 |
| pSMAD1/5 | Rabbit | 9516 | Cell Signalling | 1:800 |
| IDH1R132H | Mouse | DIA−H09 | Dianova | 1:100 |
| CD44-PE | Rabbit | 130-110-394 | Miltenyi Biotec | 1:100 |
| CTGF | Rabbit | Ab209780 | Abcam | 1:500 |
| pSTAT3 | Rabbit | 9145S | Cell Signalling | 1:1000 |
| STAT3 | Rabbit | A19566 | ABClonal | 1:1000 |
| pERK1/2 | Rabbit | 9910 | Cell Signalling | 1:1000 |
| ERK1/2 | Rabbit | 9102 | Cell Signalling | 1:1000 |
| KCNN3/SK3 | Rabbit | H-45 | Santa Cruz Biotechnology | 1:100 |
| KCNN2/SK2 | Rabbit | APC-028 | Alomone Labs | 1:200 |
| α-actin | Rabbit | A2066 | Sigma-Aldrich | 1:5000 |
| β-actin | Mouse | 8H10D10 | Novus | 1:2500 |

**Supplementary references for Table S3**

[1] L. Zhang *et al.*, “Pleiotrophin promotes vascular abnormalization in gliomas and correlates with poor survival in patients with astrocytomas.,” *Sci. Signal.*, vol. 8, no. 406, p. ra125, Dec. 2015, doi: 10.1126/scisignal.aaa1690.

[2] S. N. Popova *et al.*, “Subtyping of gliomas of various WHO grades by the application of immunohistochemistry.,” *Histopathology*, vol. 64, no. 3, pp. 365–79, Feb. 2014, doi: 10.1111/his.12252.
