# Supplementary material for: EDNRB‐dependent endothelin signaling reduces proliferation and promotes proneural‐to‐mesenchymal transition in gliomas
Source: Mol Oncol. 2026 Apr 23:10.1002/1878-0261.70223. Online ahead of print. doi: 10.1002/1878-0261.70223 (PMC13398683; doi:10.1002/1878-0261.70223)
Supplement: Supplementary file 15 — Data S1. Supplementary Material and Methods. [file MOL2-9999-0-s015.docx]

**Supplementary Material and Methods**

**Additional patient cell line and patient samples.** The Gb5 glioblastoma stem cell line previously described [1] was included for EDNRA and EDNRB mRNA expression exploration. The tumor microarrays (TMAs) are the same as described previously [2], [3]. These TMAs, generated from human brain tumors collected retrospectively at Uppsala University Hospital, include low- and high-grade gliomas as well as non-malignant brain tissue. EDNRA abundance was assessed by immunohistochemistry-based protein profiling using an anti-EDNRA antibody (AER-001, Alomone Labs).

**Public datasets (bulk and single-cell RNA-seq).** Expression of EDNRA and EDNRB were queried in bulk RNA-seq datasets (REMBRANDT, TCGA, CGGA) via the GlioVis portal [4], [5], [6], [7], [8] and in glioma single-cell RNA-seq datasets [9], [10], [11], [12] using the Gephart lab GBMseq browser and the Broad Institute Single Cell Portal [13].

**Single-cell RNA sequencing analysis of LGG275 cell line.** LGG275 (+GFs) grown in PolyHEMA-coated flasks were dissociated with trypsin-EDTA, filtered (40 μm), purified on a 15% Percoll gradient, and resuspended at 1,000 cells/μL in PBS/0.04% BSA. Viability was assessed with a CASY meter. Single-cell suspensions were processed on a Chromium Controller (10x Genomics) using the Chromium Single Cell 3’ v3.1 kit, following the manufacturer’s protocol. Libraries were sequenced on an Illumina NovaSeq 6000 (28 bp Read1, 10 bp I7, 10 bp I5, 90 bp Read2). Data were processed with Cell Ranger v6.1.1 (GRCh38), including a second --force-cells run to remove ambient RNA, aggregated with cellranger aggr, and visualized in Loupe Browser. Sequencing data are available at GEO (GSE263796). A detailed analysis of the single-cell RNA-seq results from LGG275 will be reported elsewhere [14].

**Spatial transcriptomics data analysis in glioblastoma from Ravi et al.** The 10X Visium spatial transcriptomic dataset from Ravi et al. was downloaded from Datadryad (<https://datadryad.org/stash/dataset/doi:10.5061/dryad.h70rxwdmj>) and analyzed using the Seurat R package. From the full cohort, samples UKF248T, UKF 259T and UKF269 were selected for our analyses since they showed the most interesting spatial expression levels and patterns of all samples. Spatial clustering was performed using the BayesSpace algorithm (<https://www.nature.com/articles/s41587-021-00935-2>). The endothelial cell signature was defined via the following genes: PECAM1, CDH5 and KDR, whereas for the pericyte signature the markers PDGFRB, MYH11, NOTCH3, HIGD1B, RGS5 and ACTA2 were considered. Signatures were calculated using the AddModuleScore function from the Seurat package. Spatial expression visualizations were generated via the SpatialFeaturePlot function and violin plots were created via the VlnPlot function.

**EDNRA DNA methylation sequencing bioinformatic analysis**

For EDNRA whole genomic locus methylation and specific promoter methylation, data was retrieved from TCGA-GBMLGG DNA methylation 450k profiles in function of IDH_ mutation found using <https://xenabrowser.net/> (accessed 17 Nov 2025) [15]. R Statistical Software (Version 4.5.2; R Core Team, 2025) and [RStudio](https://cran.r-project.org/) (Version 4.5.2; Posit Team, 2025) were used for EDNRA methylation level assessment and data visualization was performed using the ggplot2 package [16].

**References**

[1] P.-O. Guichet *et al.*, “Cell death and neuronal differentiation of glioblastoma stem-like cells induced by neurogenic transcription factors,” *Glia*, vol. 61, no. 2, pp. 225–239, Feb. 2013, doi: 10.1002/glia.22429.

[2] S. N. Popova *et al.*, “Subtyping of gliomas of various WHO grades by the application of immunohistochemistry.,” *Histopathology*, vol. 64, no. 3, pp. 365–79, Feb. 2014, doi: 10.1111/his.12252.

[3] L. Zhang *et al.*, “Pleiotrophin promotes vascular abnormalization in gliomas and correlates with poor survival in patients with astrocytomas.,” *Sci. Signal.*, vol. 8, no. 406, p. ra125, Dec. 2015, doi: 10.1126/scisignal.aaa1690.

[4] R. L. Bowman, Q. Wang, A. Carro, R. G. W. Verhaak, and M. Squatrito, “GlioVis data portal for visualization and analysis of brain tumor expression datasets,” *Neuro. Oncol.*, vol. 19, no. 1, pp. 139–141, Jan. 2017, doi: 10.1093/neuonc/now247.

[5] Y. Gusev, K. Bhuvaneshwar, L. Song, J. C. Zenklusen, H. Fine, and S. Madhavan, “Data descriptor: The REMBRANDT study, a large collection of genomic data from brain cancer patients,” *Sci. Data*, vol. 5, Aug. 2018, doi: 10.1038/sdata.2018.158.

[6] C. Hutter and J. C. Zenklusen, “The Cancer Genome Atlas: Creating Lasting Value beyond Its Data,” Apr. 05, 2018, *Cell Press*. doi: 10.1016/j.cell.2018.03.042.

[7] K. Tomczak, P. Czerwińska, and M. Wiznerowicz, “The Cancer Genome Atlas (TCGA): An immeasurable source of knowledge,” 2015, *Termedia Publishing House Ltd.* doi: 10.5114/wo.2014.47136.

[8] Z. Zhao *et al.*, “Chinese Glioma Genome Atlas (CGGA): A Comprehensive Resource with Functional Genomic Data from Chinese Glioma Patients,” *Genomics Proteomics Bioinformatics*, vol. 19, no. 1, pp. 1–12, Feb. 2021, doi: 10.1016/j.gpb.2020.10.005.

[9] S. Darmanis *et al.*, “Single-Cell RNA-Seq Analysis of Infiltrating Neoplastic Cells at the Migrating Front of Human Glioblastoma,” *Cell Rep.*, vol. 21, no. 5, pp. 1399–1410, Oct. 2017, doi: 10.1016/j.celrep.2017.10.030.

[10] A. S. Venteicher *et al.*, “Decoupling genetics, lineages, and microenvironment in IDH-mutant gliomas by single-cell RNA-seq,” *Science (1979).*, vol. 355, no. 6332, Mar. 2017, doi: 10.1126/science.aai8478.

[11] I. Tirosh *et al.*, “Single-cell RNA-seq supports a developmental hierarchy in human oligodendroglioma,” *Nature*, vol. 539, no. 7628, pp. 309–313, Nov. 2016, doi: 10.1038/nature20123.

[12] C. Neftel *et al.*, “An Integrative Model of Cellular States, Plasticity, and Genetics for Glioblastoma,” *Cell*, vol. 178, no. 4, pp. 835-849.e21, Aug. 2019, doi: 10.1016/j.cell.2019.06.024.

[13] L. Tarhan, J. Bistline, J. Chang, B. Galloway, E. Hanna, and E. Weitz, “Single Cell Portal: an interactive home for single-cell genomics data,” Jul. 17, 2023. doi: 10.1101/2023.07.13.548886.

[14] L. Garcia *et al.*, “Multi-omics characterization of IDH-mutant astrocytoma-derived cell lines reveals NOTCH-regulated plastic quiescent astrocyte-like state,” *bioRxiv*, p. 2025.12.30.696808, Jan. 2025, doi: 10.64898/2025.12.30.696808.

[15] K. A. Hoadley *et al.*, “Cell-of-Origin Patterns Dominate the Molecular Classification of 10,000 Tumors from 33 Types of Cancer,” *Cell*, vol. 173, no. 2, pp. 291-304.e6, Apr. 2018, doi: 10.1016/j.cell.2018.03.022.

[16] H. Wickham, *ggplot2*. Cham: Springer International Publishing, 2016. doi: 10.1007/978-3-319-24277-4.
