# Supplementary material for: EDNRB‐dependent endothelin signaling reduces proliferation and promotes proneural‐to‐mesenchymal transition in gliomas
Source: Mol Oncol. 2026 Apr 23:10.1002/1878-0261.70223. Online ahead of print. doi: 10.1002/1878-0261.70223 (PMC13398683; doi:10.1002/1878-0261.70223)
Supplement: Supplementary file 16 — Data S2. Supplementary Figure legend. [file MOL2-9999-0-s011.docx]

**Supplementary File**

**Supplementary Figures legends:**

**Figure S1 | EDNRB is preferentially expressed in astrocyte-like glioma cells (related to Fig. 1). A.** Single-cell RNA-seq analysis of glioblastoma (Darmanis et al., 2017) showing cell-type composition and EDNRA/EDNRB expression patterns [69]. (*Left*) UMAP plots display major cell populations (color-coded) from tumor core, periphery, and healthy tissue. (*Right*) EDNRA expression is predominantly enriched in vascular/mural cells, with a small subpopulation of tumor cells also expressing EDNRA (red arrow). EDNRB shows highest expression in astrocytes and is also present in tumor and vascular cells. Bar graphs indicate log₂ counts per million (mean ± SEM) for each cell type. **B.** Single-cell RNA-seq from three studies showing EDNRB expression by glioma cell subtype. (*Left*) Venteicher et al., 2017 (IDH–mutant astrocytomas): heterogeneous expression across clusters of tumors pinpointing a preferential expression in tumor subpopulations. Color scale: red = high, white = low. (*Middle*) Tirosh et al., 2016 (IDH–mutant oligodendrogliomas): preferential expression in AC-like than oligodendrocyte-like cells. (*Right*) Neftel et al., 2019 (IDH–wild-type GB): preferential expression of EDNRB in astrocyte-like (AC-like) cells. **C.** (*Left*) Correlation between EDNRB expression and lineage-associated genes in diffuse IDH1-mutant astrocytomas and oligodendrogliomas, based on scRNA-seq data from Venteicher et al., 2017 [9]. Correlation coefficients were manually retrieved from the study’s online database. In astrocytomas, EDNRB shows positive correlations with astrocytic markers and negative correlations with oligodendrocytic genes, indicating an astrocyte-like profile—similar to that observed in GB. (*Right*) Right: correlation of EDNRB with APOE, SOX9, AQP4, OLIG1, OLIG2, SOX8 in ODG, ASTRO, and GB, from Suvà group datasets ([9], [10], [13]). **D**. Representative immunofluorescence of astrocytomas (AIII) and oligodendrogliomas (OII) stained for EDNRB (*purple*), APOE or OLIG2 (*green*), nuclei (Hoechst, *blue*). *Yellow* arrowheads: EDNRB⁺/APOE⁺ cells; *white* arrows: EDNRB⁻/OLIG2⁺ or EDNRB⁺/OLIG2⁻ cells. Scale bars: 20 µm. **E**. Quantification of APOE⁺ and OLIG2⁺ cells among EDNRB⁺ cells in AIII and OII shows most EDNRB⁺ cells are APOE⁺, not OLIG2⁺. IDH Isocitrate Dehydrogenase, AC-like Astrocyte-like cells, EDNRA Endothelin Receptor Type A, EDNRB Endothelin Receptor Type B, O Oligodendrogliomas (ODG), A Astrocytomas (ASTRO), GB Glioblastomas, APOE Apolipoprotein E, SOX2/4/8/9/10 SRY-Box Transcription Factors 2/4/8/9/10, AQP4 Aquaporin 4, OLIG1 Oligodendrocyte Lineage Transcription Factor 1, OLIG2 Oligodendrocyte Lineage Transcription Factor 2.

**Figure S2 |** **EDNRB is the predominant endothelin receptor and is expressed at the cell surface of diffuse glioma cultures (related to Fig. 2).** **A**. RT–qPCR validation of EDNRA (*Left*) and EDNRB (*Right*) expression (confirming RNA-seq from Fig. 2A) in 11 patient-derived glioma lines and one additional GB line (Gb5) (Guichet et al, 2013), cultured with (+GFs) or without (–GFs) growth factors. +GFs: mean_EDNRA_ ±SD=0,00006±0,00009, mean_EDNRB_±SD=0,004±0,003 -GFs:mean_EDNRA_ ±SD=0,0009±0,002, mean_EDNRB_ ±SD=0,02±0,02;-GFs: mean_EDNRB_ fold-increase= 5,71). **B**. Western blot of EDNRA in glioma lines (T98G, U87, LGG275, Gb4) and CHO cells (Chinese Hamster Ovarian). EDNRA was detected only in EDNRA-overexpressing CHO cells (positive control) and was absent from glioma lines, consistent with Fig. 2A. **C**. Immunofluorescence of LGG275 cells stained for EDNRB (red; RB49 antibody targeting an extracellular epitope (Herbet et al, 2018)) under fixed or live conditions. Nuclei were counterstained with Hoechst (*blue*). EDNRB localizes to the cell surface, with stronger labeling in –GF conditions. Scale bar, 10 µm. **D.** Flow cytometry of EDNRB surface expression in fixed and live LGG275 cells cultured ±GFs, using RB49 and a commercial antibody (R&D Systems, Cat. #AF4496). Bar graphs show mean ± SD of % EDNRB⁺ cells (n = 3). Statistics: ANOVA with Tukey’s test; *p <0.05, **p <0.01, ****p <0.0001. RT-qPCR Reverse Transcription Quantitative Polymerase Chain Reaction, EDNRA Endothelin Receptor Type A, EDNRB Endothelin Receptor Type B, GF Growth Factor, Rendomab-B49.

**Figure S3** **|** **EDNRB expression defines a low-proliferative, astrocyte-like cell population in gliomas. (related to Fig. 1-2) A.** WebCSEA (Web-based Cell-type-Specific Enrichment Analysis, UTHealth) cell-type enrichment of EDNRB-high LGG275 cells from scRNA-seq analyses of LGG275 cells. Top 100 differentially expressed genes (p <0.05) were analyzed, Top 20 general cell types displayed on the graph are revealing significant enrichment for astrocytes (*purple*; *red* dashed line = adjusted p <0.01). **B.** scRNA-seq analysis of LGG275 cells. UMAPs showing EDNRB, MKI67, and PCNA expression (*pink* = high, *grey* = low). Violin plots compare proliferation marker levels between EDNRB-high and EDNRB-negative cells. Tests: Benjamini–Hochberg adjusted p-values. EDNRB-high cells show reduced proliferation. **C.** scRNA-seq analysis of glioblastoma (Neftel et al., 2019) [13] (*Left)* t-SNE plot showing major cell types (color-coded). (*Middle*) Single-cell expression maps of EDNRA, EDNRB, PDGFRA (OPC marker), and MKI67 (proliferation marker). Tumor cells preferentially express EDNRB, but a small population also expresses EDNRA (red arrow). (*Top right*) Dot plot summarizing EDNRA and EDNRB expression levels and percentages across the four main cell types in GB. (*Bottom right*) Pearson correlation matrix between lineage-associated genes (EDNRB, OLIG1, OLIG2, ASCL1) and proliferation markers (MKI67, PCNA, CCND1, CCND2, CCNB1), showing minimal association of EDNRB with proliferation compared to oligodendrocyte-lineage genes. EDNRA Endothelin Receptor Type A, EDNRB Endothelin Receptor Type B, GF Growth Factor, PDGFRA Platelet-Derived Growth Factor Receptor Alpha, OLIG1 Oligodendrocyte Lineage Transcription Factor 1, OLIG2 Oligodendrocyte Lineage Transcription Factor 2, ASCL1 Achaete-Scute Family BHLH Transcription Factor 1 (MASH1), MKI67 Marker of proliferation Ki-67, PCNA Proliferating Cell Nuclear Antigen, CCND1 Cyclin D1, CCND2 Cyclin D2, CCNB1 Cyclin B1, UMAP Uniform Manifold Approximation and Projection.

**Figure S4** **|** **Further characterization of ET-1 effects on migration and cell death (related to Fig. 3)** **A.** Flow cytometry analysis of cell death in LGG275 cells treated with ET-1 or ET-3. Cell death was assessed using (left) YO-PRO/Propidium Iodide (PI) or (right) Annexin-V/Hoechst 33342 staining. H₂O₂ (600 µM) served as a positive control (not shown). Data are shown as mean ± SD (n = 3 independent experiments for ET-1 in both assays; n = 3 independent experiments for ET-3 with YO-PRO/PI; n = 1 for ET-3 with Annexin-V/Hoechst). ns, not significant (ANOVA with Tukey’s post-hoc test). **B.** Time-lapse analysis of LGG275 migration after ET-1 treatment (10 nM). Migration velocity was tracked over 22 h, showing a significant increase from 10 h onward. Data are shown as mean± SD with n=100 cells per condition. ANOVA with Tukey’s post-hoc test (*p <0.05 to ***p <0.001). **C.** Mean square displacement (MSD) of LGG275 cells measured over successive 4 h intervals (2–22 h) after ET-1 treatment, showing increased displacement and migration efficiency over time. Data are shown as mean± SD with n=100 cells per condition over 110 min. **D.** Migration trajectories of individual LGG275 cells in consecutive 4 h intervals following ET-1 treatment, showing a progressive displacement from the point of origin over time. Each plot-to-origin displayed 50 color-coded traces. **E.** GSEA of RNA-seq data from ET-1–treated LGG275 cells showing significant enrichment of the *WU_Cell_Migration* gene set. NES, nominal *p*-value, and FDR *q*-value are indicated. ET-1 Endothelin 1, GSEA Gene Set Enrichment Analysis, NES Normalized Enrichment Score, FDR False Discovery Rate.

**Figure S5 | ET-1 drives astrocytic/mesenchymal transcriptional reprogramming in IDH1-mutant and IDH–wildtype glioma cells revealed by RNA-seq (related to Fig. 4).
A.** Pathway enrichment (EnrichR) from 65 DEGs in LGG275 cells after ET-1 treatment (n = 3 independent experiments) [76]. Bars show –log₁₀(p) values; Fisher’s exact test (one-sided hypergeometric over-representation test) implemented in EnrichR with significance: p <0.05 (*), <0.01 (**), <0.001 (***). **B–C.** Analyses of Bulk RNA-seq of ET-1–treated glioma lines: *(B)* IDH1-mutant (LGG336, BT237) and *(C)* IDH–wildtype GB (Gb4, Gb7, Gb21) (n=1 per condition). Data are shown as TPM and FDR-adjusted p-values are reported for descriptive purposes*. Top panels*: Fold-change of lineage-associated genes (oligodendrocytic/proneural = purple; astrocytic/mesenchymal-like = green). Negative fold-change values were obtained by applying the transformation –1/(linear fold-change) to achieve graphical symmetry when representing downregulated genes. *Bottom panels*: GSEA for each line; all processes FDR q <0.25. Significance: p <0.05 (*), <0.01 (**), <0.001 (***). ET-1 Endothelin 1, DEG Differentially Expressed Gene, TPM Transcripts Per Million, GSEA Gene Set Enrichment Analysis, FDR False Discovery Rate.

**Figure S6 | ET-1 induces shifts in the proteomic profile associated with mesenchymal transition signatures in LGG275 cells (related to Fig. 4). A**. Gene Ontology (GO) term enrichment analysis of proteomic profiles from LGG275 cells treated with ET-1 versus vehicle, based on five independent experiments and analyzed by 1D annotation enrichment with Fisher’s exact test. Orange bars indicate positive enrichment, black bars negative enrichment. **B**. Scatter plot of proteins quantified by TMT-based mass spectrometry (ET-1 vs vehicle) (same as Fig 4H). The x-axis shows the log₂ ratio (ET/V) and the y-axis the iBAQ value. Proteins in orange correspond to 26 of the 28 proneural-to-mesenchymal transition (PMT) markers defined by Chanoch-Myers et al., 2022 [14]; 73% (19 out of 26) of these are upregulated following ET-1 treatment, consistent with a mesenchymal-like shift. ET-1 Endothelin 1, TMT Tandem Mass Tag, iBAQ Intensity-Based Absolute Quantification.

**Figure S7 | Signaling stimulated by endothelins in glioma cell lines (related to Fig. 5) A**. ET-1–induced Ca²⁺ responses in BT237 IDH1-mutant oligodendroglioma cells (related to Fig. 5 C). *Left*: Intracellular Ca²⁺ fluxes (Cal-520 AM) in control, ET-1, or ET-3–treated cells cultured with (+GFs) or without (–GFs) growth factors. Data shown as mean ± SD, n = 3 independent experiments; ANOVA/Tukey; ****p <0.0001. *Right*: Dose–response curves for the EDNRB agonist IRL-1620 and the EDNRB antagonist BQ-788 plus EC₈₀ ET-1 (4 nM) with or without GFs. Curves: non-linear regression; one representative of three experiments shown. **B.** ERK1/2 phosphorylation induction is masked in LGG275 under +GFs in presence of endothelins (ET-1, ET-3). Data are represented as mean ± SD (n = 3 independent experiments); ANOVA/Tukey was used for statistical analyses, ns= non-significant and EDNRB activation (Non-linear regression dose–response curves from one representative of three experiments). **C**. ET-1–stimulated STAT3 signaling in glioma cell lines. *Top:* Western blot of phosphorylated STAT3 (pSTAT3, Tyr705), total STAT3, and β-actin in BT237 (n = 3), Gb4 (n = 2), and Gb7 (n = 2) cells treated with ET-1 or vehicle control. *Bottom*: Quantification of pSTAT3 normalized to total STAT3; values are expressed as arbitrary units (A.U.) relative to control (set to 1). **D–E.** Expression of KCNN2/SK2 and KCNN3/SK3 in LGG275 cells. RNA (TPM, RNA-seq; n = 2) and protein (Western blot) levels in cells cultured with (+GFs) or without (–GFs) growth factors. Actin was used as a loading control. **F.** Correlation of EDNRB expression with KCNN2 and KCNN3 in gliomas. Scatter plots from the CGGA dataset (n = 1,016) showing Pearson’s correlation between EDNRB and KCNN2 (top) or KCNN3 (bottom) expression. r² and p-values =0, (two-sided) from GlioVis database. **G.** Validation of FluxOR™ probe for detecting K⁺ fluxes in LGG275 cells (related to Fig. 5 H). *Left*: Normalized fluorescence intensity (Max–Min) after treatment with the K⁺ ionophore nigericin (10 µM or 5 µM) under +GFs or –GFs conditions.
*Right*: K⁺ flux induced by the Ca²⁺ ionophore ionomycin (10 µM) with or without the SK-channel blocker apamin (1 µM), under +GFs or –GFs conditions. Data: mean ± SD, n = 2 independent experiments; ANOVA/Tukey. ****p <0.0001; ***p <0.001; **p <0.01; ns: not significant. **H.** Apamin-sensitive K⁺ currents in LGG275 cells (+GFs). Fold-change in membrane current amplitude (normalized to control) recorded under whole-cell voltage-clamp with intracellular free Ca²⁺ buffered at ~1 µM (PCa6) to activate SK channels. Spontaneous Ca²⁺-dependent K⁺ currents were significantly reduced by apamin (100 nM), a selective SK channel blocker. Mean ± SD (n =13 cells). Unpaired t-test; *p <0.05. ET-1 Endothelin 1, ET-3 Endothelin 3, GF Growth Factor, ERK1 Extracellular Signal-Regulated Kinase 1 (MAPK3), ERK2 Extracellular Signal-Regulated Kinase 2 (MAPK1), CGGA Chinese Glioma Genome Atlas, KCNN2/SK2 Potassium Calcium-Activated Channel Subfamily N Member 2/ Small Conductance, KCNN3/SK3 Potassium Calcium-Activated Channel Subfamily N Member 3/Small Conductance, STAT3 Signal Transducer and Activator of Transcription 3.

**Figure S8 | Validation of TDI activating YAP pathway in diffuse glioma cells (related to Fig. 6).**
Western blot analysis of YAP and CTGF (full-length ~37 kDa and cleaved ~21 kDa) in LGG275 (**A)** and Gb7 cells (**B**) treated with TDI (5 µM) or vehicle (Ctrl). β-actin served as a loading control. Right panels: densitometric quantification of CTGF normalized to β-actin, expressed as arbitrary units (A.U.) with Ctrl set to 1. Data from n=2 (LGG275) and n=3 independent experiments (Gb7). CCN2/CTGF Cellular Communication Network Factor 2 / Connective Tissue Growth Factor, YAP Yes-Associated Protein.

**Figure S9 | EDNRA expression increases with glioma grade. A.** *(Left)* Immunohistochemistry on tumor microarrays (TMA [91-92]) showing EDNRA in control brain (white + grey matter) and gliomas (oligodendrogliomas, astrocytomas, glioblastomas). Scale bar = 600 µm. *(Right)* Quantification of EDNRA integrated signal intensity (A.U.) in control and tumor samples (grades 2–4) from 39 patients. Data were analyzed by ANOVA with Tukey’s post-hoc test; *p <0.05. Scale bar= 600 µm **B.** Representative immunofluorescence images of oligodendroglioma (OII) and glioblastoma (GB) stained for EDNRA (*green*), CD31 (*red*), and nuclei (Hoechst, *blue*). Merged images (*left*) show EDNRA localization near CD31⁺ endothelial cells, with insets highlighting colocalization (*white* arrows, OII; *yellow* arrows, GB). EDNRA staining is stronger and more widespread around vessels in GB. Scale bars = 10 µm. **C.** Single-cell expression of EDNRA and EDNRB in vascular and vascular-associated tumor cells. Violin plots showing normalized EDNRA (*top*) and EDNRB (*bottom*) expression across major brain cell types from purified vascular fractions of glioma tissues (scRNA-seq; Xie et al., 2024 [97]). These plots were adapted from the interactive resource available at<https://scatlas.shinyapps.io/human_bbb_and_btb/>. EDNRA is enriched in mural and vascular-associated tumor cells, whereas EDNRB is mainly detected in astrocytes. The absence of EDNRB in tumor cells here likely reflects the isolation strategy (MACS with CD31⁺ and PDGFRβ⁺ selection), which enriches for vascular/perivascular populations while excluding most tumor cells. CD31 Cluster of Differentiation 31 (PECAM-1, Platelet Endothelial Cell Adhesion Molecule 1), MACS Magnetic-Activated Cell Sorting, PDGFRB Platelet-Derived Growth Factor Receptor Beta.

**Figure S10 | Spatial transcriptomic mapping of endothelin receptors in glioblastoma.**
**A**. Violin plots showing normalized transcript counts for EDNRA and EDNRB in three glioblastoma specimens (248_T, 259_T, 269_T; data from Ravi et al.[99]). EDNRB is consistently more highly expressed than EDNRA. **B**. Spatial transcriptomic expression maps (Ravi et al.[99]) for the same specimens, displaying EDNRA and EDNRB expression, endothelial and pericyte marker signatures, and the “Cluster 7” gene set from Wang et al. (2023) [98] with or without EDNRA. For each patient: (top left) H&E-stained section; (top middle) spatial EDNRA/EDNRB expression; (top right) spatial cell-type and Cluster 7 signature scores (blue-to-red gradient). (bottom left) spatial clustering of Visium spots; (bottom right) violin plots of EDNRA/B expression, cell types, and Cluster 7 signature scores across spatial clusters. EDNRA shows a more restricted pattern than EDNRB and is enriched in regions co-locating with vessel markers. These analyses indicate that tumor cells with the Cluster-7 signature and EDNRA expression share features with endothelial cells and pericytes. Scale bars of H&E stained sections = 100 µm.

**Figure S11 | Endothelin receptor gene expression across human glioma datasets.** mRNA levels (log₂) for *EDNRA* and *EDNRB* were obtained from the GlioVis portal (accessed 3 Jan 2025) and plotted as box-and-whisker plots (median, interquartile range with 95% confidence interval); each dot represents one tumor sample. Unless otherwise stated, statistical significance was assessed in GlioVis using one-way ANOVA with Tukey’s post-hoc test; * p <0.05, ** p <0.01, *** p <0.001, ns = not significant. **A**. REMBRANDT microarray cohort: non-tumoral brain (n=28) vs oligodendroglioma (ODG, n=67), astrocytoma (AST, n=147), and glioblastoma (GB, n=219). Across all samples (N = 537), EDNRB expression was significantly higher than EDNRA (mean±SD: 10,03±1,14 vs 7,77±0,71, p<0.001). **B**. TCGA pan-glioma RNA-seq cohort stratified by WHO tumor grade II (n=226), III (n=244), and IV (n=150). Across all samples (N = 667), EDNRB expression was significantly higher than EDNRA (mean±SD :12,03 ± 1,14 vs. 8,47 ± 1,19, p < 0.001). **C**. CGGA RNA-seq cohort: primary (n=651) vs recurrent (n=333) gliomas. In primary tumors, EDNRB expression was significantly higher than EDNRA (mean±SD : 5,09±1,59 vs 1,92±1,19, p<0.001*)*. EDNRB expression appears to decrease with increasing malignancy (REMBRANDT: mean±SD from grade II-IV 10,52±1,17 to 9,65±0.98, Tukey HSD p<0,001, pairwise t-test p=2,9.10^-8^; TCGA: mean±SD from grade II-IV 12,77±1,22 to 11,75±1.10, Tukey HSD p<0,001, pairwise t-test p=7,2.10^-13^; CGGA: mean±SD EDNRB (primary) 5,09±1,59 vs (recurrence*)* 4,64±1,48, Tukey HSD p<0,001, pairwise t-test p=6,9.10^-5^; from grade II-IV 5,57±1,50 to 4,51±1,47, Tukey HSD p<0,001, pairwise t-test p=1,7.10^-18^), while EDNRA expression appears to increase (REMBRANDT: from grade II-IV 7,69±0,74 to 7,94±0,73, HSD p<0,05, pairwise t-test p=5,6.10^-2^; TCGA: from grade II-IV 8,27±1,23 to 8,93±0,94, Tukey HSD p<0,001, pairwise t-test p=2,1.10^-7^; CGGA: from grade II-IV 1,75±1,15 to 2,26±1,24, Tukey HSD p<0,001, pairwise t-test p=1,9.10^-7^). **D.** TCGA pan-glioma Kaplan–Meier survival analysis of patients stratified by high vs. low EDNRA or EDNRB expression (cutoff: median) over 200 months. Statistical significance obtained in GlioVis is displayed on the graph. HSD Tukey's Honestly Significant Difference test, REMBRANDT Repository for Molecular Brain Neoplasia Data, TCGA The Cancer Genome Atlas, CGGA Chinese Glioma Genome Atlas.

**Figure S12 | EDNRA gene exhibits higher methylation levels in IDH-mutant gliomas.** DNA methylation β-values (Illumina array) were obtained from the UCSC Xena Browser and visualized as box plots. Box-and-whisker plots are shown. The box represents the interquartile range (IQR), the center line indicates the median, and whiskers represent the confidence interval around the median. **A**. EDNRA whole genomic locus methylation data was retrieved from TCGA-GBMLGG DNA methylation 450k profiles in function of IDH_ mutation found using <https://xenabrowser.net/> (accessed 17 Nov 2025) (Hoadley et al, 2018). EDNRA average global gene methylation was higher in IDH-mutant compared to IDH-wildtype gliomas patients (N=1153). Statistics: Welch’s t-test p=0.008. **B**. EDNRA specific promoter methylation data was retrieved from same database using R showing higher EDNRA promoter methylation in IDH-mutant (n=412) compared to IDH-wildtype gliomas (n=105); Wilcoxon test p=1,29.10^-54^. Each dot represents one methylation value obtained from a single patient. EDNRA Endothelin Receptor Type A, TCGA The Cancer Genome Atlas, GBM Glioblastomas, LGG Low-Grade Gliomas, IDH Isocitrate Dehydrogenase.
